# Computer-Assisted Dental Implant Placement Following Free Flap Reconstruction: Virtual Planning, CAD/CAM Templates, Dynamic Navigation and Augmented Reality

**DOI:** 10.3389/fonc.2021.754943

**Published:** 2022-01-28

**Authors:** Santiago Ochandiano, David García-Mato, Alba Gonzalez-Alvarez, Rafael Moreta-Martinez, Manuel Tousidonis, Carlos Navarro-Cuellar, Ignacio Navarro-Cuellar, José Ignacio Salmerón, Javier Pascau

**Affiliations:** ^1^ Servicio de Cirugía Oral y Maxilofacial, Hospital General Universitario Gregorio Marañón, Madrid, Spain; ^2^ Instituto de Investigación Sanitaria Gregorio Marañón, Madrid, Spain; ^3^ Departamento de Bioingeniería e Ingeniería Aeroespacial, Universidad Carlos III de Madrid, Madrid, Spain

**Keywords:** virtual surgical planning, 3D printing, computer-aided surgery, free flaps, dental implants, dynamic navigation, augmented reality, static navigation

## Abstract

Image-guided surgery, prosthetic-based virtual planning, 3D printing, and CAD/CAM technology are changing head and neck ablative and reconstructive surgical oncology. Due to quality-of-life improvement, dental implant rehabilitation could be considered in every patient treated with curative intent. Accurate implant placement is mandatory for prosthesis long-term stability and success in oncologic patients. We present a prospective study, with a novel workflow, comprising 11 patients reconstructed with free flaps and 56 osseointegrated implants placed in bone flaps or remnant jaws (iliac crest, fibula, radial forearm, anterolateral thigh). Starting from CT data and jaw plaster model scanning, virtual dental prosthesis was designed. Then prosthetically driven dental implacement was also virtually planned and transferred to the patient by means of intraoperative infrared optical navigation (first four patients), and a combination of conventional static teeth supported 3D-printed acrylic guide stent, intraoperative dynamic navigation, and augmented reality for final intraoperative verification (last 7 patients). Coronal, apical, and angular deviation between virtual surgical planning and final guided intraoperative position was measured on each implant. There is a clear learning curve for surgeons when applying guided methods. Initial only-navigated cases achieved low accuracy but were comparable to non-guided freehand positioning due to jig registration instability. Subsequent dynamic navigation cases combining highly stable acrylic static guides as reference and registration markers result in the highest accuracy with a 1–1.5-mm deviation at the insertion point. Smartphone-based augmented reality visualization is a valuable tool for intraoperative visualization and final verification, although it is still a difficult technique for guiding surgery. A fixed screw-retained ideal dental prosthesis was achieved in every case as virtually planned. Implant placement, the final step in free flap oncological reconstruction, could be accurately planned and placed with image-guided surgery, 3D printing, and CAD/CAM technology. The learning curve could be overcome with preclinical laboratory training, but virtually designed and 3D-printed tracer registration stability is crucial for accurate and predictable results. Applying these concepts to our difficult oncologic patient subgroup with deep anatomic alterations ended in comparable results as those reported in non-oncologic patients.

## Introduction

Head and neck tumor treatment entails an irreversible anatomical distortion and a loss of essential functions such as chewing, swallowing, or phonation. Facial contour disfigurement is also common, especially when adjuvant radiotherapy is required. Therefore, functional restoration of the oral cavity is one of the main challenges for head and neck surgeons. In this context, microsurgical free flaps enable the three-dimensional (3D) repair of orofacial defects on an individual basis to restore lost tissue.

In 1991, Urken (1) stated the main functional objectives for the reconstruction of the oral cavity (1): primary restoration of bone continuity with rigid fixed vascularized bone (2), immediate positioning of osseointegrated implants to ensure rapid rehabilitation of occlusion (3), placement of thin and pliable tissue for reconstruction of the floor of the mouth and tongue, and (4) restoration of soft tissue sensitivity: labial competence and restoration of sensation in intraoral tissue.

In 1988, Riediger (2) was the first author that fitted delayed implants in an iliac crest microsurgical flap. One year later, Urken et al. (3) were the pioneer in fitting implants immediately during the hard tissue reconstructive procedure with DCIA flap (deep circumflex iliac artery, the iliac crest flap). Since then, shape reconstruction using flaps and function restoration with implant-supported prostheses have become well-established methods.

As stated by Schoen et al. (4), in any curative cancer treatment, the placement of dental implants should be evaluated. Roumanas et al. (5) studied chew impairment after tumor resection. They concluded that microsurgical reconstruction and conventional dental restoration contribute to chewing function recovery. However, this study showed a statistically significant improvement when dental rehabilitation was based on osseointegrated implants. It is well reported that quality of life improves after dental implant-supported rehabilitation in oncologic patients in terms of self-assessed masticatory ability, social and psychological disability (6), or good to excellent speech intelligibility and aesthetics (7).

Prosthetic-based implant placement in oncologic patients poses major challenges for the surgeon because these patients have small mouth openings, flat reconstructed ridges, reduced tongue mobility and lip seal, thickened and retracted mucosa, xerostomia, skin scars, etc. (8). An ideal prosthetic rehabilitation should provide appropriate support, retention, and stability, preventing soft tissue injuries; this is why we consider that implant-based dental restoration is the only alternative to reestablish cosmesis and function in oncologic patients.

Smolka et al. (9) reported a significant difference between successful osseointegration and the ability of implants to provide valuable and functional restoration. A 92% osseointegration success rate fell to 42% in the functional evaluation owing to factors such as lack of patient cooperation or implant malpositioning. In our department, Cuesta-Gil et al. (10) found that 4.4% of malpositioned implants are due to a critical lack of parallelism, excessive angulation, or else lingual or vestibular deviation, demonstrating that adequate implant placement is crucial for long-term prosthetic success. More recently, we reported less than 2% of malpositioned, non-load-bearing, osseointegrated implants in oncologic patients (11). Clark et al. (12) estimated that about 7% of complications might be related to implant malposition. Nowadays, the goal is not only to load all the osseointegrated implants but also to place the fixtures so that they are guided by the prosthesis in an accurate and biomechanically ideal position. Poor implant positioning would lead to biological complications due to the inability to maintain proper hygiene, peri-implantitis, unfavorable mechanical load, and, finally, loss of implant at an early stage (13).

In head and neck oncology, virtual surgical planning (VSP) and image-guided surgery (IGS) have become widely accepted methods to improve resection and reconstruction reproducibility, speed, and accuracy (14). Based on CT or MRI data, this concept could also be applied to implant surgery in cancer patients to overcome the aforementioned challenges. Our dental implant placement philosophy has changed in numerous ways. From placing freehand implants in maximum bone volume and density, we moved to prosthetically driven surgery, guided surgery, and finally, computer-assisted surgery.

Computer-assisted implant surgery (CAIS) was introduced in 1995 by Fortin et al. (15), seeking an increased precision and accuracy with a particular interest in complex oncologic reconstructed patients. Several authors have demonstrated the CAIS concept in treating oncologic patients reconstructed with free flaps (8, 16, 17).

The prosthetically driven implant placement method is based on 3D image reconstruction and planning of virtual implant placement in the optimal position. Gargallo-Albiol et al. (18) classified implant navigation surgery as dynamic and static. The ideal plan is transferred to the actual surgical site through a custom-made template in the case of static CAIS (sCAIS) or through real-time tracking and guidance of the surgical drill in dynamic CAIS systems (dCAIS) (19).

Regarding the type of drilling and implant placement, static navigation can also be divided into fully guided (FG) and half-guided (HG) implant surgery. During FG navigation, drilling and insertion of the implants are performed through the rigid guide, whereas during HG navigation the drilling procedure is guided while the implants are inserted freehand without the guide in place. Furthermore, depending on the type of surgical visibility and guide support, we can differentiate between open and closed guided (flapless) or mucosa, bone, and tooth-crown-supported guided navigation. Both approaches, static and dynamic, rely on how the presurgical information is translated into the surgical procedure, offering different advantages and limitations.

When comparing dynamic and static guidance versus freehand placement, the literature shows consistently improved accuracy for the guided procedure (20). When studying guided techniques, Kaewsiri et al. (21) and Mischkowski et al. (22) concluded that dynamic navigation provided higher accuracy than the static guide system. Therefore, any guided method yielded better results than freehand implant placement techniques (17). Dynamic navigation is accurate, is useful in edentulous and mouth-restricted opening patients, and allows intraoperative updates. Static-guided surgery is simple, is cheaper, allows flapless surgery, and produces excellent results in dentate patients.

Another cutting-edge technology used in computer-aided surgery is augmented reality (AR). AR enables the surgeon to visualize virtual information from the patient (e.g., virtual surgical plan or medical images) overlaid on the surgical field (23). Clinical application reports of AR in implantology are scarce. Pellegrino et al. (24) presented the feasibility of adopting AR to facilitate the use of dynamic navigation for dental implantology and evaluated AR’s accuracy compared to dynamic navigation in two cases.

To the best of our knowledge, this is the first time where the advantages of combining both guided techniques, static and dynamic navigation, are presented through a workflow that comprises virtual surgical planning, patient-specific 3D-printed tools, dynamic guidance based on real-time optical tracking, and augmented reality visualization. We have already applied these technologies to improve the surgical management of craniosynostosis (25–27). Our research has demonstrated that integrating these solutions into the surgical workflow has a positive impact on surgical outcomes, increasing the reproducibility and efficiency of the interventions (28). Therefore, we hypothesize that virtual prosthetically driven implant placement planning could be accurately translated to our oncologic reconstructed patients by combining static navigation, dynamic navigation, and AR visualization.

## Materials and Methods

### Population

Eleven head and neck reconstructed oncologic patients (5 epidermoid carcinomas, 1 mucoepidermoid carcinoma, 1 adenoid cystic carcinoma, and 4 ameloblastomas), 9 males and 2 females, ages ranging from 18 to 84 years, with different bone and soft tissue defects, were treated with virtually planned and fully guided dental implant placement to restore form and function. Surgical reconstructions included free and regional flaps, 2 iliac crest free flaps, 2 double-barrel fibula flaps, 4 conventional osteocutaneous fibula flaps, 1 ALT with vastus lateralis flap, 1 radial forearm flap, and 1 pectoralis major after a failed fibula flap. Two patients received adjuvant radiotherapy. **Table 1** summarizes the characteristics of each patient included in this study.

**Table 1:** Characteristics of the patients participating in this study.

Localization and tumor	Surgery	Reconstruction	Guided method	Implants	Prosthesis	Results
Mandibular right body and ramus ameloblastoma	Segmental mandibulectomy	Fibula flap	sCAIS	2 flap, 1 failed	Fixed screw retained	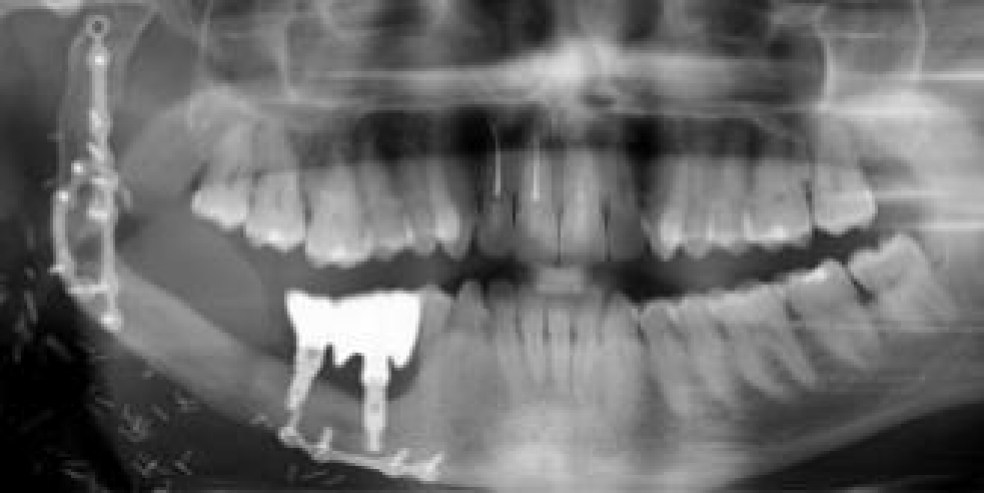
Right mandibular body epidermoid carcinoma	Segmental mandibulectomy and neck dissection	Double-barrel fibula flap	dCAIS	3 flap	Fixed screw retained	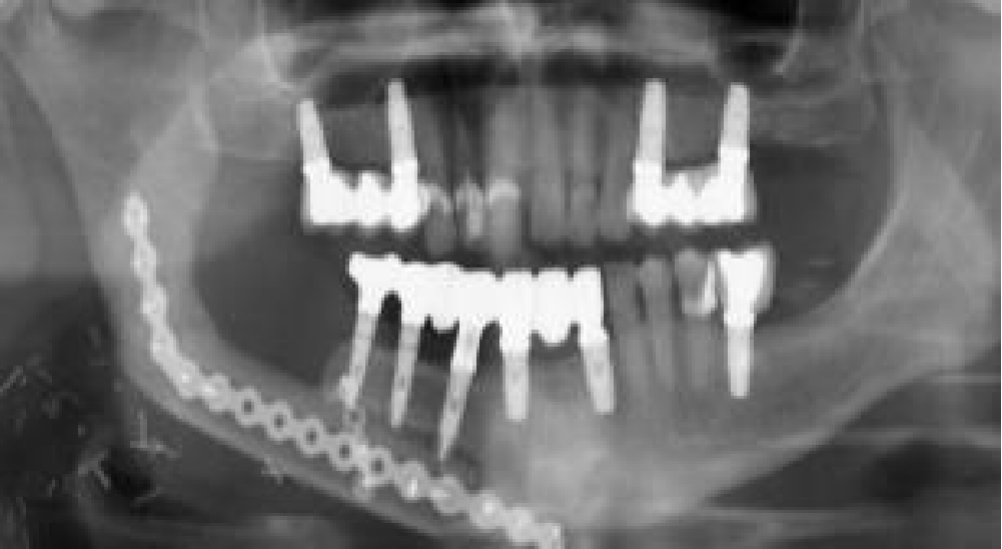
Left mandibular body epidermoid carcinoma	Segmental mandibulectomy and Neck dissection	Iliac crest free flap	dCAIS	2 flap	Fixed screw retained	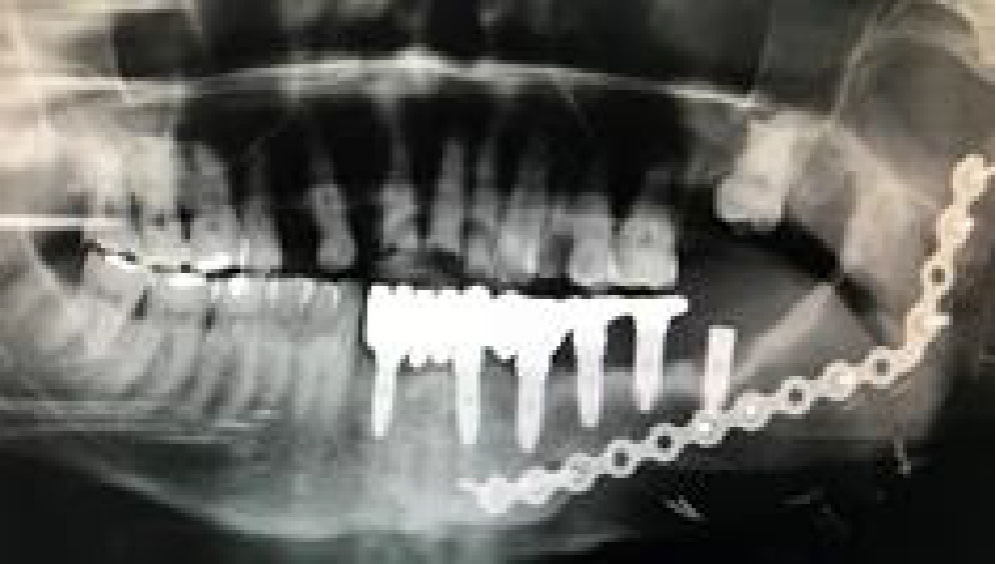
				2 Mdb		
Left mandibular body ameloblastoma	Segmental mandibulectomy	Fibula flap	dCAIS	4 flap	Fixed screw retained	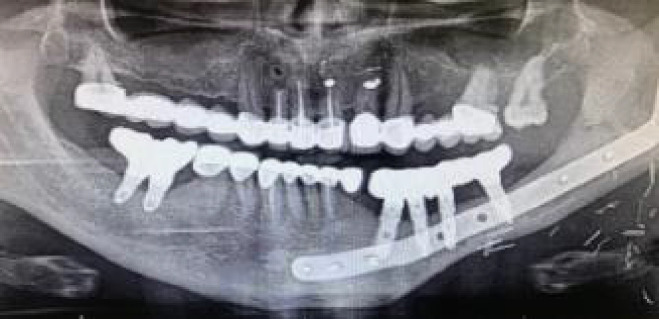
Right mandibular body ameloblastoma	Segmental mandibulectomy	Fibula flap	dCAIS and freehand	3 flap	Fixed screw retained	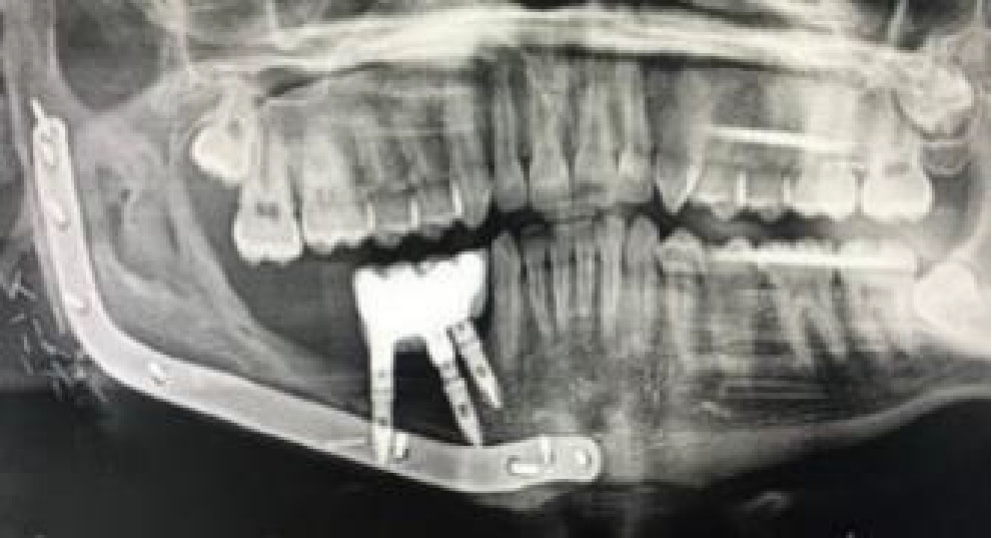
Hard palate adenoid cystic carcinoma	Central maxillectomy	Radial forearm flap	dCAIS and sCAIS	5 Mx	Fixed screw retained	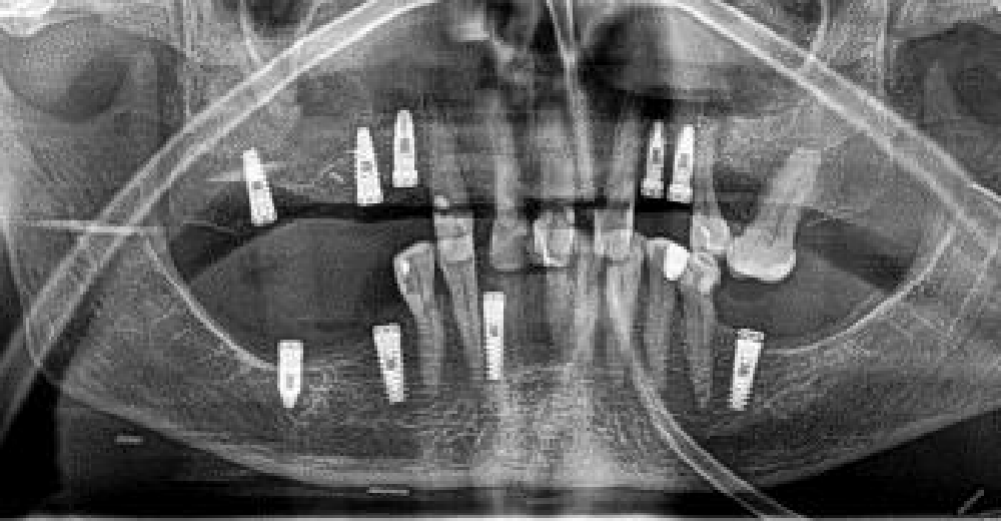
				4 Mdb		
Left maxilla tuberosity adenocarcinoma	IIb Brown maxillectomy	Iliac crest free flap	dCAIS and sCAIS	3 flap	Fixed screw retained	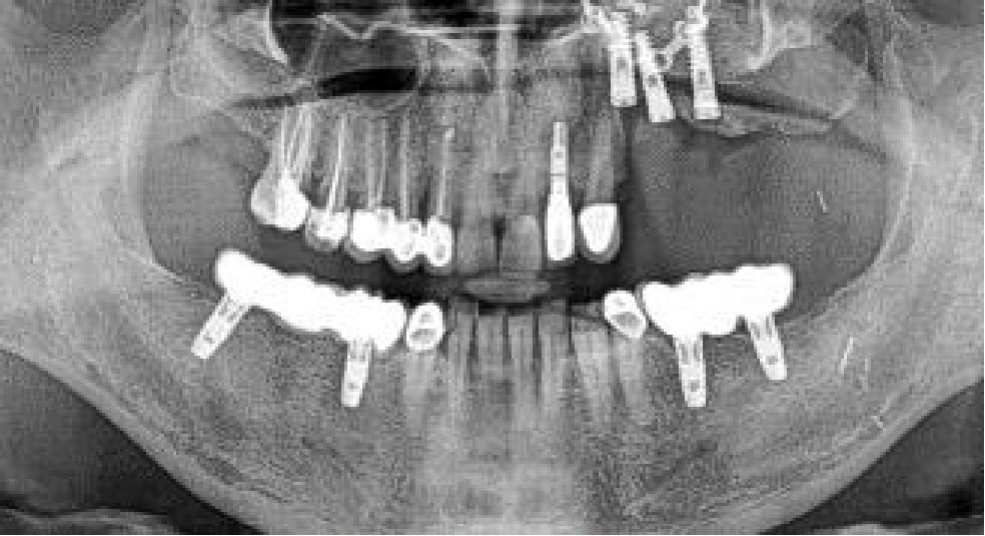
Left mandibular body epidermoid carcinoma	Segmental mandibulectomy and Neck dissection	Double-barrel fibula flap + 70 Gy	dCAIS and sCAIS	3 flap	Fixed screw retained (Pending)	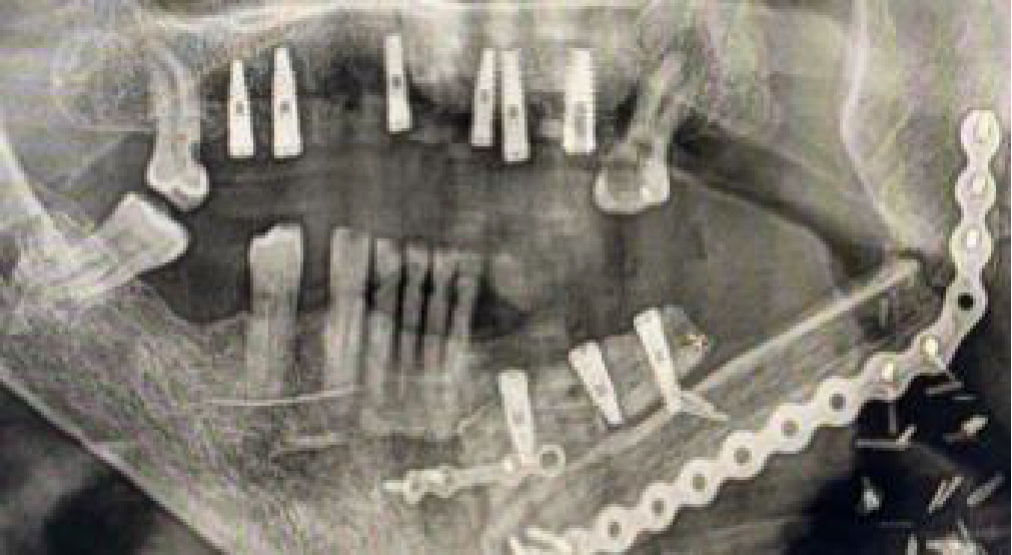
				6 mx		
Left mandibular body epidermoide carcinoma	Segmental mandibulectomy and Neck dissection	Fibula flap	dCAIS and sCAIS	3 flap	Fixed screw retained (pending)	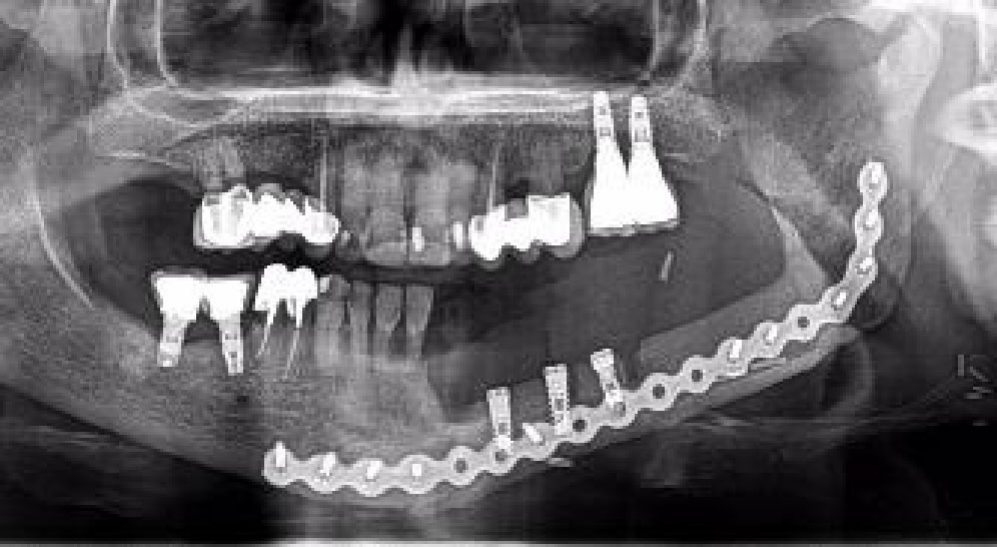
Right hemitongue epidermoid carcinoma	Hemiglosectomy, bilateral neck dissection	Alt+ Vastus Lateralis Flap + 70 Gy	dCAIS	6 mx	Fixed screw retained (pending)	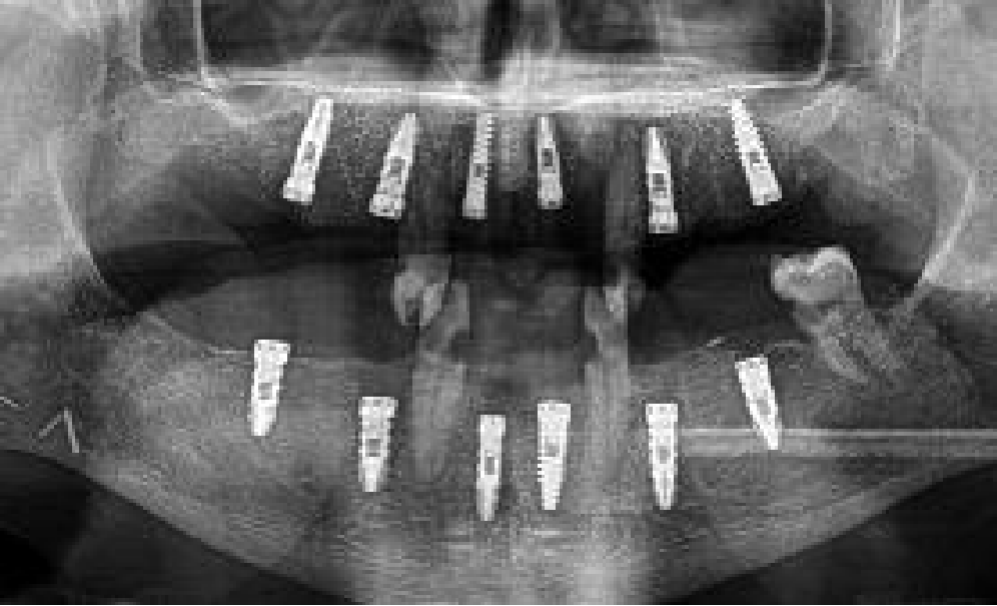
				6 mdb		
Left hemimandible ameloblastoma	Segmental mandibulectomy	Failed Fibula flap and pectoralis major	sCAIS	5 mx	Fixed screw retained (pending)	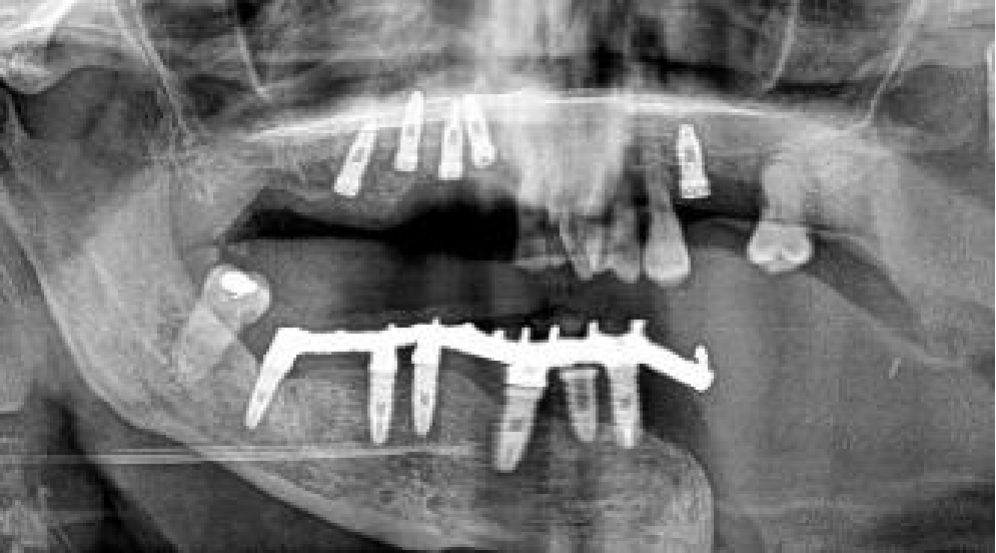

All patients signed an informed consent for study participation. The study was performed in accordance with the principles of the 1964 Declaration of Helsinki as revised in 2013 and was approved by the Research Ethics Committee at Hospital General Universitario Gregorio Marañón in Madrid.

### Treatment Protocol for Computer-Assisted Implant Surgery

Our treatment protocol follows these fundamental steps:

- Cone-beam computed tomography (CBCT) and patient plaster model scanning.
- Virtual planning: virtual dental prosthesis design, definition of prosthesis-guided implant location, and surgical guide design, including a modification for dynamic navigation (holes and sleeves for registration markers).
- Fabrication of surgical drilling guides on biocompatible resin with a 3D printer.
- Surgical-guided procedure. Static, dynamic, or mixed technique. Intraoperative verification with augmented reality
- Postoperative CBCT and analysis of position differences between planned and final results.

**Figure 1:**
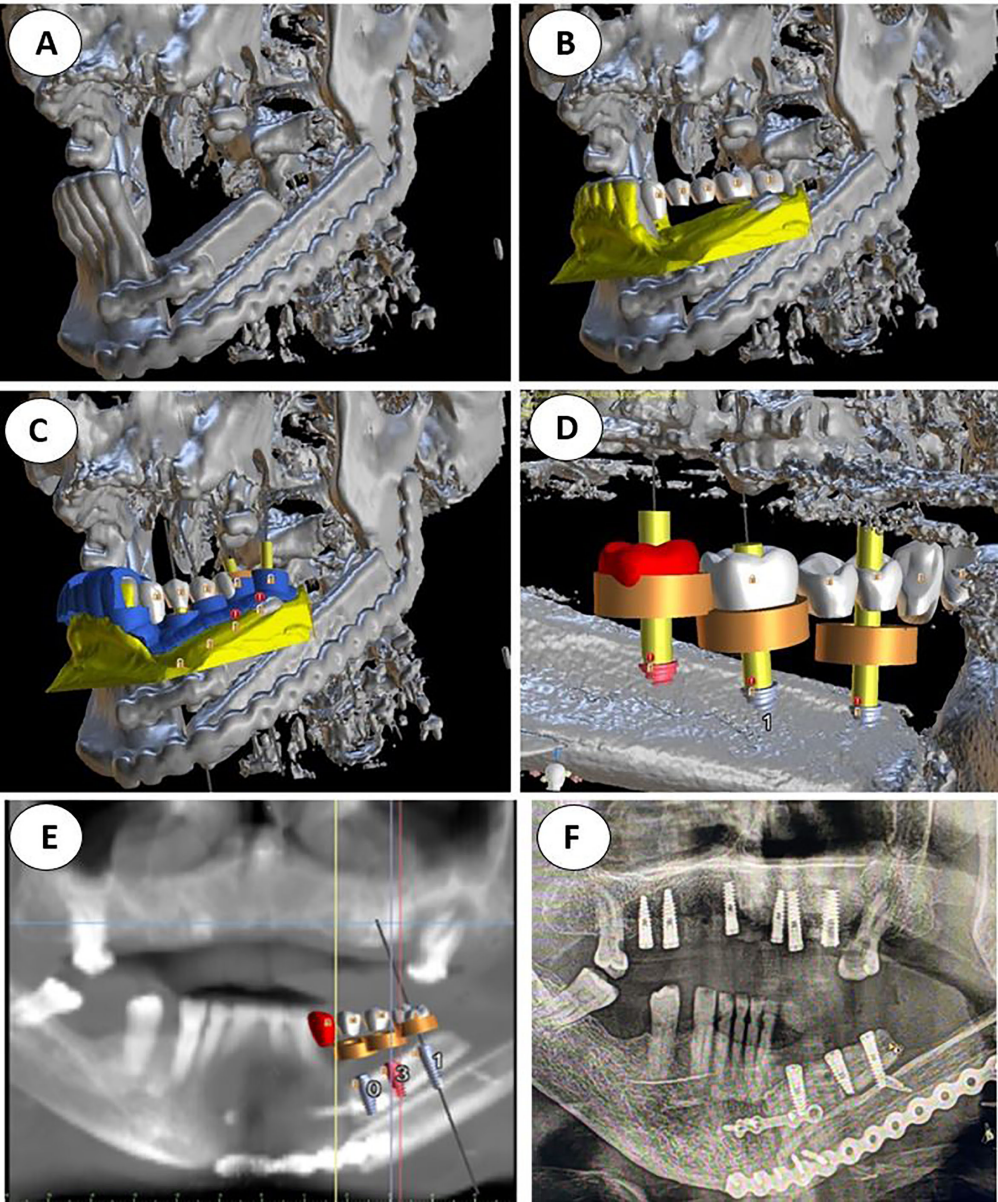
**(A)** Double barrel fibula flap CBCT 16 months after irradiation with 70 Gy. A basal reconstructive plate and a crestal miniplate. **(B)** Scanned lower jaw plaster model merged with the CBCT and virtual tooth design. **(C)** Prosthetically driven implant planning and in blue the teeth supported rigid splint designed with windows for insertion verification. **(D)** Lingual view seen from the floor of the mouth of the crestal fibula segment and the prosthetically driven implant placement. **(E)** VSP, preoperative implant planned position superimposed in the CBCT **(F)** Postoperative orthopantomogram.

A bimaxillary CBCT scan was acquired for each patient (**Figure 1A**). Then, impressions of both arches were taken with either silicone or alginate materials, and the plaster was scanned to obtain 3D digital models with a D700 3Shape^®^ scanner. These 3D models were manually aligned with the CBCT scan using anatomical landmarks. Then, VSP was performed on a computer workstation for optimal prosthetically guided dental implant placement using either Nobel Clinician-DTX^®^ studio implant licensed software or Blue Sky Bio^®^ open software **(** **Figure 1B**). VSP started with the virtual screw-retained prosthesis design. Once teeth are in the ideal occlusal position, implant locations were defined, focusing on achieving an appropriate angulation and depth while avoiding any interference with osteosynthesis screws. Finally, we ended with a virtual objective of treatment (**Figures 1C–F**). In all patients we placed Ticare^®^Osseous (Mozo-Grau, SA, Valladolid, Spain) and Ticare^®^Osseous Quattro (Mozo-Grau, SA, Valladolid, Spain).

**Figure 2:**
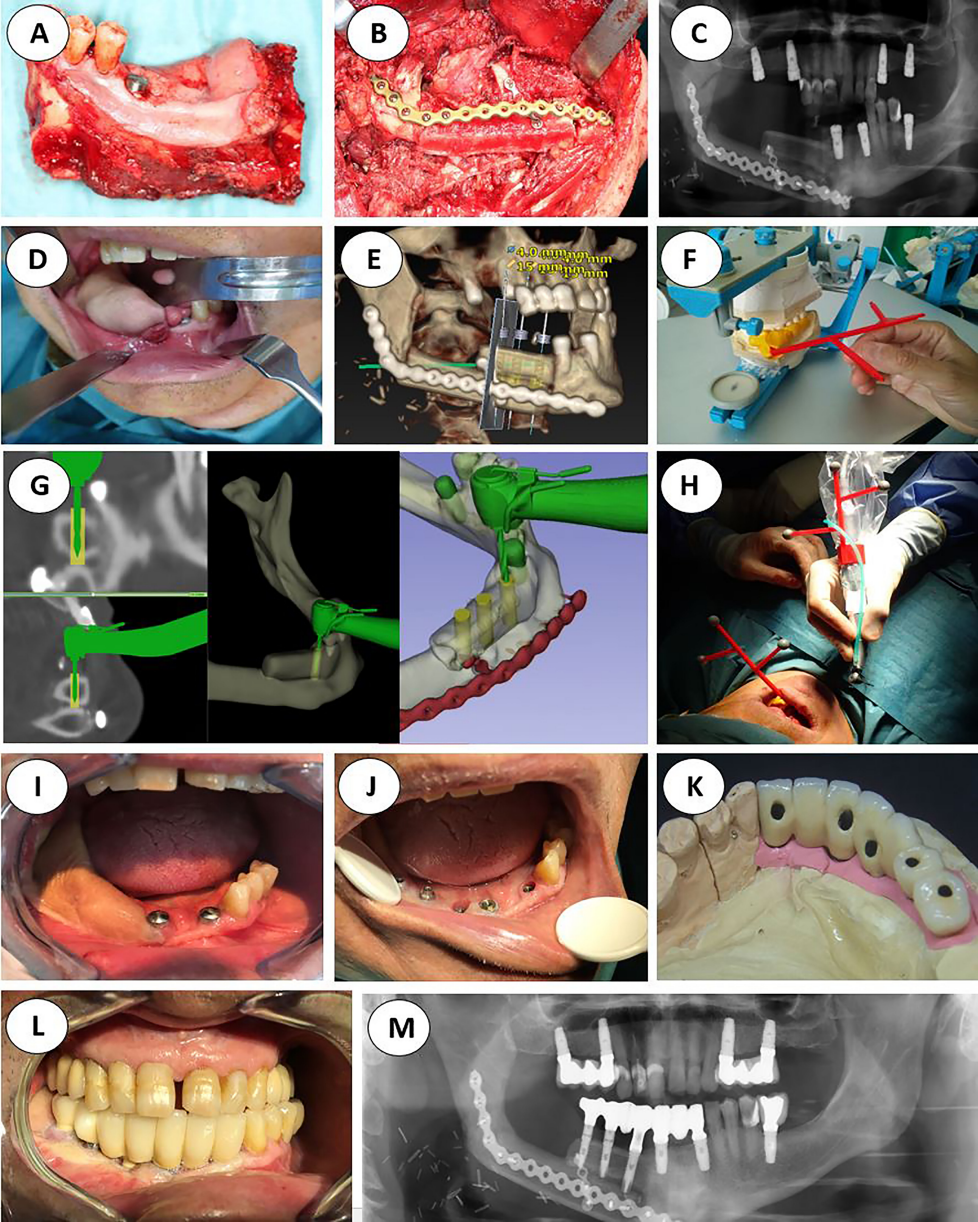
Initial protocol example, from the ablative surgery to the final orthopantomogram **(A)** Right segmental mandibulectomy. **(B)** Double-barrel fibula flap in place. **(C)** Postoperative orthopantomography. **(D)** Redundant fibula skin paddle. **(E)** Implant placement virtual surgical planning with Nobel Clinician-DivX software. **(F)** Teeth supported silicone jig holding the 3D-printed dynamic reference frame. **(G)** Intraoperative screen view of the navigated handpiece and the real-time drilling trajectory. **(H)** Handpiece and patient’s optical markers ready for dynamic navigation. **(I)** Still redundant skin paddle after implant surgery. **(J)** Intraoral view after vestibuloplasty and implant second phase. **(K)** Screw retained porcelain fused to metal fixed prosthesis lingual view. **(L)** Final occlusion. **(M)** Final panorex with the prosthesis in place.

Regarding virtual surgical planning translation to the surgical field, in our initial four patients (**Figure 2**) we followed a silicone jig tooth-supported dynamic navigated procedure. We built the virtual plan in the NobelClinician-DTX^®^ studio implant software (Nobel Biocare^®^, Zurich, Switzerland) and manually created a silicone tooth-retained jig to hold the 3D-printed dynamic reference frame by articulating upper and lower jaw plaster impressions on a semi-adjustable articulator. Registration was based on fiducial markers and in some previous foreign bodies attached to the oncologic patient, including *in-situ* osseointegrated implant heads or fixed osteosynthesis devices, screws, and reconstructive plates.

In the third patient, we encountered a problem with the stability of the silicone tooth-supported jig intraoperatively. This finding forced us to end that procedure in a conventional non-guided freehand way. From this third patient, we decided to stop using the silicone jig as the retention method for the dynamic reference frame.

**Figure 3:**
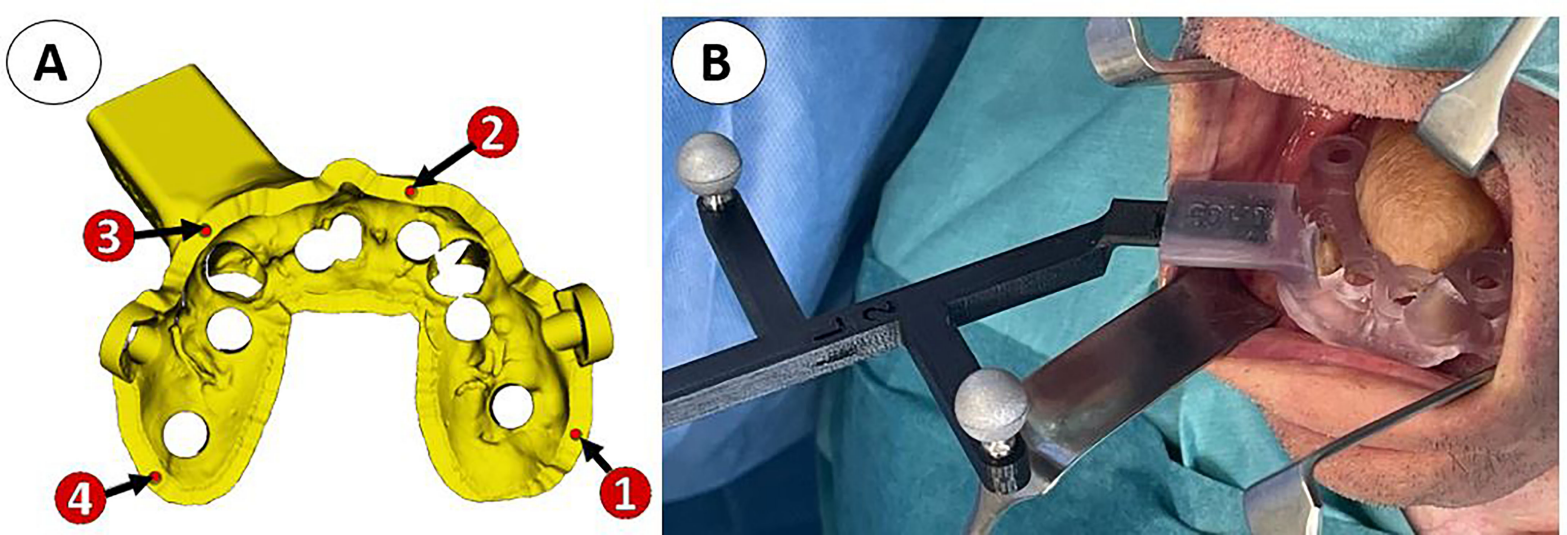
**(A)** Virtual model of the surgical guide with four pinholes (red) for intraoperative registration and a socket for the attachment of the dynamic reference frame. **(B)** 3D-printed biocompatible teeth-supported resin guide holding the dynamic reference frame during computer-assisted surgery in a right hemotingue epidermoid carcinoma reconstructed by means of an ALT with vastus lateralis free flap.

Therefore, for the subsequent seven cases, rigid resin tooth-supported guides were designed with 3D CAD (Blue Sky Bio software) and manufactured by 3D printing technology, seeking intraoperative stability. With this approach, the 3D-printed surgical guide provided excellent stability and was also used for intraoperative registration, avoiding the need for anatomical landmarks. For that purpose, the 3D CAD conventional rigid guide design was imported in Meshmixer software (Autodesk Inc., San Rafael, CA, USA) for design modifications. Several pinholes were added to the surgical guide surface to be used as reference landmarks during patient-to-image registration (**Figure 3A**). A specifically designed socket was also included to attach the dynamic reference frame during navigation or a 3D-printed marker during AR visualization. This socket was positioned on the same jaw as the planned implant but on the opposite side of the arch to avoid interference with the surgical instruments (**Figure 3B**). In general, our rigid guides were designed with a 9- to 12-mm offset between implant head and surgical guide for a 23- or 28-mm drill length. In addition, guiding tubes with a 5.2-mm diameter without metallic sleeves were included to mark the trajectories of the defined implants. Surgical guides were manufactured by the stereolithographic technique with a Formlab Form 2 3D printer and using BioMed Clear V1 biocompatible resin (Formlabs Inc., USA). Finally, surgical guides’ stability was evaluated by fitting them on the cast models and on the patient before surgery. With the advantageous rigid guide in place, holding the registration tracers, we could consider dynamic- or static-guided surgery, augmented reality, or a combination of all of them.

Therefore, VSP surgical translation was individualized in each patient. For example in two of them presenting almost an ideal restored anatomy, or when treating a non-reconstructed jaw, we applied a close transmucosal surgical technique with conventional static-guided surgery concept. In one patient, the mandible was treated with dCAIS and the maxilla with sCAIS. In four patients, we mixed both concepts in the same jaw, starting with a half-guided sCAIS, drilling with the static guide and then placing the implant with dynamic navigation.

**Figure 4:**
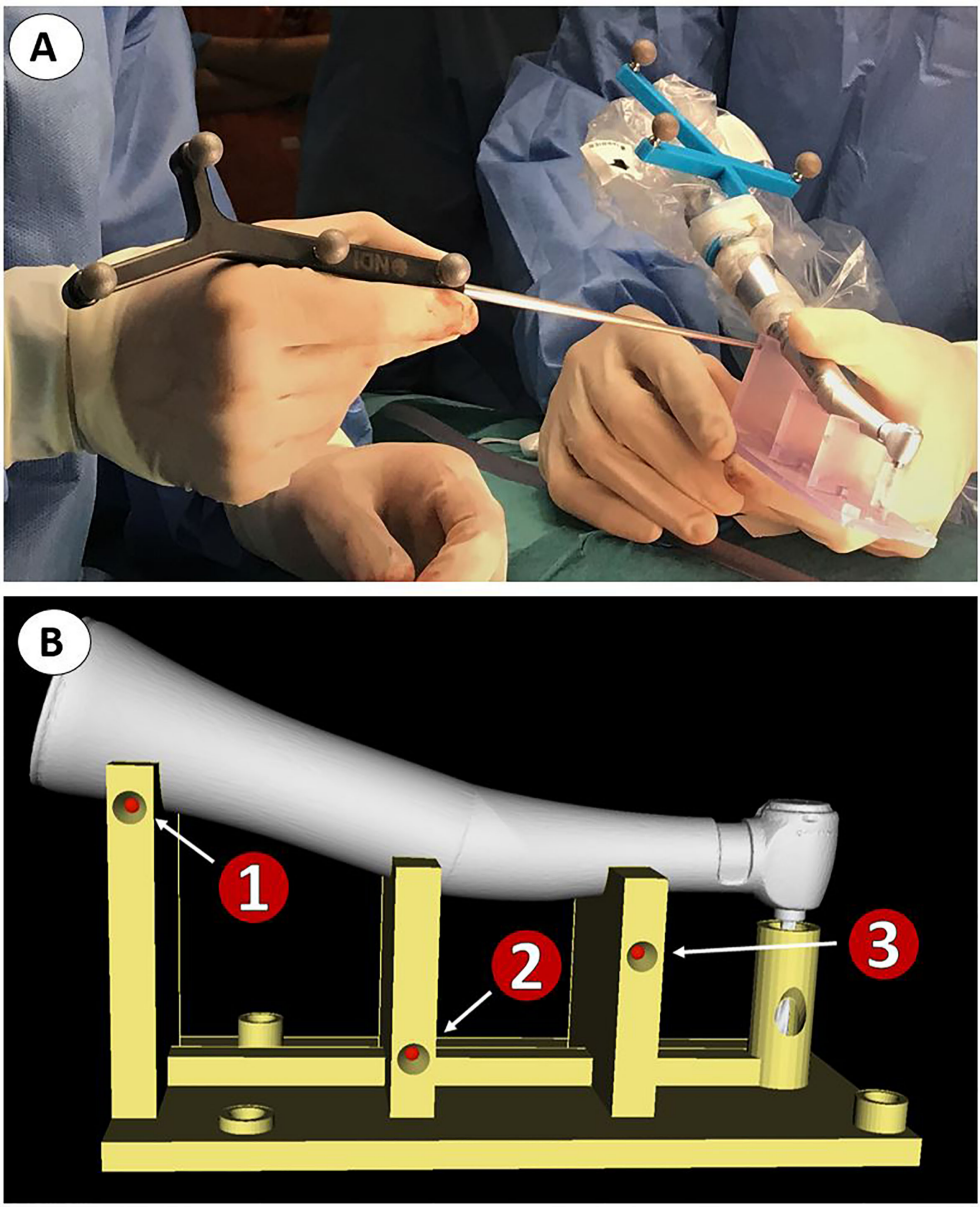
Calibration of handpiece tool prior to dynamic navigation. **(A)** Surgeons recording reference points in the tool calibration platform. **(B)** 3D model of the calibration platform.

A customized in-house software application was developed in 3D Slicer to assist surgeons during dental implant placement. A Polaris Spectra (Northern Digital Inc., Waterloo, ON, Canada) optical tracking system was used for real-time positioning of the surgical instruments with respect to the patient’s anatomy, attaching a dynamic reference frame to the surgical guide. Patient-to-image registration was computed by recording the pinholes included on the surgical guide. This approach ensures an accurate registration when the surgical guide is correctly fixed on the patient. In addition, optical markers were included in the handpiece to track the movements of the tool and guide the drilling trajectory. A calibration step is required to compute the position of the tip of the handpiece with respect to the optical markers. This calibration was performed by fitting the handpiece tool on a specifically designed calibration platform and recording a total of six pinholes located on the platform (**Figure 4**). Head immobilization is no more a requisite for accurate navigation with this workflow due to real-time positioning.

**Figure 5:**
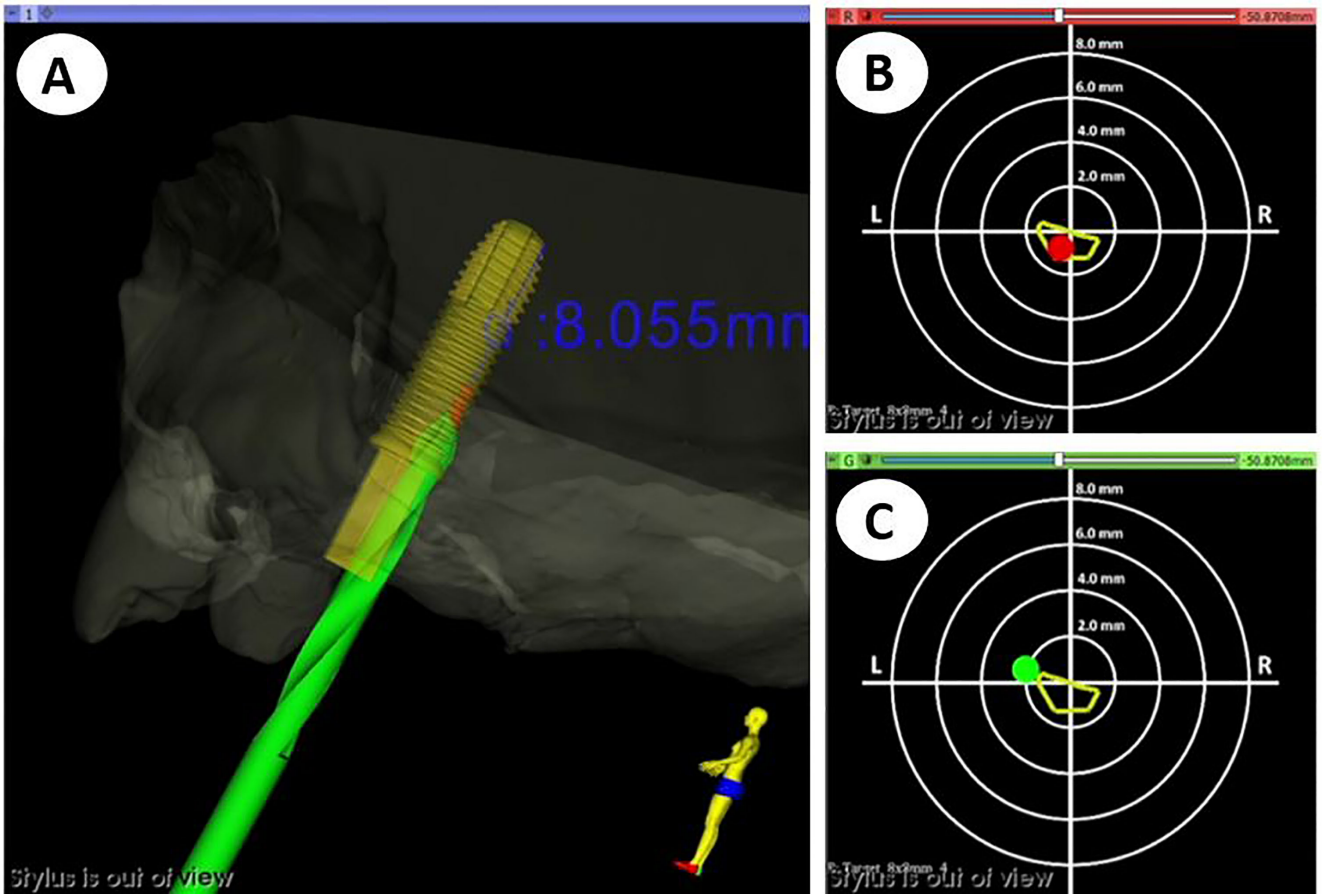
Visualization layout during dynamic navigation. **(A)** 3D view of the handpiece position during drilling with respect to the virtual surgical plan. **(B)** 2D target view to control linear deviation of the handpiece tip at the entry crestal point. **(C)** 2D target view to control angular deviation of the drilling trajectory.

The developed software application displayed the real-time position of the handpiece with respect to the preoperative CT images, anatomical 3D models, and VSP. Optimal drilling trajectory was controlled through constant visual and acoustic feedback to ensure accurate matching with the VSP (**Figure 5A**). The navigation software displayed two target images: one to visualize the linear deviation of the insertion point of the implant (**Figure 5B**), and the other to control the angular deviation of the drilling trajectory (**Figure 5C**, **Video 1**).

**Figure 6:**
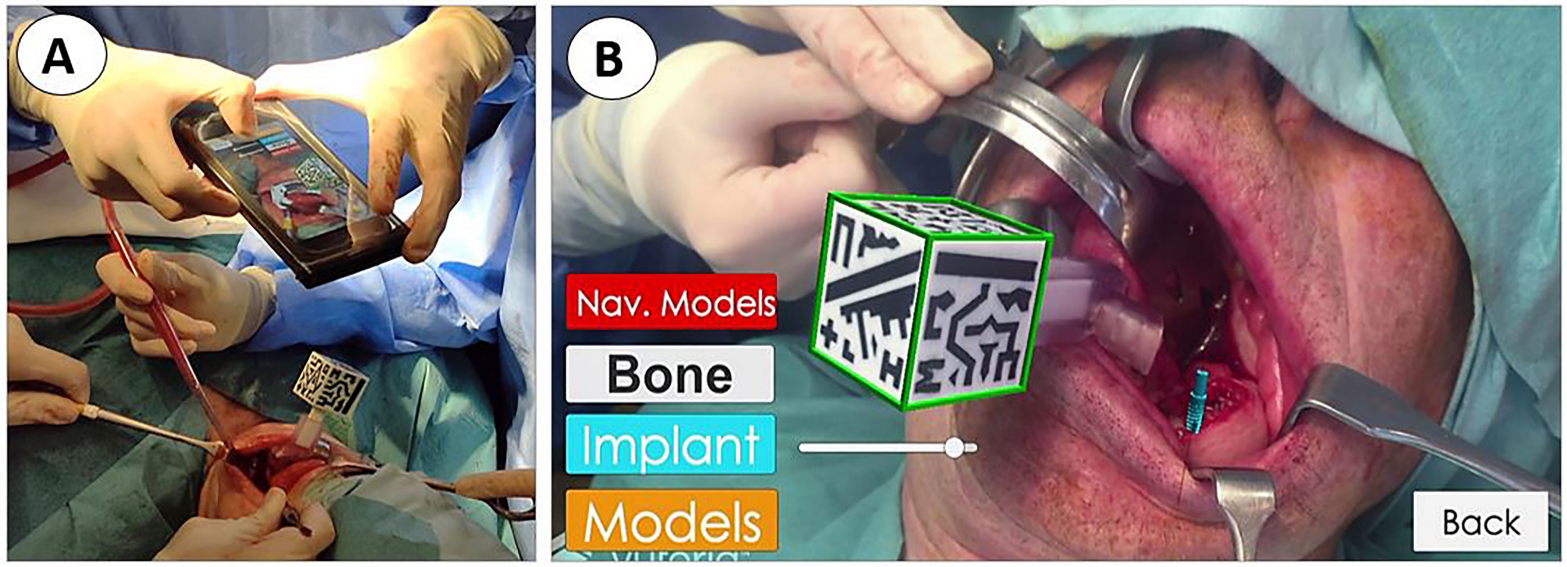
Verification of implant position using augmented reality visualization. **(A)** Surgeon using a smartphone inserted into a sterile cover. **(B)** Augmented reality visualization of the virtual surgical plan overlaid on the patient’s anatomy.

AR visualization was also available for the surgical team and applied in five cases as a tool to verify the final position of the implants. A customized AR smartphone application was developed to project the patients’ virtual models onto the real-world image. This application uses the smartphone camera to detect and track the position of a 3D-printed cubic reference marker for real-time positioning of the virtual models (**Figure 6**). The tracking marker was designed to contain unique black and white patterns on each face (29). This cubic reference was 3D printed in polylactic acid and sterilized with ethylene oxide at low temperature (37°C) before surgery (25).

The developed application was deployed on an iPhone 6 (Apple Inc., Cupertino, CA, USA) and used after the implants were placed on the patient to verify their final position. Once the reference marker was attached to the surgical guide socket, an automatic registration was performed. Then, the smartphone was introduced in a sterilized case (CleanCase, SteriDev Inc., Lansing, MI, USA) and held by one physician. The surgeon pointed with the smartphone camera to the cubic marker, and once it was detected, the virtual models were projected on the smartphone display (**Figure 6A**). The AR device enabled the surgeons to visualize VSP directly on the patient’s anatomy, showing the bone, target implant location, and the optimal drilling trajectories in their expected position (**Figure 6B**), (**Video 2**).

**Figure 7:**
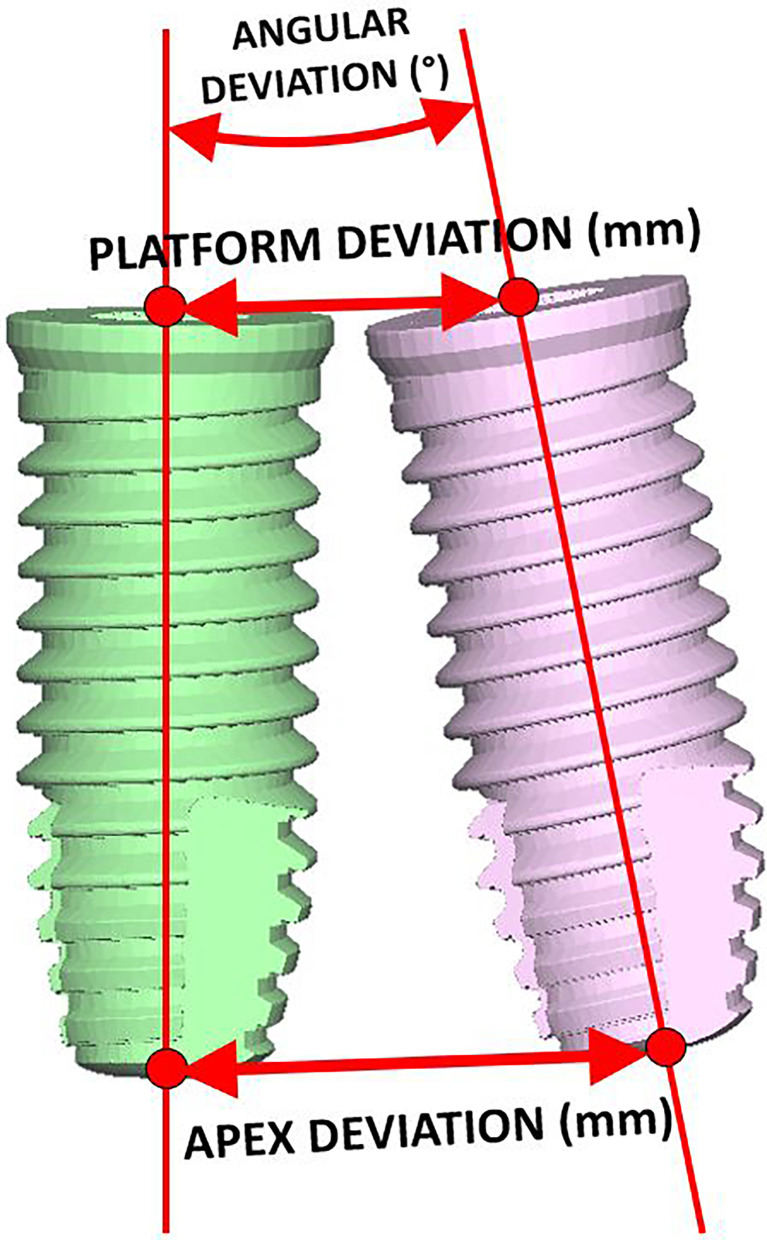
Metrics used to compare the final position of implants with the preoperative virtual surgical plan.

Postoperative CBCT scans were acquired to evaluate surgical outcomes and navigation accuracy. Implants were segmented from the postoperative CT study, comparing their position with the VSP. Accuracy evaluation metrics included (1) linear deviation of the implant entry point (platform or crestal point of insertion) (2), linear deviation of the implant apex or apical endpoint, and (3) absolute angular deviation **(** **Figure 7** **)**. We calculated the mean and standard deviation values of these metrics for each group under study.

## Results

We placed a total of 56 implants, 25 in the upper jaw, and 31 in the lower jaw. 18 implants were inserted into the fibula bone (conventional or double barrel), 6 into the iliac crest, and 32 in the patient remnant bone; 15 implants were placed in irradiated bone (1 fibula and the ALT with vastus lateralis case).

In those eleven patients, we treated 14 jaws, 3 jaws exclusively with sCAIS (closed transmucosal technique, 13 implants), 4 jaws with dCAIS (13 implants), and the other 7 with a combination of both methods (30 implants). In those 11 navigated jaws, we opened a flap for proper bone visualization and soft tissue remodeling. Adequate bone width control is advisable in extremely narrow alveolar bone cases. Vestibular cortical plate fenestration was noticed in three implants, so we extracted the implants and placed them again in different locations. Those three freehand implants were visually oriented and placed in the best-quality bone that was found available intraoperatively without considering the virtual planning. That is why they were withdrawn from the statistical analysis. We also withdrew our third patient (3 implants) from the analysis, since we did not achieve enough stability of the optical markers. Hence, the navigation procedure was not accurate enough, ending the surgery in a conventional non-guided freehand method. Consequently, the implants were placed with an eye-oriented insertion axis and without considering the prosthetically driven surgical planning. Despite the intraoperative complication, the osseointegration was uneventful in the fibula and we ended with the planned fixed screw-retained prosthesis (**Table 1**, patient 5, orthopantomogram with the prosthesis in place). Therefore, we withdrew a total of 6 freehand placed implants from our data analysis

All implants except one achieved a successful osseointegration measured during follow-up by ISQ stability (frequency of resonance), resulting in a 98% osseointegration success rate. This follow-up is, however, too short to extract conclusions.

**Figure 8:**
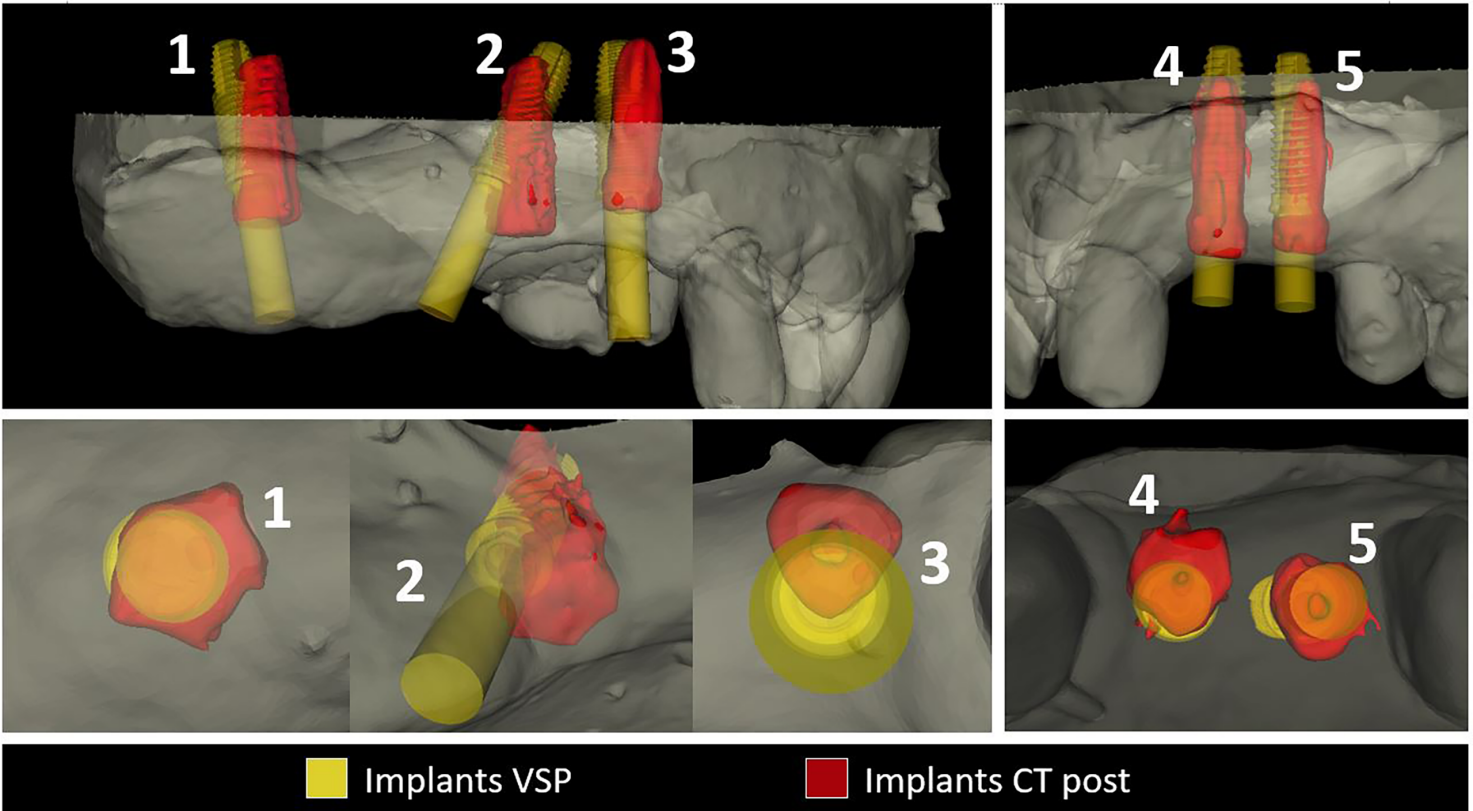
Virtual surgical planning in yellow comparison with final intraoperative position. Excellent accuracy in implants 1,2,4,5, mismatch error in implant 2. Implant 2 while inserting into an extremely narrow alveolar bone developed a vestibular complete fenestration. We intraoperatively corrected the position freehand seeking adequate bone volume. Since this implant is not guided, we withdrew the final result from our study.

**Figure 8** shows an example of the comparison between the virtual surgical planning and the final intraoperative position. Complete data are provided as supplementary material, including patient information, angular deviation, crestal point of insertion, and apical deviation.

The average crestal point insertion error (**Table 2**) was 1.96 mm, with values between 0.35 and 4 mm (standard deviation 0.95 mm). The combination of static and dynamic navigation offers the best accuracy with an average error of 1.52 mm. Static alone has a 1.56-mm average error, and only-dynamic procedures raise the error to 2.7 mm.

Table 2Results of the insertion point deviation in mm.

**Table:** 

PATIENT ID	SUP/INF	IMPLANT ID						AVERAGE	TECHNIQUE
		1	2	3	4	5	6		
ION0001	INFERIOR	3.50	2.47	1.26				2.41	DYNAMIC
ION0002	INFERIOR	2.23	1.99	1.67				1.96	DYNAMIC
ION0003	INFERIOR	6,42*OUT	5,23*OUT	1,89* OUT				OUT	DYNAMIC
ION0004	INFERIOR	3.70	3.11	2.89	4.14			3.46	DYNAMIC
ION005	INFERIOR	2.17	4.37					3.27	STATIC
ION0006	INFERIOR	0.37	0.75	0.35	0.67			0.54	STATIC + DYNAMIC
ION0006	SUPERIOR	0.71	3,11*OUT	1.61	1.04	0.37		0.93	STATIC + DYNAMIC
ION0007	SUPERIOR	3.50	1.78	2.52				2.60	STATIC + DYNAMIC
ION0008	INFERIOR	2.11	3.31	3,99* OUT				2.71	STATIC + DYNAMIC
ION0009	INFERIOR	1.12	3.47	2.93				2.51	STATIC + DYNAMIC
ION0009	SUPERIOR	0.96	0.54	1.28	1.08	2.02	0.80	1.11	STATIC
ION0010	INFERIOR	0.66	1.59	1.76	1.34	0.50	1.10	1.16	STATIC + DYNAMIC
ION0010	SUPERIOR	1.88	1.88	0.81	2.80	0.44	1.20	1.50	STATIC + DYNAMIC
ION0011	SUPERIOR	1.59	1.58	1.89	2,51*OUT	0.43		1.37	STATIC
								1.96	

OUT*. Freehand implant placement, data excluded from analysis

**Table:** 

**GUIDED TECHNIQUE**		**STATIC**	**DYNAMIC**	**STATIC+DYNAMIC**	**TOTAL**				
**AVG**		**1.56**	**2.70**	**1.52**	**1.96**				
**STD**		**1.01**	**0.88**	**0.97**	**0.95**				
**MAX**		**4.37**	**4.14**	**3.50**	**4.00**				
**MIN**		**0.43**	**1.26**	**0.35**	**0.68**				

The endpoint or apical deviation error (**Table 3**) was 2.66 mm, with a range from 0.62 to 7.5 mm (standard deviation 1.33 mm). Considering different guiding techniques, static shows the best accuracy (1.27 mm), followed by the combination with a 2.94-mm error and dynamic alone (3.94 mm).

Table 3Results of the end point deviation in mm.

**Table:** 

PATIENT ID	SUP/INF	IMPLANT ID						AVERAGE
		1	2	3	4	5	6	
ION0001	INFERIOR	5.14	3.74	1.73				3.54
ION0002	INFERIOR	3.22	2.13	3.18				2.84
ION0003	INFERIOR	3,6*OUT	7,45*OUT	4,11*OUT				OUT
ION0004	INFERIOR	3.16	5.67	3.97	7.50			5.08
ION005	INFERIOR	1.35	1.01					1.18
ION0006	INFERIOR	1.30	0.75	0.66	3.03			1.44
ION0006	SUPERIOR	2.41	1,97*OUT	2.41	0.85	1.05		1.68
ION0007	SUPERIOR	7.11	6.75	4.82				6.23
ION0008	INFERIOR	2.75	2.97	2,3*OUT				2.86
ION0009	INFERIOR	2.05	4.54	4.47				3.69
ION0009	SUPERIOR	1.19	0.70	0.90	1.80	2.46	2.30	1.56
ION0010	INFERIOR	0.90	1.95	0.77	1.00	1.07	1.40	1.18
ION0010	SUPERIOR	4.39	3.41	1.86	0.87	2.82	1.44	2.47
ION0011	SUPERIOR	0.62	0.97	1.19	7,91*OUT	0.71		0.87
								2.66

OUT*. Freehand implant placement, data excluded from analysis

**Table:** 

**GUIDED TECHNIQUE**		**STATIC**	**DYNAMIC**	**STATIC+DYNAMIC**	**TOTAL**			
**AVG**		**1.27**	**3.94**	**2.49**	**2.66**			
**STD**		**0.59**	**1.64**	**1.76**	1.33			
**MAX**		**2.46**	**7.50**	**7.11**				
**MIN**		**0.62**	**1.73**	**0.66**				

The angular deviation (**Table 4**) average error was 8.98°, ranging from 1.4° to 30° (standard deviation 5.38°). The combination of static and dynamic and the static alone shows a similar accuracy (8.07 and 8.1 degrees, respectively). Only-dynamic navigation has a 10.5° average error.

Table 4Results of the angular deviation in degrees.

**Table:** 

PATIENT ID	SUP/INF	IMPLANT ID						AVERAGE
		1	2	3	4	5	6	
ION0001	INFERIOR	13.60	11.37	5.75				10.24
ION0002	INFERIOR	9.78	4.19	10.58				8.18
ION0003	INFERIOR	8,18*OUT	10,18*OUT	14,51*OUT				OUT
ION0004	INFERIOR	3.90	9.54	5.76	30.70			12.48
ION005	INFERIOR	16.01	14.21					15.11
ION0006	INFERIOR	5.57	5.57	1.42	13.28			6.46
ION0006	SUPERIOR	10.13	23*OUT	5.14	1.74	3.57		5.15
ION0007	SUPERIOR	15.91	20.90	9.72				15.51
ION0008	INFERIOR	4.79	6.77	15,74*OUT				5.78
ION0009	INFERIOR	7.90	11.95	7.26				9.04
ION0009	SUPERIOR	6.41	2.27	6.12	11.86	6.29	11.77	7.45
ION0010	INFERIOR	7.86	6.25	4.66	1.58	6.08	2.71	4.86
ION0010	SUPERIOR	12.59	13.11	4.72	12.44	12.01	10.44	10.89
ION0011	SUPERIOR	6.55	6.07	5.50	33,07*OUT	4.11		5.56
								8.98

OUT*. Freehand implant placement, data excluded from analysis

**Table:** 

**GUIDED TECHNIQUE**		**STATIC**	**DYNAMIC**	**STATIC+DYNAMIC**	**TOTAL**			
**AVG**		**8.10**	**10.52**	**8.07**	**8.90**			
**STD**		**4.09**	**7.40**	**4.63**	5.38			
**MAX**		**16.01**	**30.70**	**20.90**				
**MIN**		**2.27**	**3.90**	**1.42**				

**Table 5** summarizes the results for angular deviation and crestal and apical deviation. Each variable is subdivided into three groups for descriptive data analysis: static-guided surgery or dynamic navigation alone, and the combination of both.

**Table 5:** Average, standard deviation and maximum and minimum values of the results for angular deviation, insertion point deviation, and end point deviation.

		AVG	STD	MAX	MIN
**ANGULAR DEVIATION (degrees) 8.98°**	**STATIC**	8.10	4.09	16.01	2.27
	**DYNAMIC**	10.52	7.40	30.70	3.90
	**STATIC+DYNAMIC**	8.07	4.63	20.90	1.42
**INSERTION POINT (mm) 1.96**	**STATIC**	1.56	1.01	4.37	0.43
	**DYNAMIC**	2.70	0.88	4.14	1.26
	**STATIC+DYNAMIC**	1.52	0.97	3.50	0.35
**END POINT DEVIATION (mm) 2.66**	**STATIC**	1.27	0.59	2.46	0.62
	**DYNAMIC**	3.94	1.64	7.50	1.73
	**STATIC+DYNAMIC**	2.49	1.76	7.11	0.66

A fixed screw-retained prosthesis has already been placed in five patients as planned, while the last six patients are waiting to complete the osseointegration period. We duplicate the period of osseointegration in irradiated bone.

At the end of the surgical procedure, we were able to display planned implant placement intraoperatively with the AR app on our mobile phone in five patients. The final platform and the insertion point deviation were visually verified in 30 implants. The match between virtual planning and final surgical results was observed and recorded.

## Discussion

Virtual computerized implant surgery has opened a new horizon in the management of complex cases when the anatomy of the jaw bones has been altered due to trauma or pathology (30). In free flap oncologic reconstructed patients, there are several reports regarding guided static surgery-based implant placement (8, 16, 17) but only a few about dynamic navigation and none reporting a combination of both or augmented reality guidance.

There is a gap between surgical planning and interventional procedures in oral and maxillofacial surgery. Medical CAD/CAM technologies ensure a precise virtual plan. However, their translation to the operating room cannot be guaranteed due to the lack of accurate surgical guidance and anatomical visualization during the procedure (31).

The deviation range of an ideal surgical navigation system to meet the clinical requirements should be between 0.5 and 1.5 mm. The more complicated the surgical procedure is, the greater the error should be expected (32–34). The literature seems to indicate that one has to accept a dynamic unavoidable inaccuracy of 2.0 mm in any guided surgery procedure (35). The accuracy of the registration process between the virtual image and the surgical site has the most significant impact on the precision of the navigation system since subsequent tasks depend on that step. Several registration methods have been proposed in craniofacial surgery: bone implants (plates and screws), occlusal splint fitted to the teeth, anatomical landmarks, and laser surface scanning. Occlusal splints provide a non-invasive, highly accurate registration method that is steadily fitted to stable bony landmark-dental cusps (33).

The position of the dynamic reference frame in relation to the surgical site requires consideration. The aim is to position the reference frame as close as possible to the surgical field to maximize navigation accuracy, but considering that it should not limit the surgeon’s maneuvers during the intervention. Jiang et al. (33) found that the closer (further) the distance from the reference frame, the smaller (larger) the positional deviation, showing a similar trend for the angular error. They concluded that an occlusal splint might be sufficient for the navigation of maxillary and mandible surgery.

Nevertheless, our first four cases were splintless dynamic-guided surgeries (one iliac crest, one fibula double barrel, and two conventional fibula flaps). We applied the tooth-supported silicone jig dCAIS concept for patient registration, which was the most inaccurate method (linear crestal insertion error 2.7 mm, apical deviation 3.94 mm, and 10**°** of angular mismatch). These values are similar to those reported in the literature for conventional freehand non-guided placement in simple non-oncological cases. Vercruyssen et al. (35) revealed a crestal error of 2.7 mm, an apical error of 2.9 mm, and an angular deviation of 9.9°. For the same features, Block et al. (36) reported 1.67 mm, 2.51 mm, and 7.69**°,** respectively. In our study, the tooth-supported silicone jig provided an adequate registration. However, it resulted unstable during surgery. Consequently, the cases with large anatomical distortion (fibula cases patients 3 and 4 with a basal fibula bone placement and a thick skin paddle) showed the lowest accuracy. Moreover, we had to stop the navigation procedure in the third patient and continue with a conventional freehand method. Despite the mismatch, osseointegration was uneventful and the initial group of four patients ended with adequate screw-retained implant-supported prosthesis.

To overcome this stability problem, we introduced the sCAIS in our study, based on a 3D-printed teeth-supported surgical guide that not only stabilized the fiducial markers for accurate registration and navigation but also demonstrated to be a good alternative for implant placement if needed.

Static CAIS systems are limited due to undesirable cooling methods, restricted direct visual contact with the working surgical site (blind technique), and the impossibility of modifying the planned position intraoperatively. Placement and stability of the guide during the surgical procedure are critical to achieving precision. Some sources of error, in oncological reconstructed patients, could be the following: limited mouth opening in patients, nature of the guide support, tooth availability, tooth position or mobility, template fabrication process or flap approach, in particular concerning posterior surgical sites, and the need of long drills in restricted mouth opening patients (37). Previous studies have shown that tooth-supported guides provide better results than mucosa or bone-supported guides. Implants in distal extension gaps resulted in more significant deviation when compared to implants placed in posterior areas with adjacent bilateral teeth support due to possible intraoperative guide movement, tilting, and bending, particularly in long cantilever lengths. Although the mismatch between the planned and final achieved positions can be measured, no information on the source of inaccuracy can be assessed (38). Most of our patients belonged to the group of long posterior extension gaps prone to bending and tilting of the static guide. Therefore, we expected difficulties with static guide accuracy in most of our cases. In addition, sCAIS needs specific surgical drills and instruments.

We restricted the use of static surgery to three patients, 13 implants, carefully choosing the jaws with minimal anatomical distortion, ideal tooth support for splint stabilization, non-restricted mouth opening, and almost average mucosal or soft tissue flap thickness.

sCAIS accuracy results in a healthy population (partially edentulous non-oncological cases) were analyzed by two systematic reviews, Tahmaseb et al. (39) and Jung et al. (40). They reported an entry point error ranging from 1.04 to 1.45 mm, apex mean error between 1.38 and 2.99 mm, and angular error around 4°. Therefore, it is considered a highly accurate method, but many studies are biased reporting single-unit cases with ideal tooth support on both sides of the edentolous space. Our results after a careful selection of patients are respectively 1.56 mm, 1.27 mm, and 8°, similar to non-oncological series. sCAIS usefulness is limited in oncologic patients due to the aforementioned restrictions. It should be considered in irradiated patients when feasible because it allows a close surgical approach. Flapless surgery is a less invasive and traumatic procedure, avoiding raising a flap and detaching undamaged soft and hard tissue from its vascularization, which could be crucial in irradiated patients (8).

On the other hand, open dynamic image-guided navigation techniques enable real-time surgical tool tracking and visualization with respect to surrounding anatomical structures, allowing the surgeon to accurately place the implant on the position defined during preoperative virtual planning. The surgeon’s perception of the drilling sequence and implant placement is not affected by a splint. There is no need for a specific set of drills or instruments and can be used in almost all patients, even in cases with limited mouth opening. Kalaivani et al. (13) stated that the major value of the dynamic design is the ability to adjust the planned implant positioning intraoperatively.

dCAIS tracking depends on the registration procedure. Errors in that step could be detected and corrected with continuous recalibration paying attention to reference fixation and position stability. Nevertheless, there are some limitations to evaluate since the registration process is technically sensitive and requires time. In addition, the surgeons need a steep learning curve and the cost of the equipment is high (41). Sun et al. (42) reported that the learning curve plateau is not reached until the surgeon has placed at least 15 dental implants with these systems. Our group created a workflow based on open software, avoiding extra costs, and included laboratory practice with biomodels to reduce the learning curve.

dCAIS in healthy non-oncological case results are excellent, and similar to the sCAIS, Yimarj et al. (19) reported a 1.24-mm crestal insertion error, 1.58-mm apical mismatch, and 3.78° angular deviation. In another systematic review and meta-analysis, Wei et al. (17) reported that the average global platform deviation, global apical deviation, and angular deviation were 1.02 mm, 1.33 mm, and 3.59°, respectively. Our subgroup of five patients, 30 implants, placed by means of a splint-based registration dynamic navigation technique yielded values of 1.52 mm, 2.49 mm, and 8°, respectively, slightly higher than in non-oncological patients.

When comparing sCAIS with dCAIS, Kaewsiri et al. (21), Mischkowski et al. (22), and Block et al. (36) concluded that dynamic navigation provided higher accuracy than any static guide system but without statistical significance. On the other hand, Jorba-García et al. (43) considered that not all commercial dynamic systems are suitable for treating difficult fully edentulous cases, suggesting the use of static systems as the first-line option in guided implant surgery.

Our workflow shares the best capabilities from both methods. Assuming that the surgical template is difficult to use alone in anatomically altered reconstructed oncologic patients and that dynamic navigation information allows intraoperative real-time modifications, we combine both techniques. The rigid tooth-supported acrylic 3D-printed splint provides a stable platform for patient registration and optical marker display. It also holds all the information for guided surgery and the cylinders for static drilling. In our last five patients, we placed the tip of the drill inside the cylinder using dynamic navigation to find the virtually planned crestal insertion point and the best handpiece axis orientation before starting to drill. The main difference with a conventional static drilling technique is that we have a certain degree of freedom within the guiding 5.2-mm-diameter tube, to slightly change the drill insertion point and axis as suggested by the intraoperative dynamic navigation display in order to match the virtual surgical plan. With this combined approach, our results in the last three oncologic patients resemble those achieved in healthy non-oncological patients.

**Figure 9:**
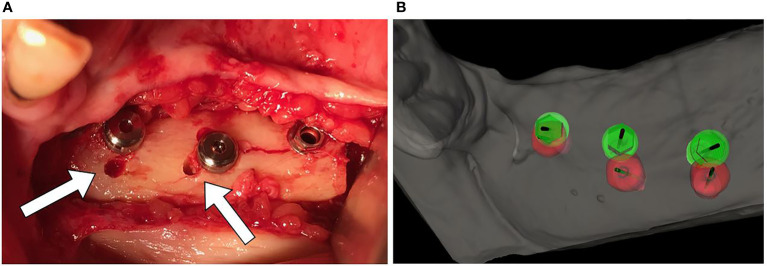
**(A)** Mismatch between the initial 2-mm drilled holes suggested by the static splint (see white arrows) and the final intraoperative implant position achieved with dynamic navigation guidance. **(B)** Static splint-based dynamic navigation accuracy is higher than static navigation alone in this double-barrel fibula case. Postoperative implant position analysis, the achieved position in red, the virtual surgical planned position in green.

In many implants, we noticed an intraoperative mismatch in the crestal insertion point between the static surgical guide and the dynamic navigation. We give more credit to the position provided by the navigation technique, and the postoperative implant position analysis reveals that dynamic navigation based on a stable splint offers superior accuracy to translate the virtual surgical planning into the operating room (**Figure 9**).

Considering the image-guided surgery learning curve, it is difficult to draw conclusions because all our oncologic patients are different and each case should be individualized while planning (**Table 6**). Our series showed a progressive increase in accuracy and indeed a learning curve despite preclinical laboratory training. The combination of a modified navigation static 3D-printed splint and infrared optical navigation yielded the best results in all the measured variables.

**Table 6:** Learning curve, from the initial jig dynamic navigation to combined, static-dynamic-AR, guided surgery.

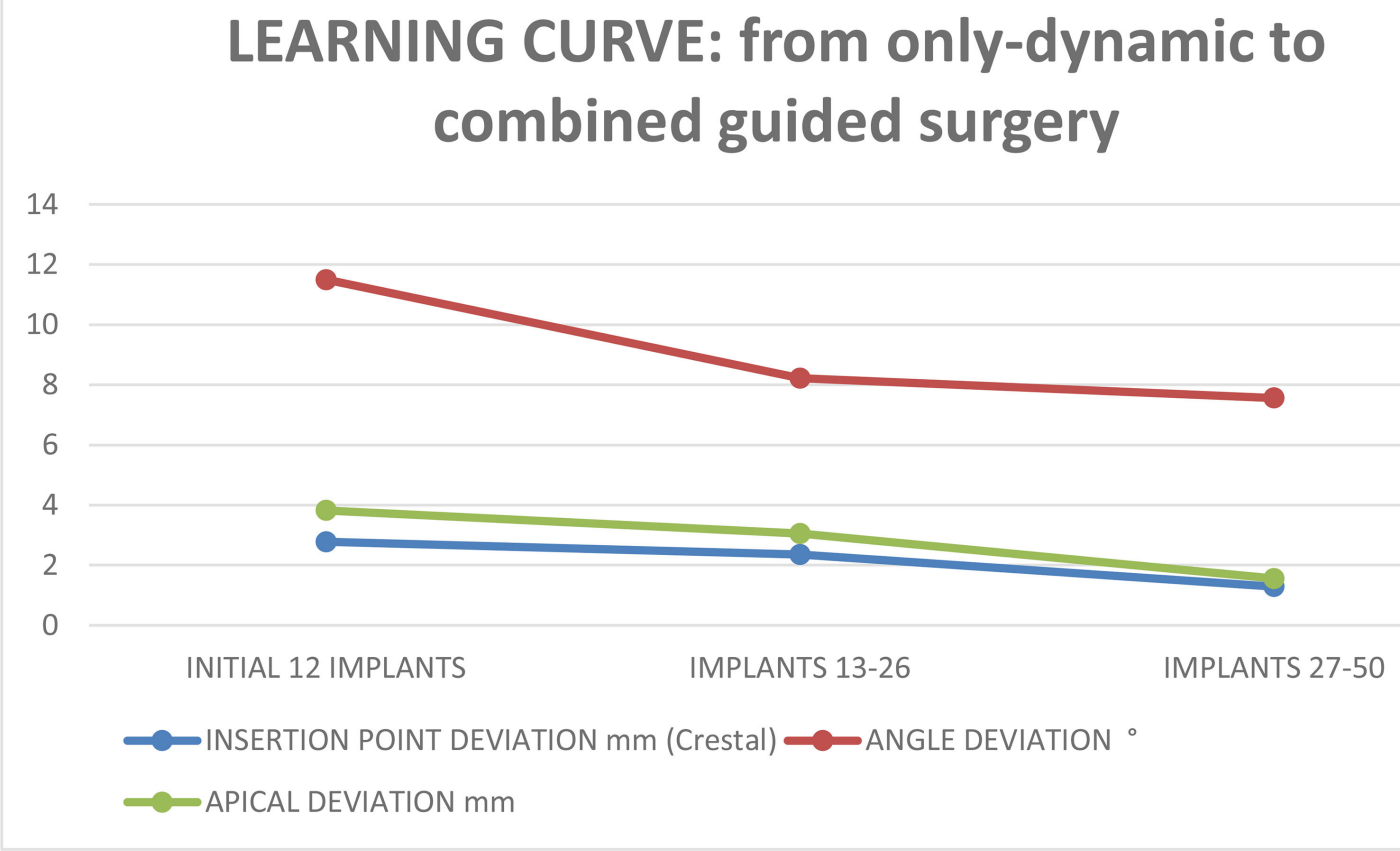

Clinical studies about augmented reality in maxillofacial implantology are scarce with very few publications investigating this technology. Pellegrino et al. (24) presented two cases where they evaluated the feasibility of adopting Hololens glasses-guided AR as a means of facilitating the use of dynamic navigation. Pellegrino placed just one implant on each patient and found a 0.5–0.53-mm deviation at the entry point, a 0.46–0.48-mm deviation at the apical portion, and an angular deviation between 2.19° and 3.05°. They concluded that AR overcomes one of the disadvantages of the dynamic guided system: the need to simultaneously pay attention to the patient and the output from the navigation system display. However, when a 3D virtual layer is displayed and laid over the real environment, there is often a discrepancy between the real and virtual images due to an overlay or positional error. In their opinion, the main limits of AR are the inconvenient virtual window positioning and orientation together with the working distance of the glasses, which could force the surgeon to operate in an uncomfortable position, and the lack of depth perception. In addition, when comparing visual perception of depth on a 3D image with a 2D screen projection, stereo and motion parallax are two missing essential cues (34). These reasons explain why AR is still a field of *in vitro* investigation in maxillofacial surgery with few clinical reports.

We developed a smartphone-based AR navigation system combining an AR application with a 3D-printed reference marker. One of the main advantages of our approach is that there is no need to wear special heavy and bulky head-mounted displays or divert surgeons’ eyes from the surgical site, since the smartphone display could overlap the operating area. Another advantage is the automatic patient-to-image registration method, thanks to the reference marker placed into the surgical guide socket. Wang et al. (31) presented an interesting alternative with a stereo camera marker-less image registration method where the only requirement to generate correct AR scenes is to expose the patient’s teeth to the camera.

The smartphone was easy to use in the surgical field thanks to the sterilized case. The proposed smartphone app could display the bone, virtual implant planning, and plaster model holding the fixed screw-retained planned prosthesis in the real position on top of the patient. Regarding the implants, it was easy to superimpose, with different degrees of translucency, the planned virtual implant over the final intraoperative position. We applied this in five patients as a final validation method of the guided surgery with favorable results. However, depth perception is still a limitation in AR. Therefore, we believe that using AR technology for surgical guidance is still challenging, and further research and laboratory practice are needed to overcome barriers.

Increasing economic costs and operating time should be mentioned as potential limitations. There are no extra costs in our proposal since planning and splint design is based on free software. We establish a negligible 20 Euros cost for splint exportation and printing. The software allows subsequent splint modifications and impression for a bimaxillary splint without new extra fees for each patient. Regarding surgical time, we calculate that 15 min of extra time are needed for subsequent iterative registration procedures during a three-implant-guided placement.

Despite that, non-oncological implant placement is gradually becoming a navigated surgery. Dental implantologists are increasingly applying this method due to the undoubted advantages. As stated by Michael S. Block in 2017: “Clinicians placing implants should consider routinely using dynamic navigation on daily basis to improve their accuracy and not just for special cases” (41). In our experience, any guided method, even the most inaccurate, yielded at least similar results when compared to the freehand technique.

## Conclusion

Oncologic patients reconstructed with free flaps represent a challenge for implant treatment. Image-guided implant surgery should be used with caution, since previous results from these techniques cannot be directly translated from normal healthy non-oncologic cases. The static surgery concept could be easily used in non-reconstructed jaws or when the anatomical distortion is minimal and good tooth support ensures surgical guide stability.

Combining a modified static-guided surgery tooth-supported 3D-printed resin guide with dynamic navigation (modified for accurate registration and optical markers display) in oncologic patients could achieve equivalent results to those obtained with guided methods in healthy non-reconstructed patients.

Furthermore, intraoperative flexibility allowing alignment and orientation modifications during implant placement is a significant advantage of VSP and dynamic guided surgery. Augmented reality is a valuable tool for intraoperative verification but needs further research to be considered an alternative guided method for implant surgery.

Computer-aided implant surgery based on dynamic navigation and 3D-printed surgical modified guides is an accurate and valuable technique for prosthetically driven implant placement in free flap oncologic reconstructed patients.

## Data Availability Statement

The raw data supporting the conclusions of this article will be made available by the authors, without undue reservation.

## Ethics Statement

The studies involving human participants were reviewed and approved by Comité de Ética de la Investigación con Medicamentos Hospital General Universitario Gregorio Marañón. The patients/participants provided their written informed consent to participate in this study.

## Author Contributions

SO, DG-M, and JP conceived and designed the study. SO, DG-M, JP, and AG wrote the main manuscript. SO defined the virtual surgical plan and designed the surgical guides and dental rehabilitations for all patients. DG-M developed the navigation software, performed the data analysis, and edited the supplementary videos, DG-M and AG manufactured the surgical guides and navigation tools. DG-M and AG prepared the figures and tables. SO, MT, CN-C, IN-C, and JS were the surgeons at the ablative, reconstructive free flap procedures and at implant placement. They also made the implant supported fixed dental rehabilitation at the outpatient clinic. RM-M designed the workflow for augmented reality visualization, designed the APP for the mobile phone, and intraoperatively collected the data and images. All authors reviewed the manuscript. All authors contributed to the article and approved the submitted version.

## Funding

This work was supported by grant PI18/01625 (Ministerio de Ciencia e Innovación, Instituto de Salud Carlos III and European Regional Development Fund “Una manera de hacer Europa”). This study was also supported by Ticare® implants (Mozo-Grau, Valladolid, Spain). The funder was not involved in the study design, collection, analysis, interpretation of data, the writing of this article or the decision to submit it for publication.

## Conflict of Interest

The authors declare that the research was conducted in the absence of any commercial or financial relationships that could be construed as a potential conflict of interest.

## Publisher’s Note

All claims expressed in this article are solely those of the authors and do not necessarily represent those of their affiliated organizations, or those of the publisher, the editors and the reviewers. Any product that may be evaluated in this article, or claim that may be made by its manufacturer, is not guaranteed or endorsed by the publisher.
