# Supplementary material for: Computer-Assisted Dental Implant Placement Following Free Flap Reconstruction: Virtual Planning, CAD/CAM Templates, Dynamic Navigation and Augmented Reality
Source: Front Oncol. 2022 Jan 28;11:754943. doi: 10.3389/fonc.2021.754943 (PMC8833256; doi:10.3389/fonc.2021.754943)
Supplement: Supplementary file 1 [file DataSheet_1.pdf]

ION001 - INF

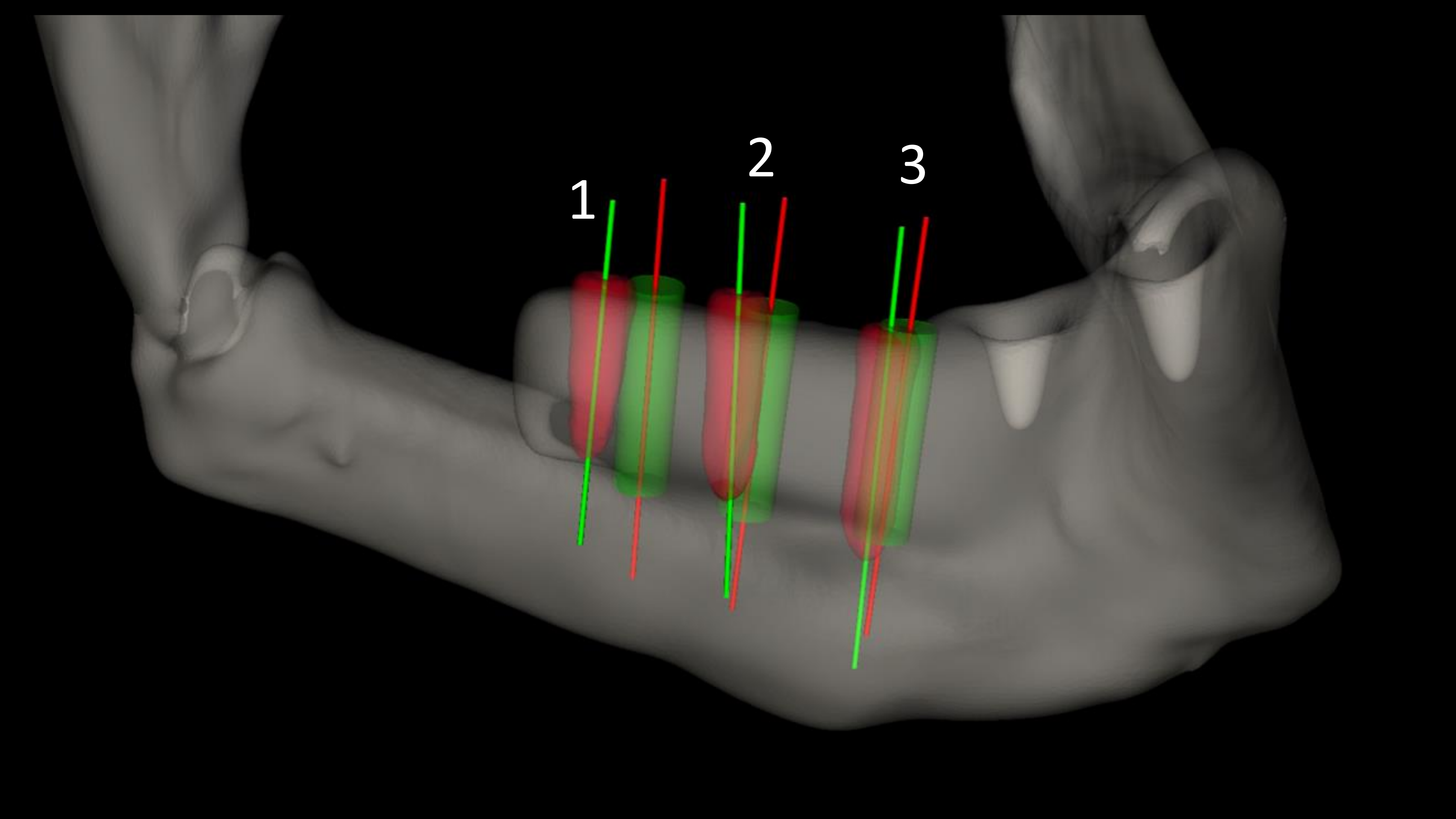

1
2
3

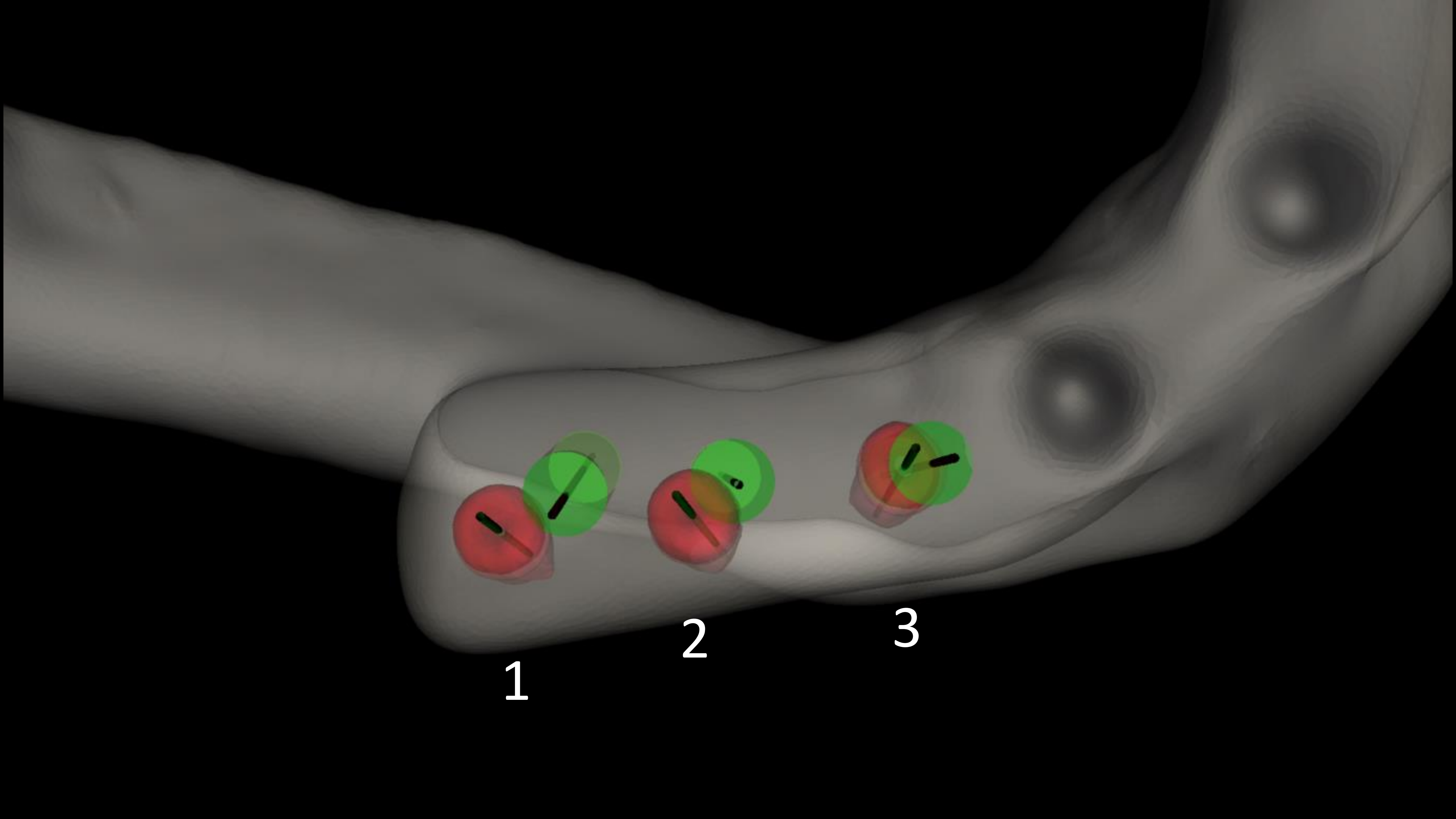
1
2
3

ION002 - INF

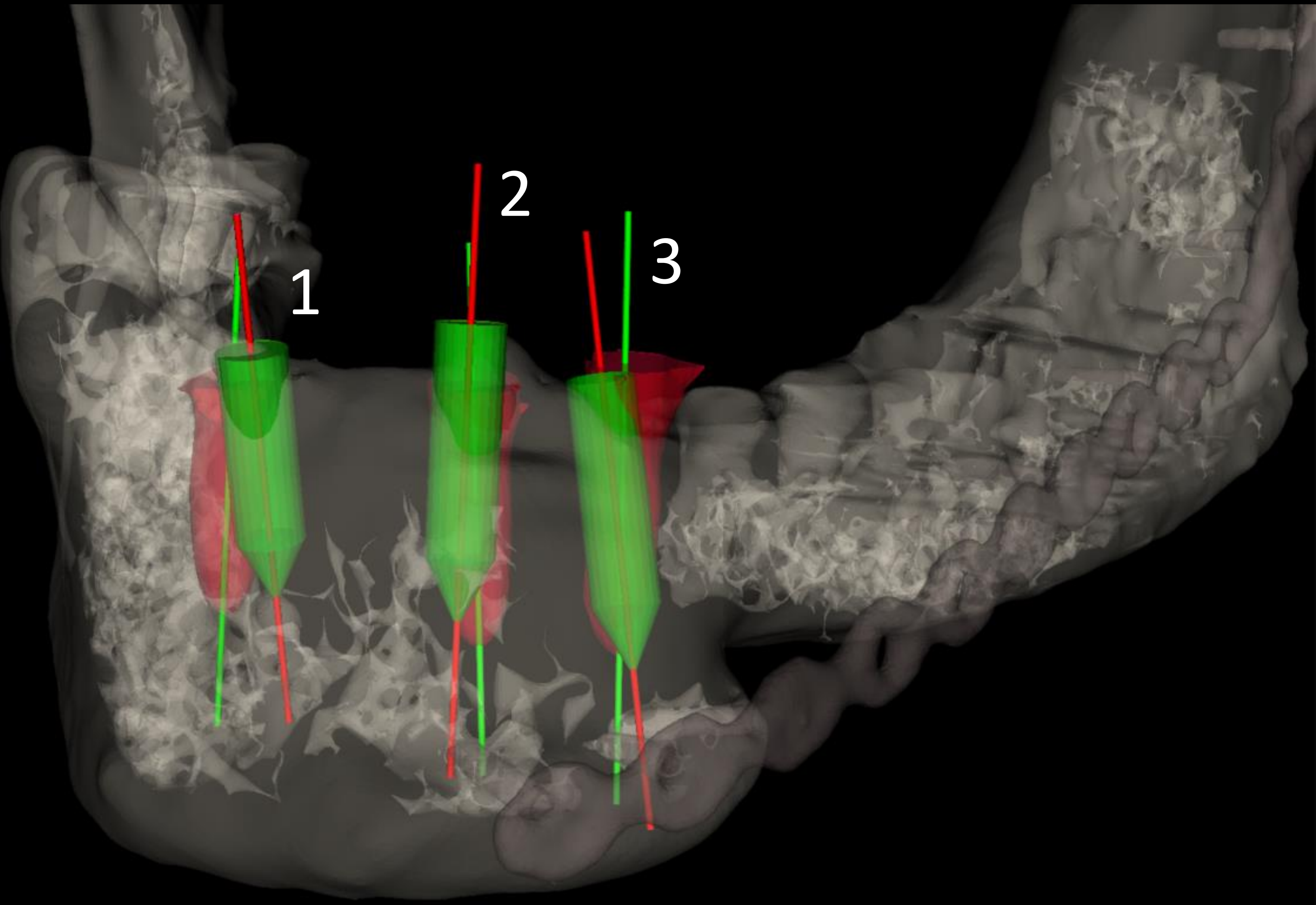
1
2
3

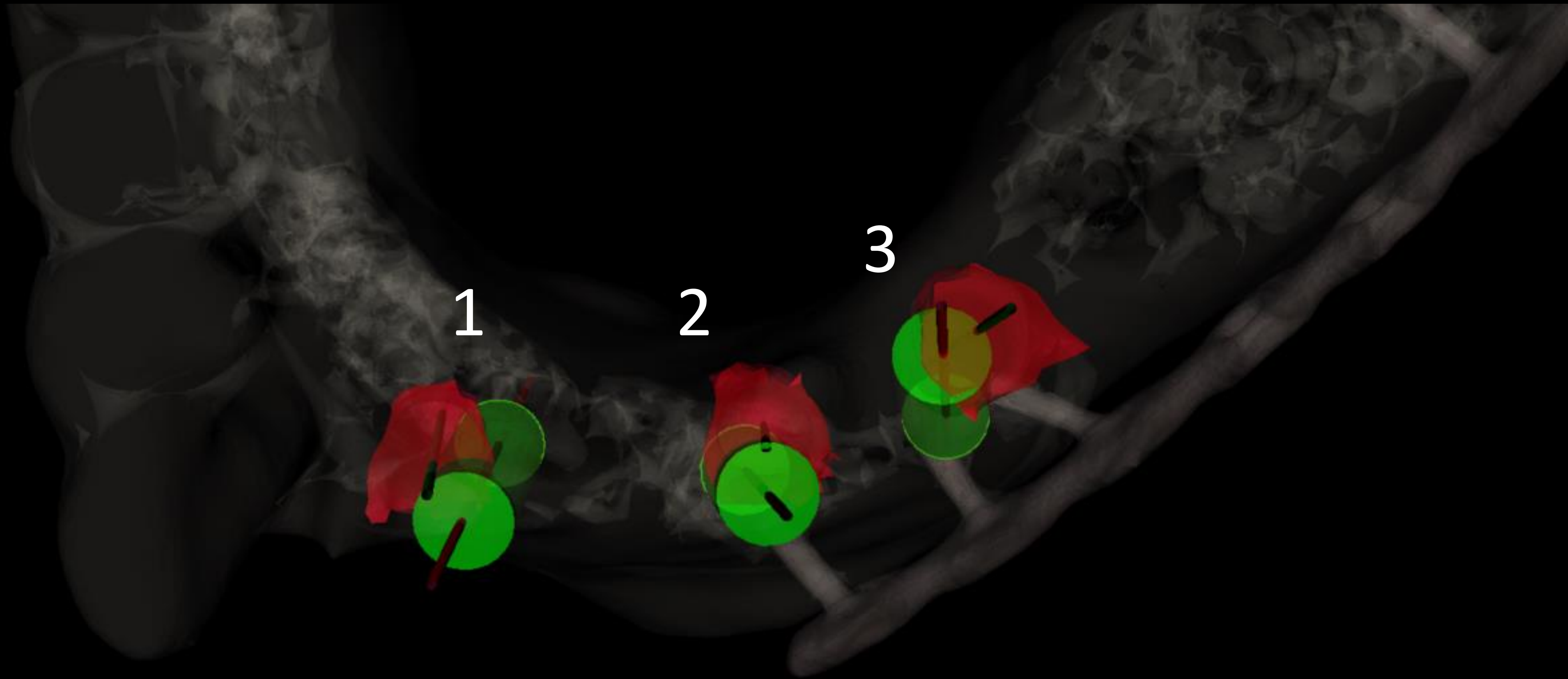
1
2
3

ION003 - INF

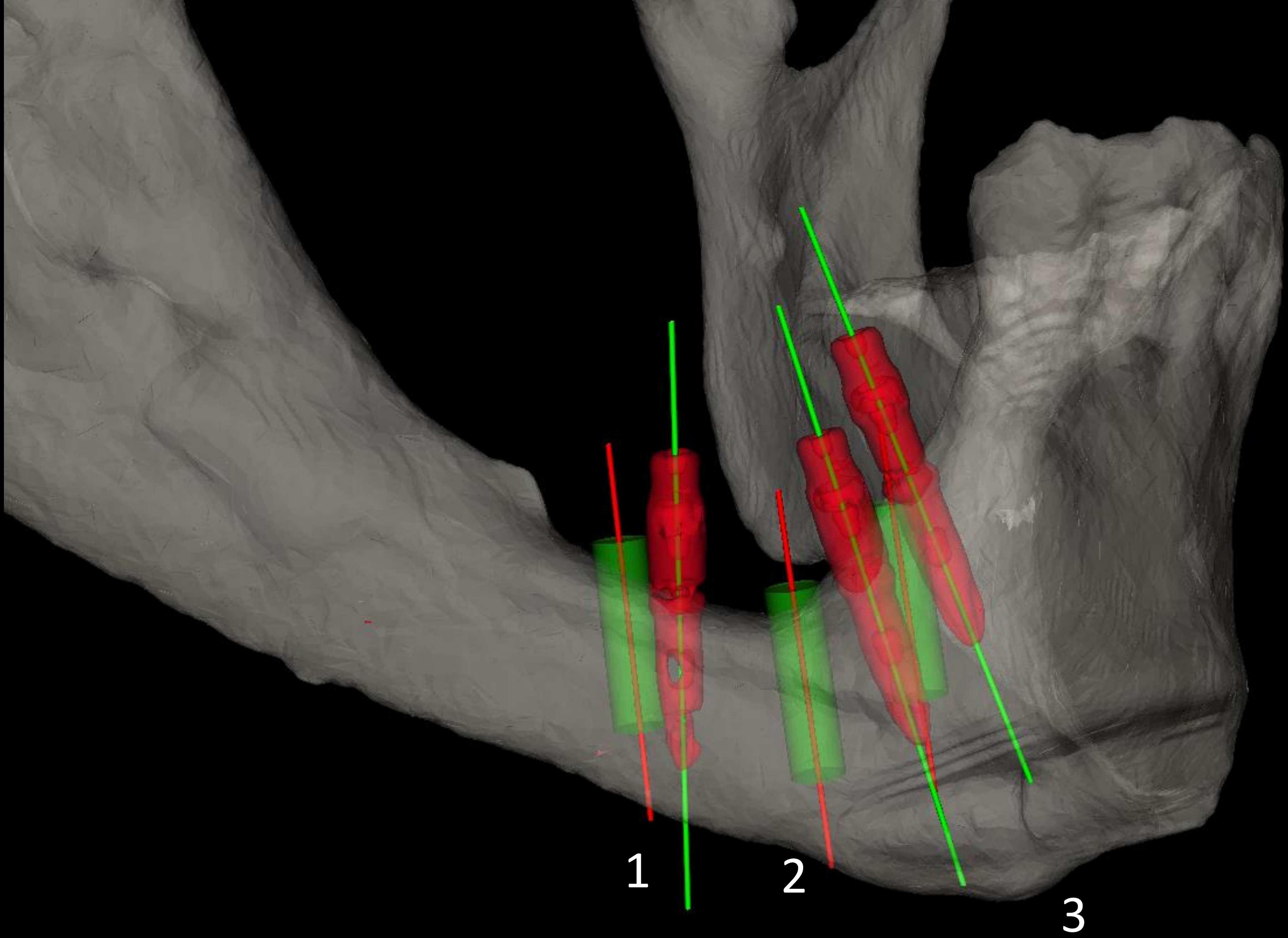
1
2
3

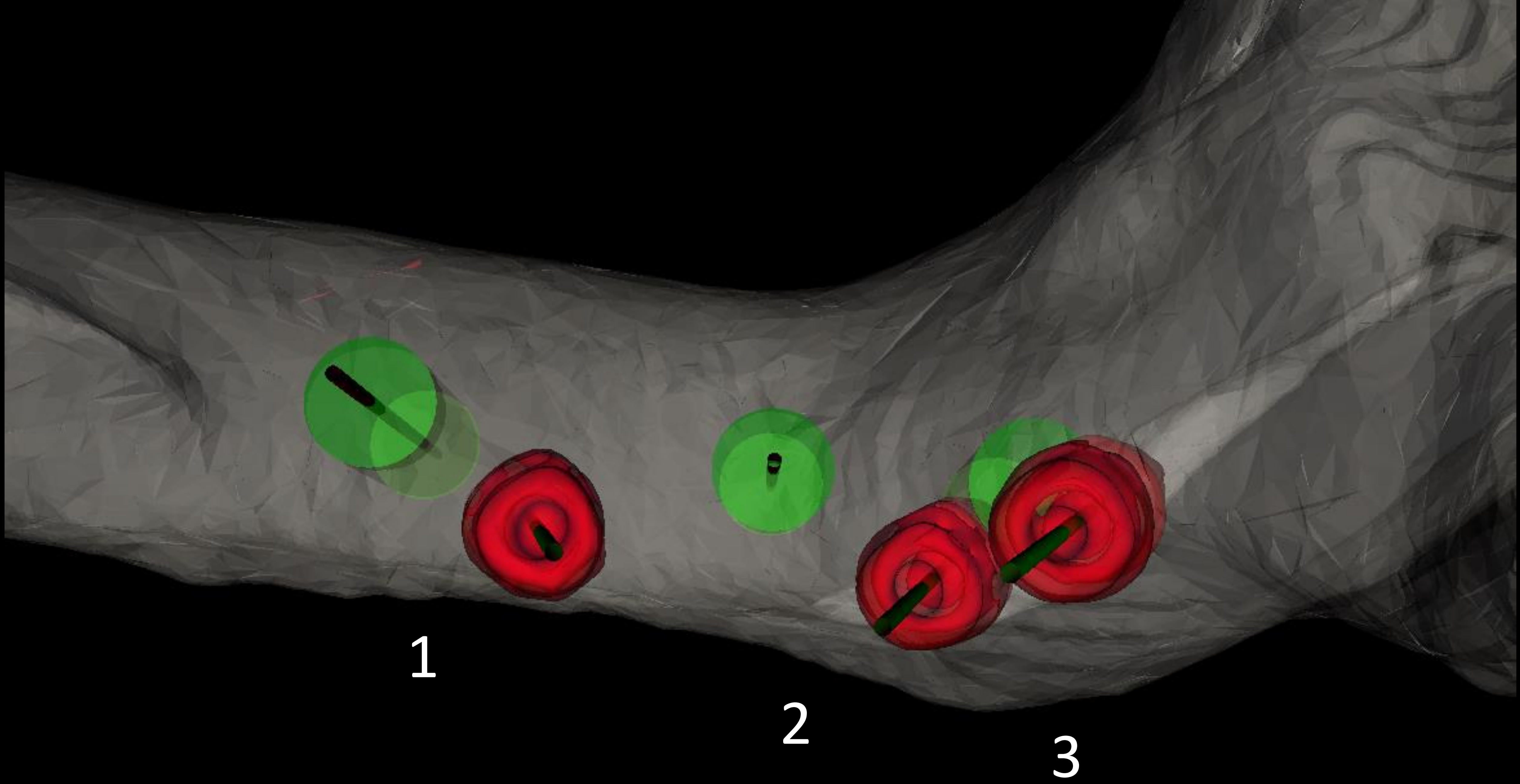
1
2
3

ION004 - INF

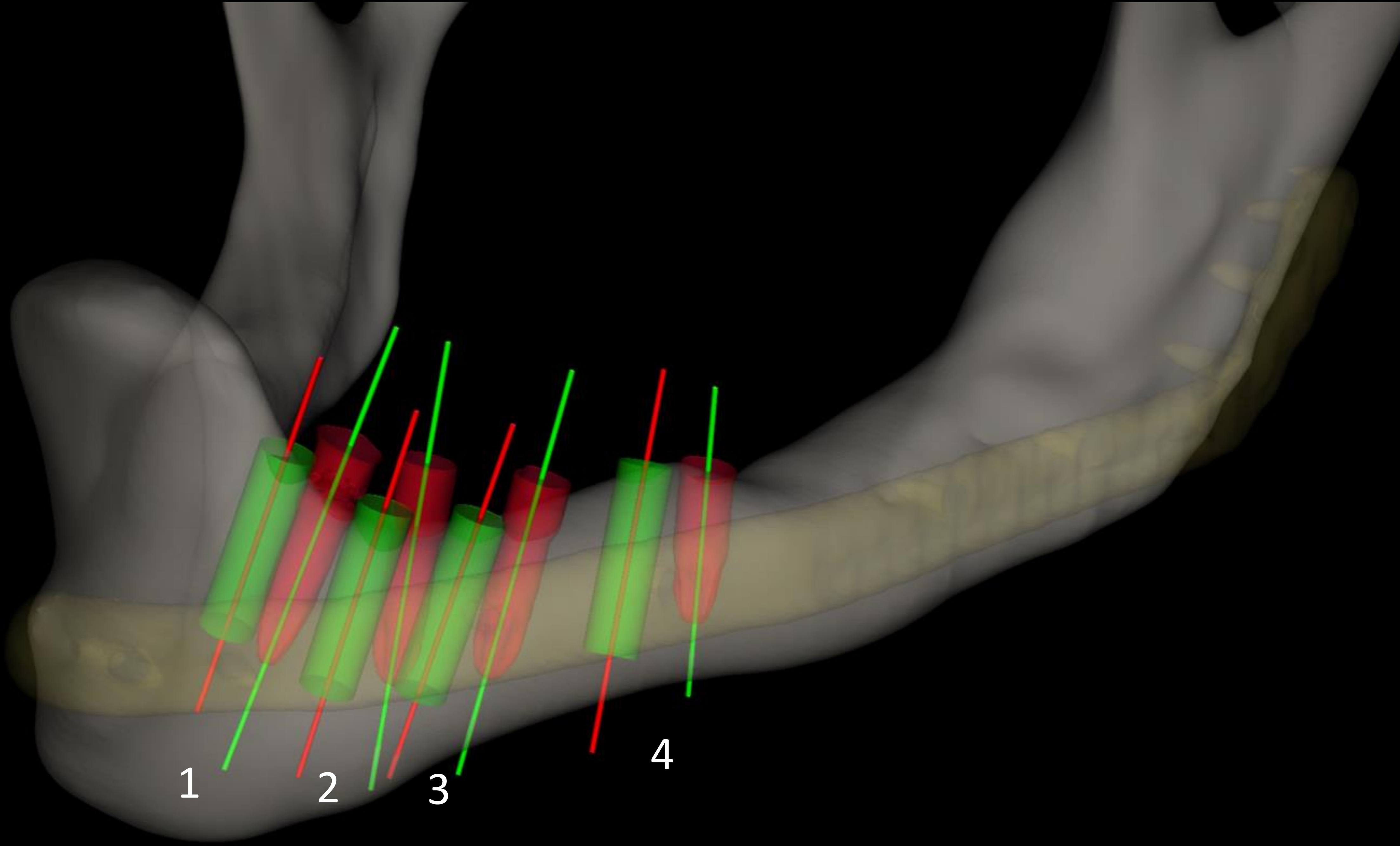
1
2
3
4

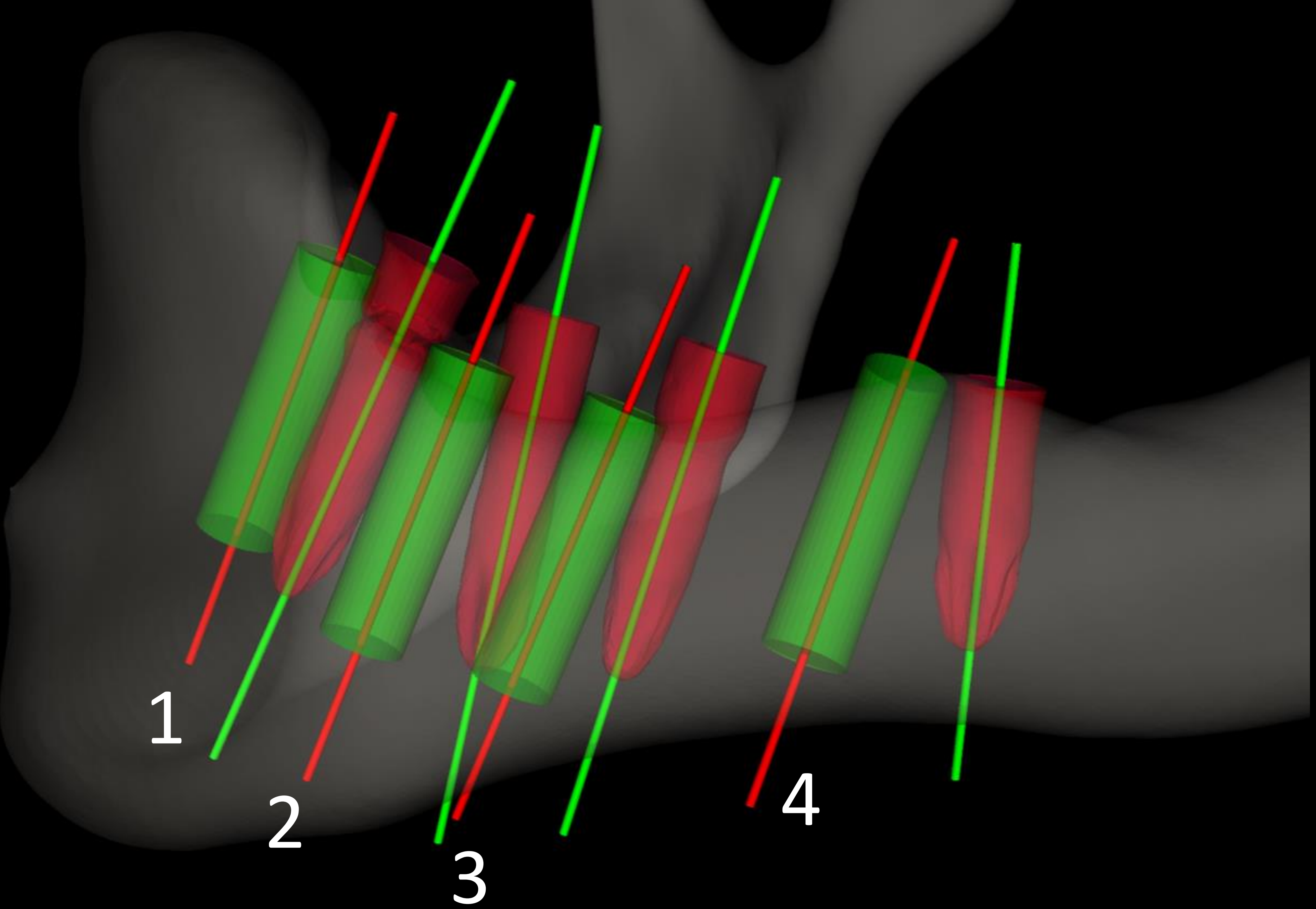
1
2
3
4

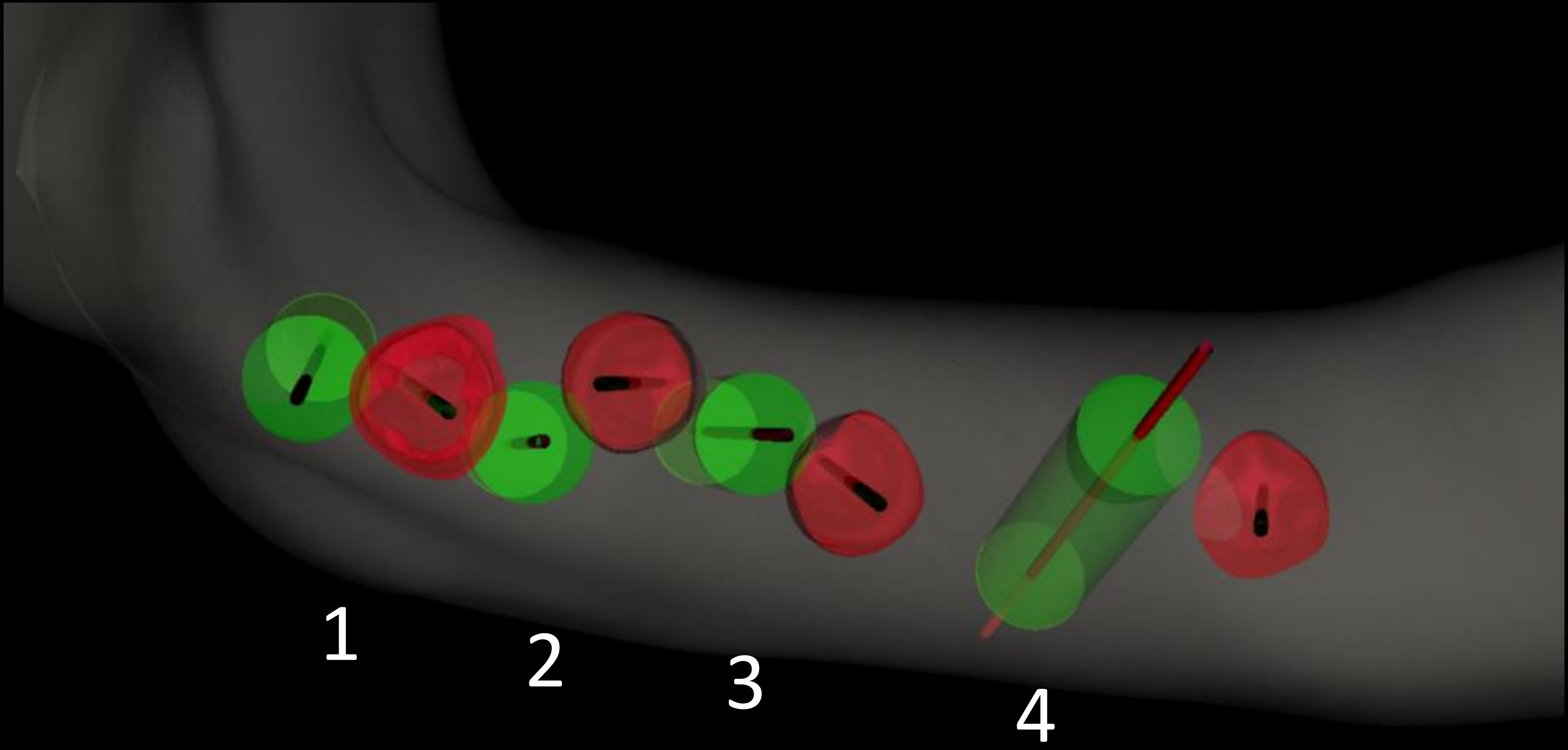
1
2
3
4

ION005 - INF

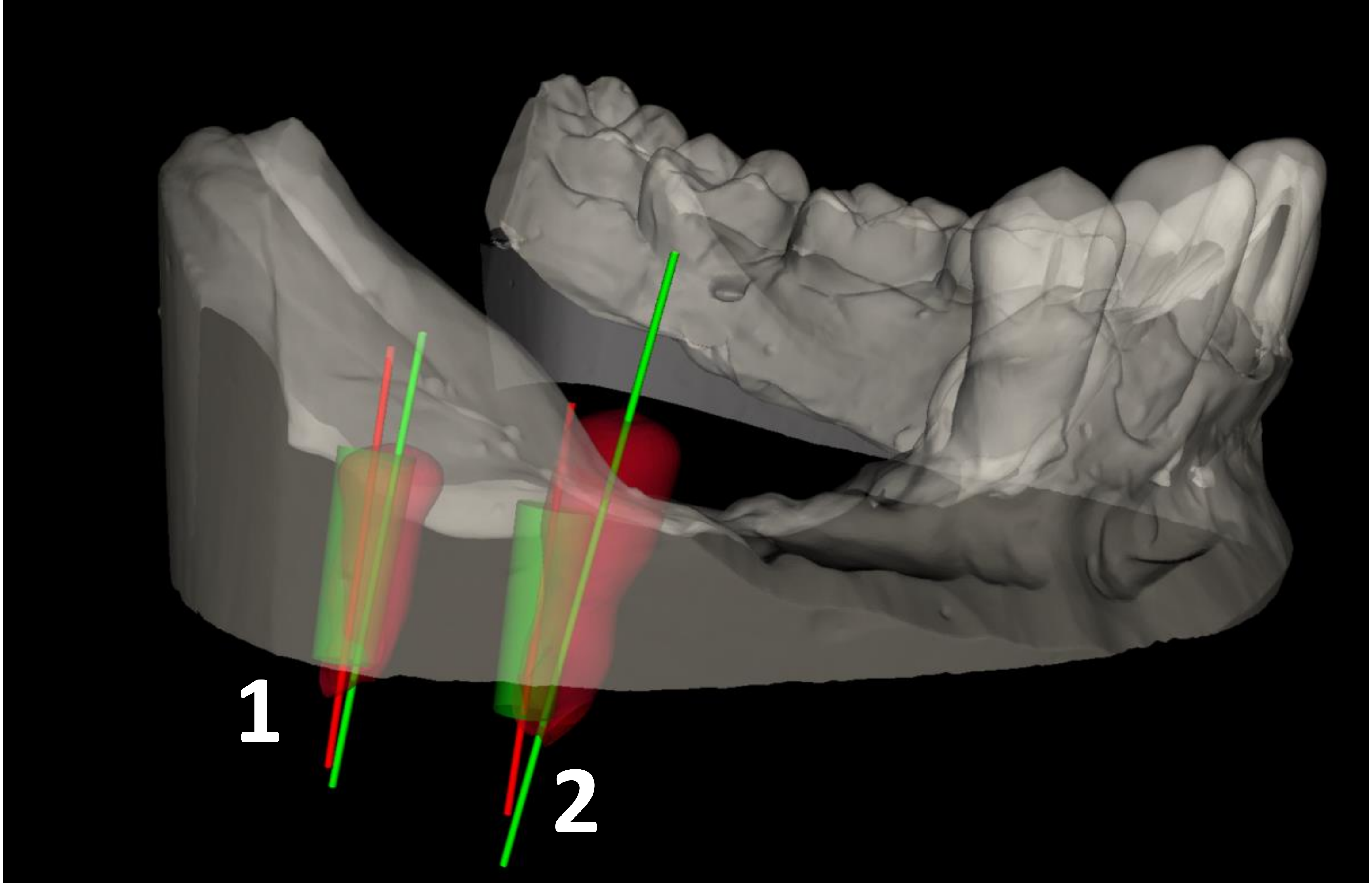
1
2

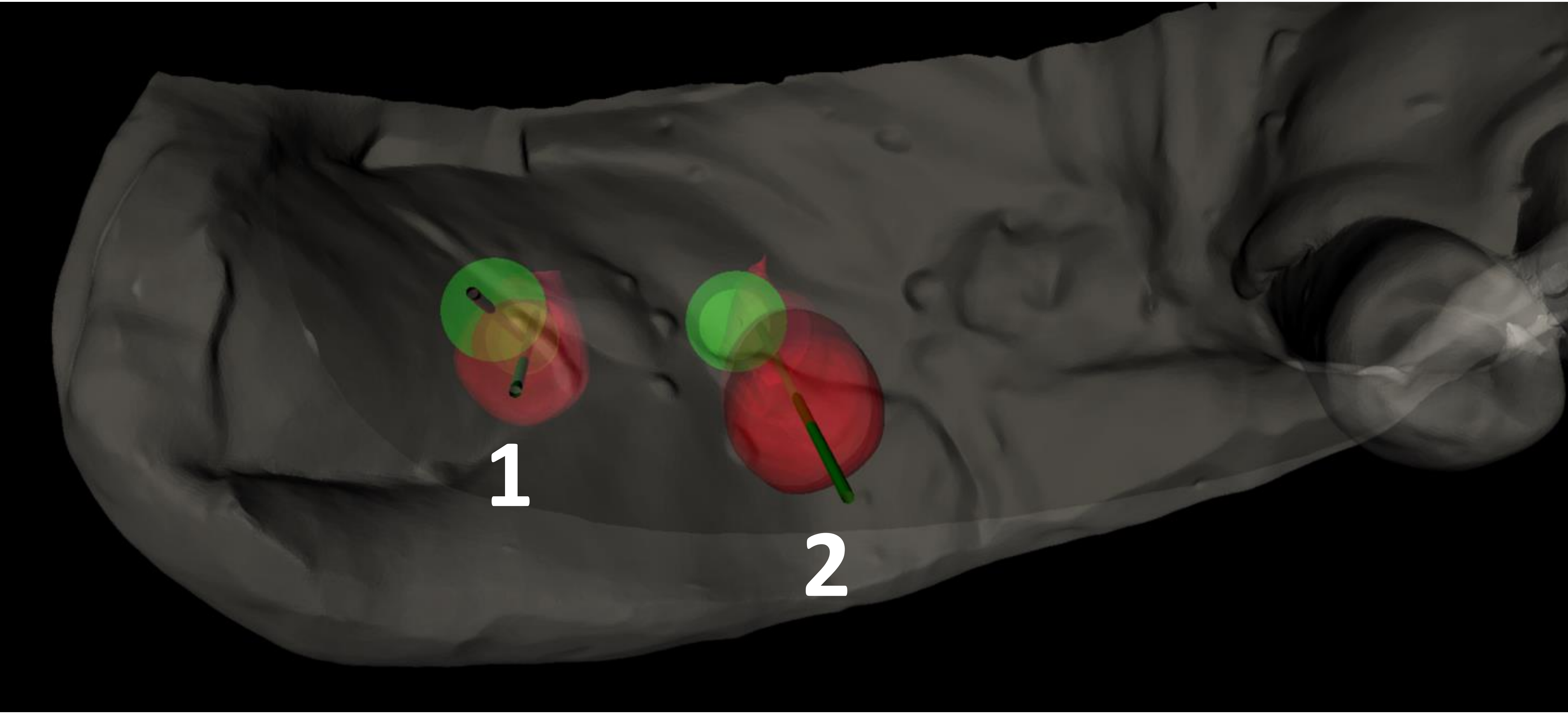
1
2

ION006 - INF

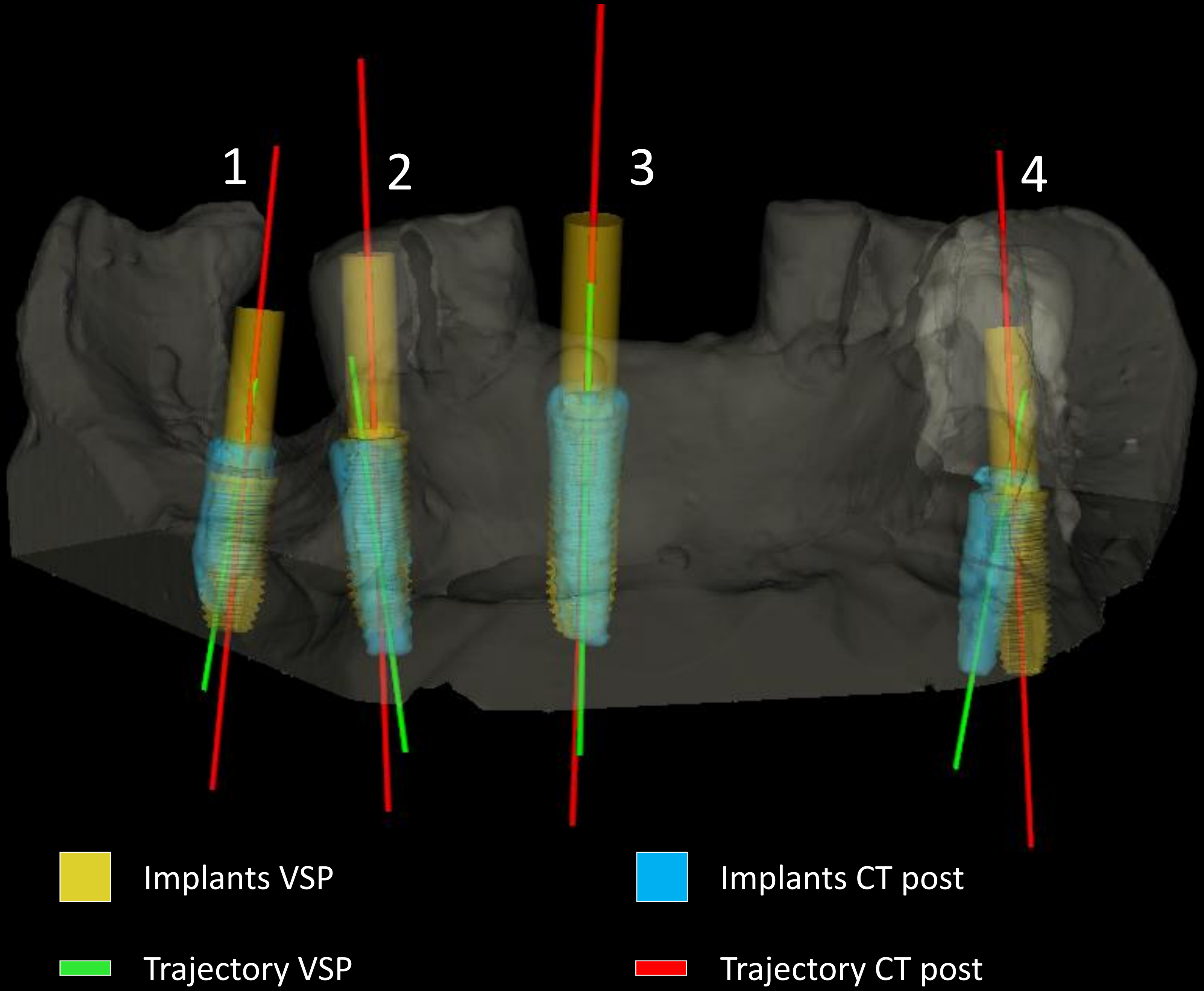
1
2
3
4
Implants VSP
Implants CT post
Trajectory VSP
Trajectory CT post

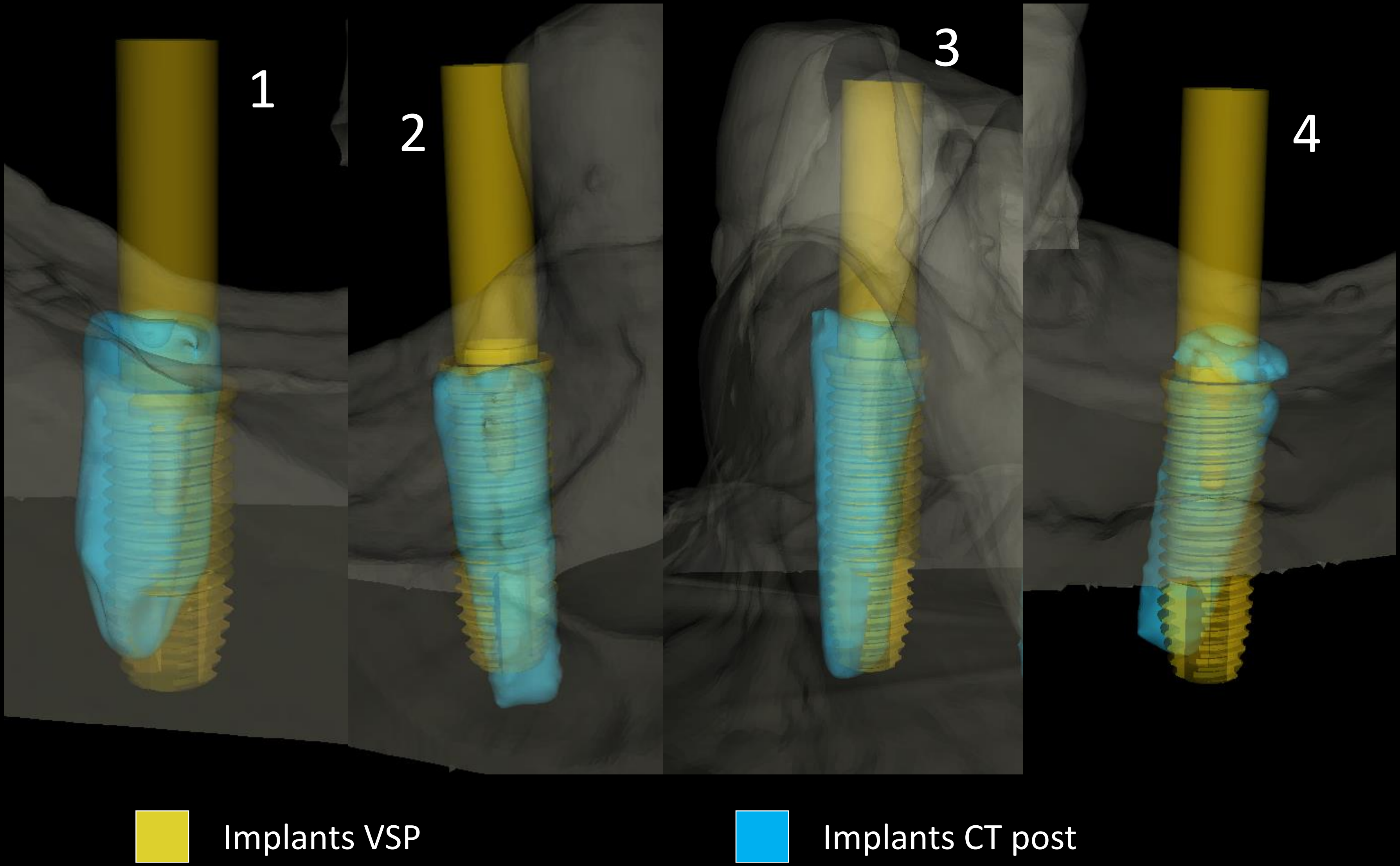
1
2
3
4
Implants VSP
Implants CT post

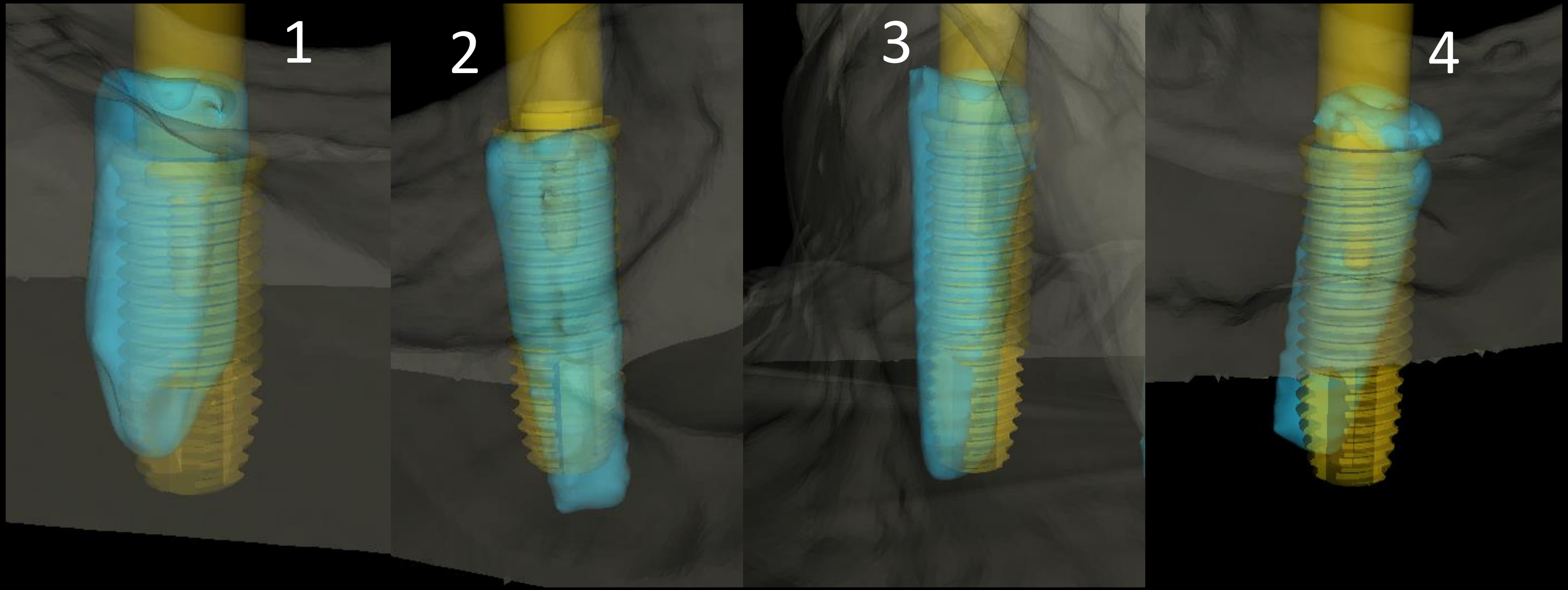
1
2
3
4

ION006 - SUP

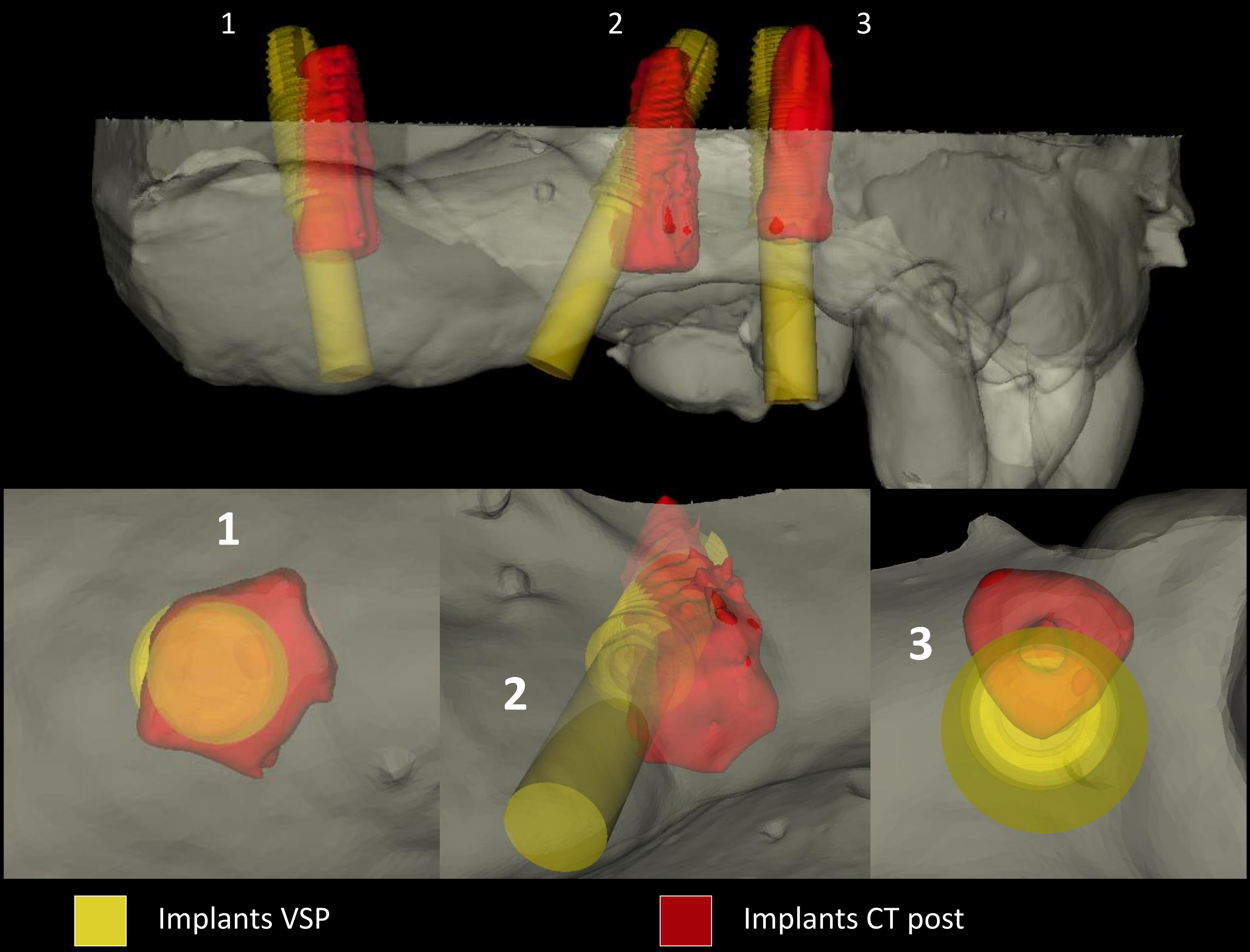
1
2
3
1
2
3
Implants VSP
Implants CT post

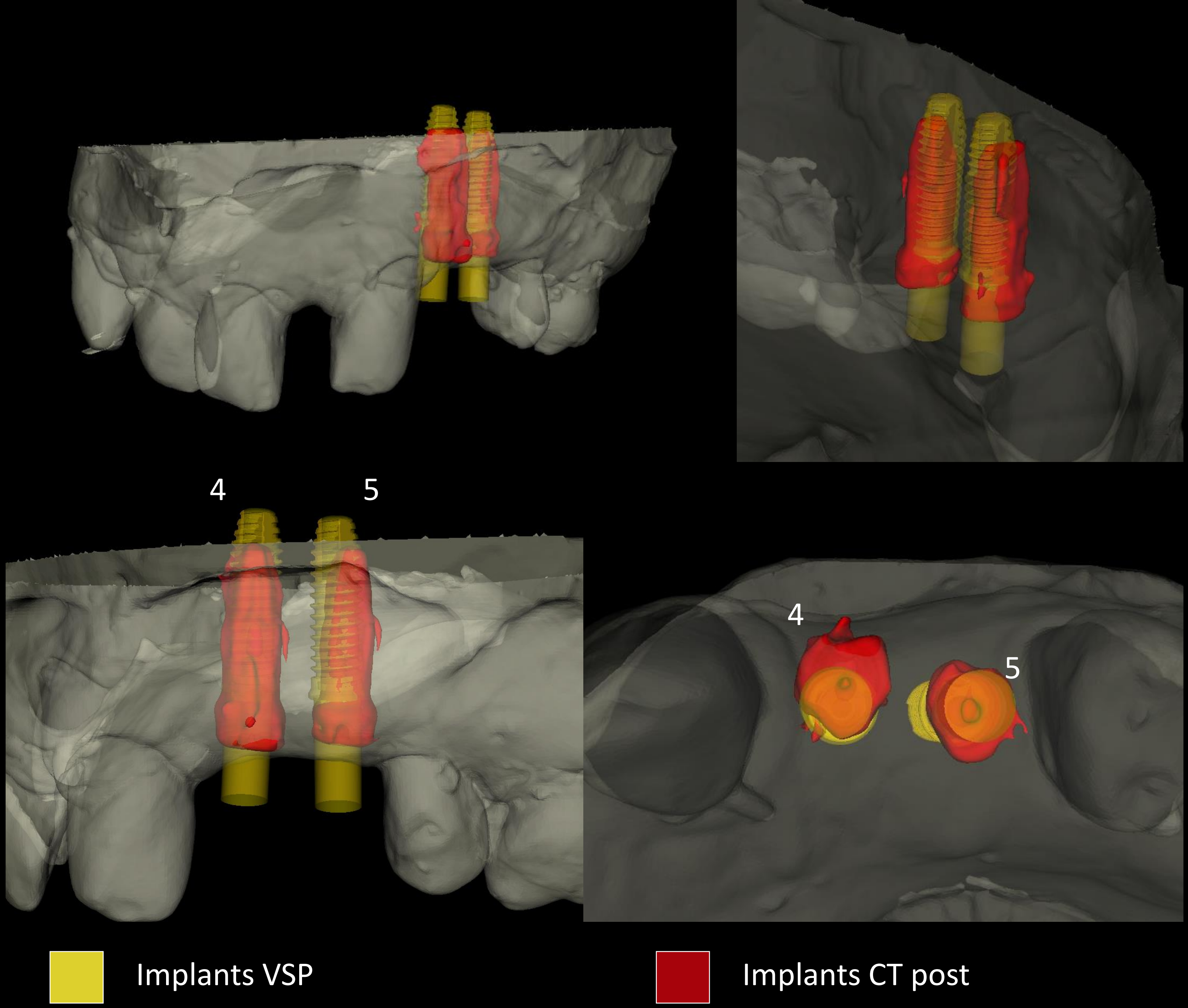
4
5
4
5
Implants VSP
Implants CT post

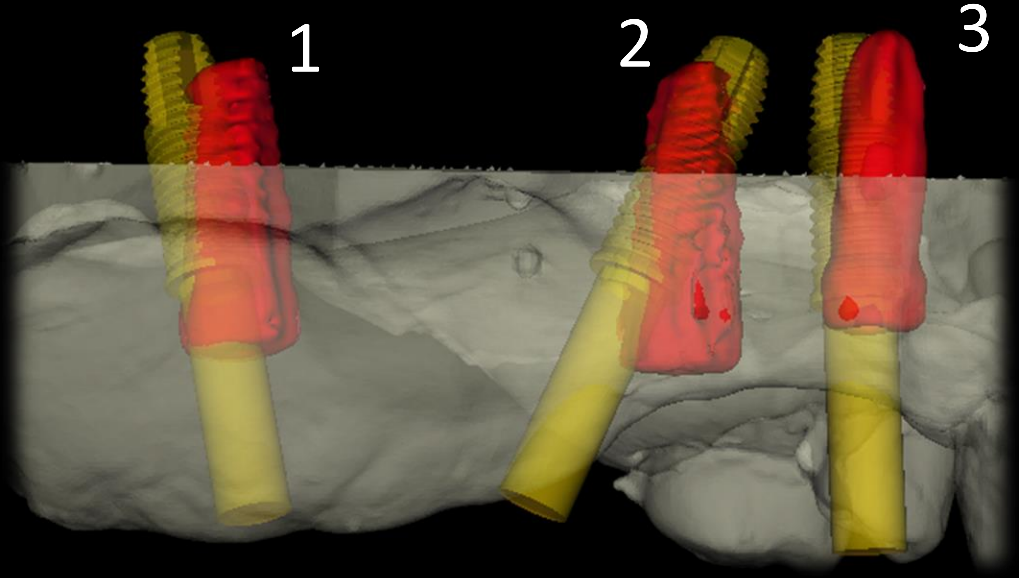
1
2
3

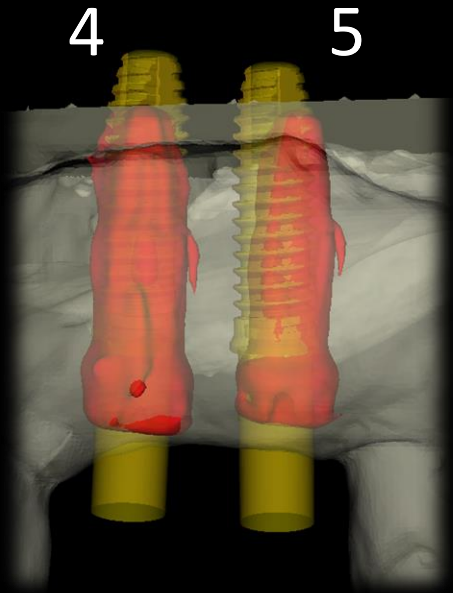
4
5

ION007 - SUP

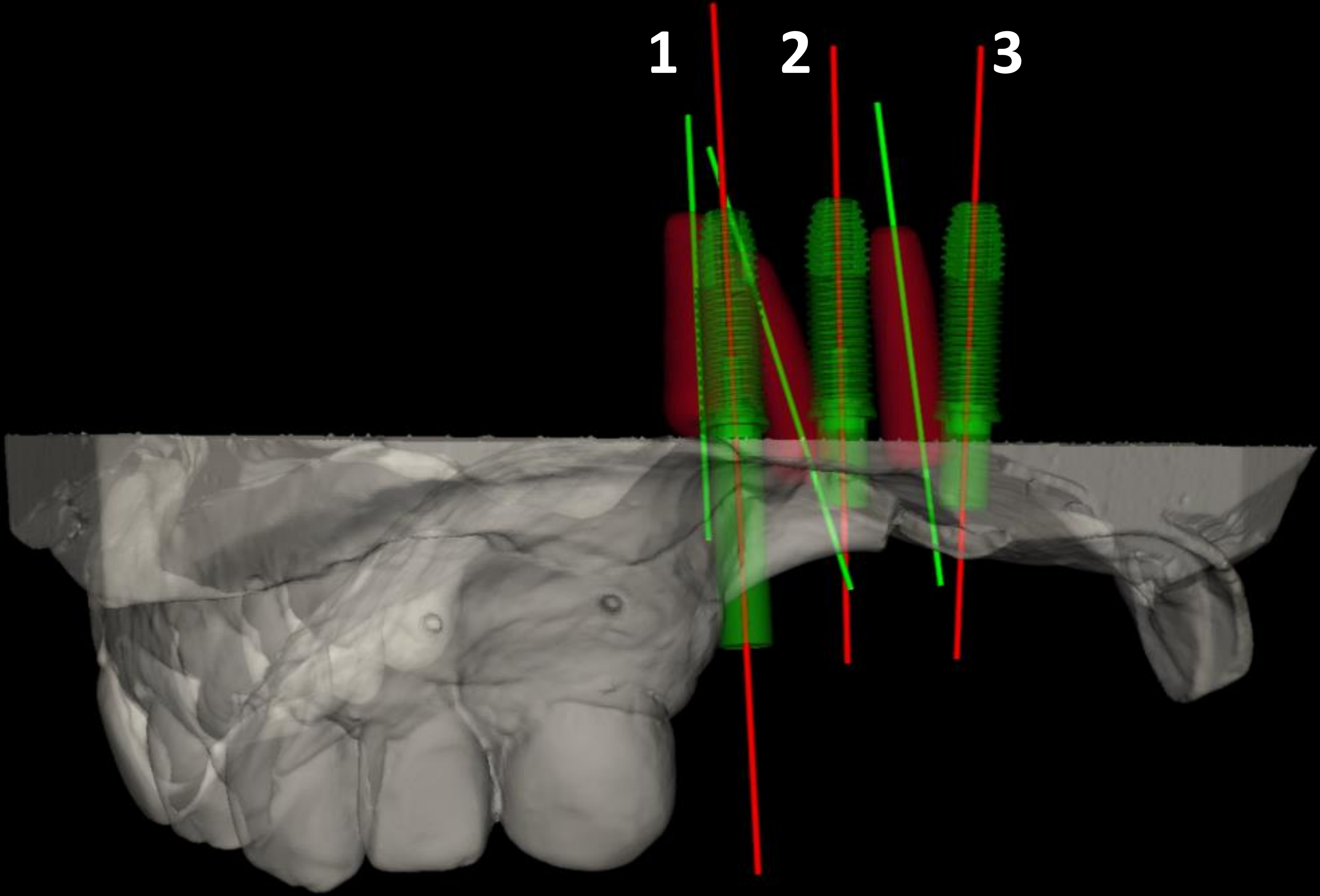
1
2
3

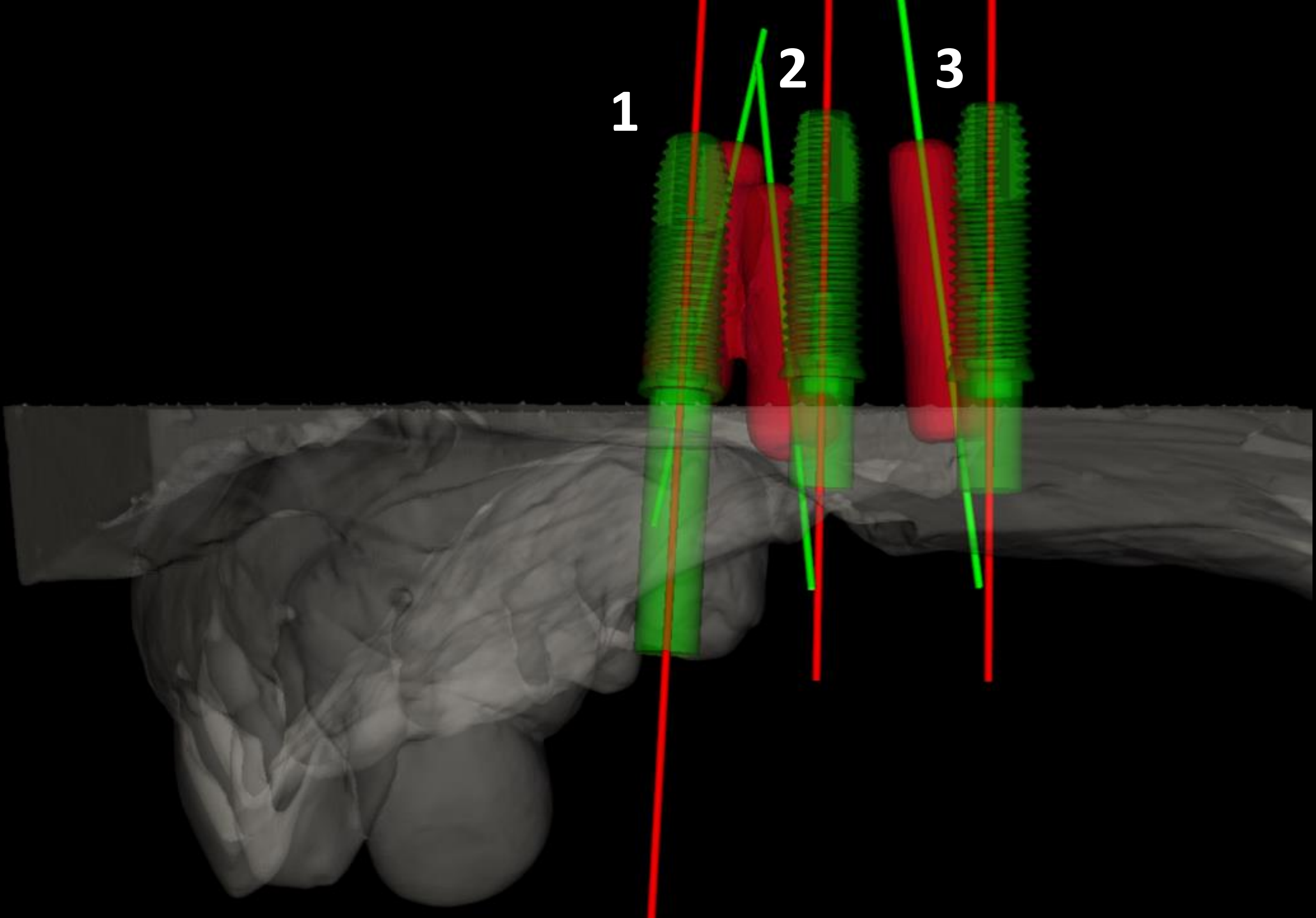
1
2
3

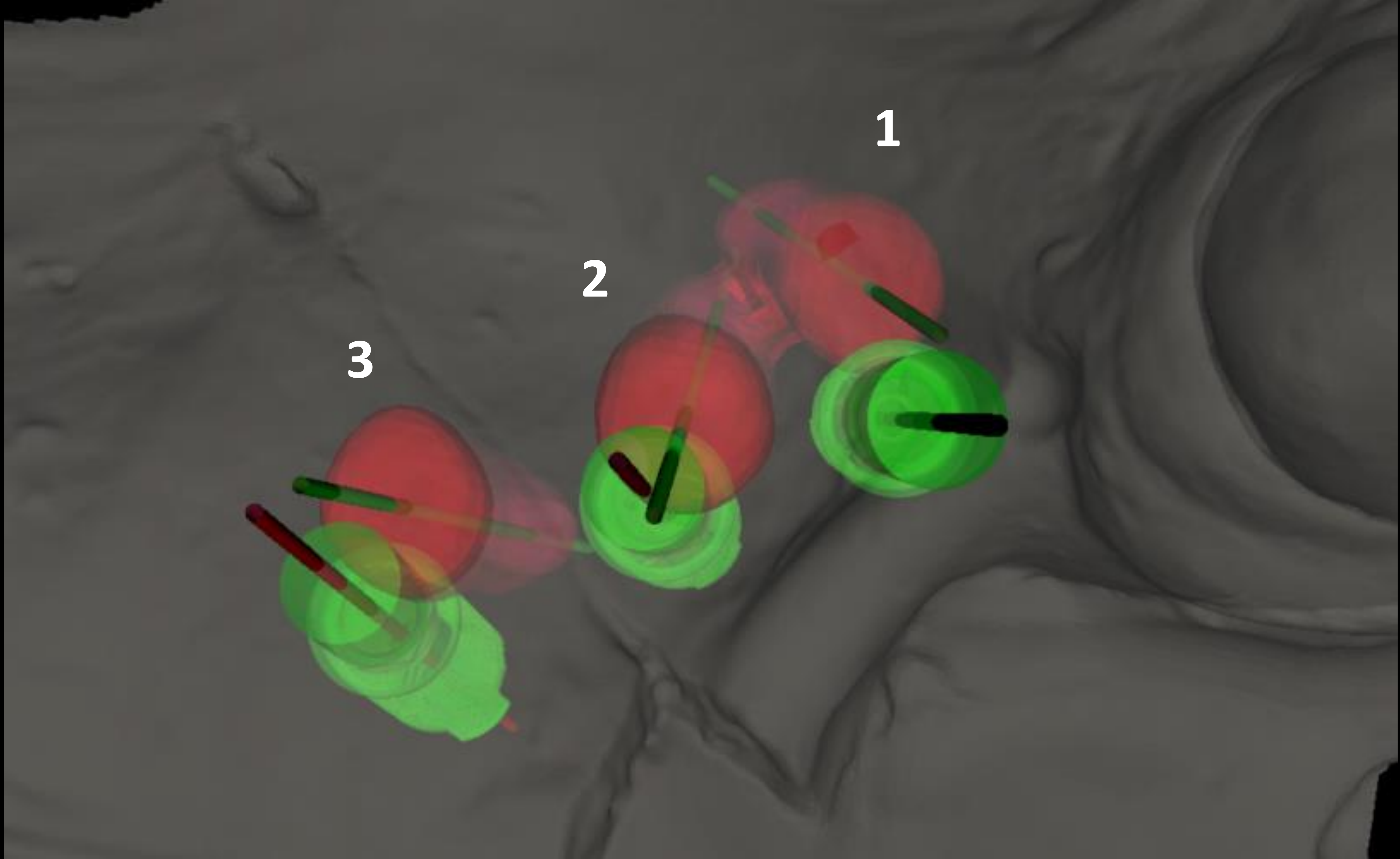
1
2
3

ION008 - INF

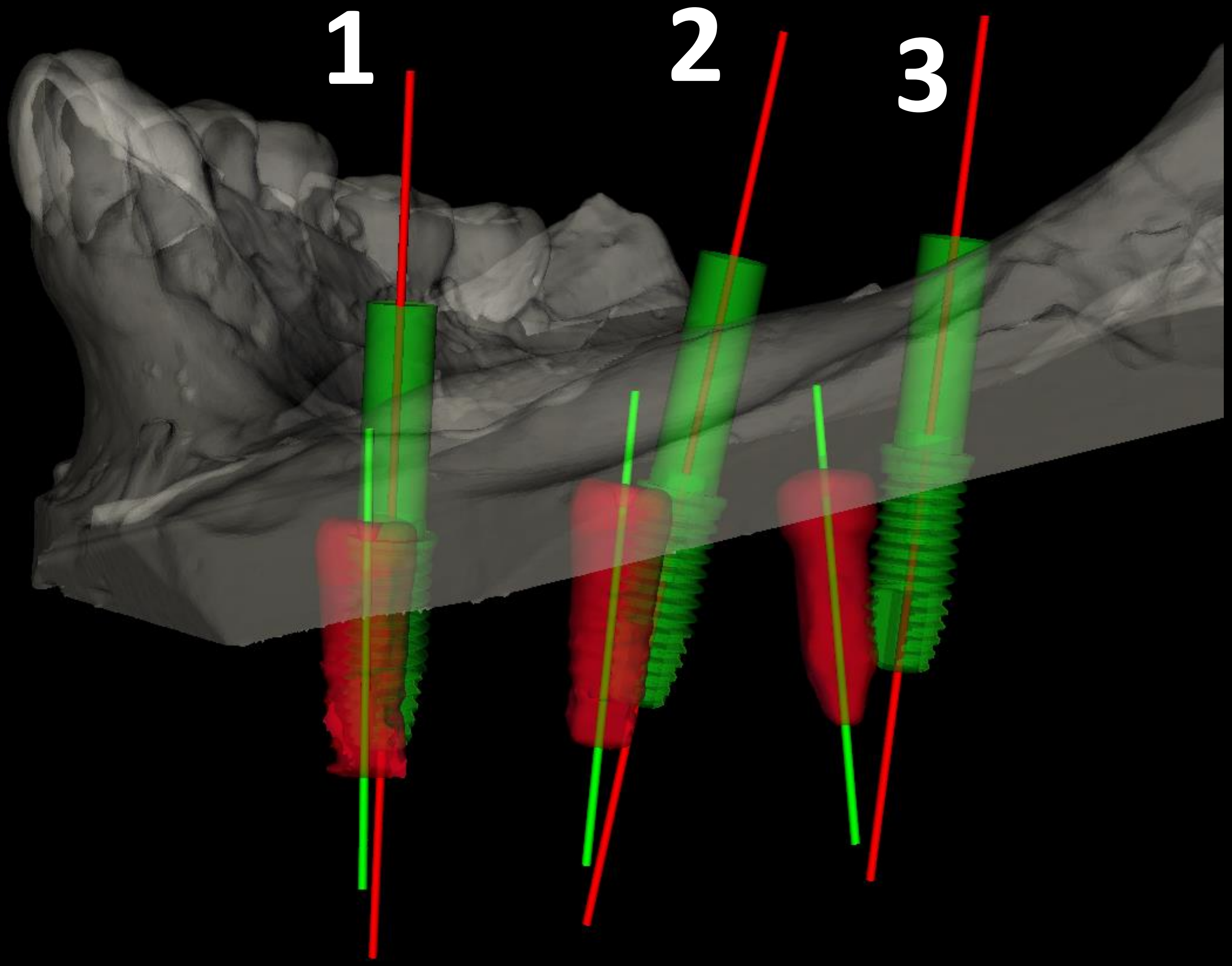
1
2
3

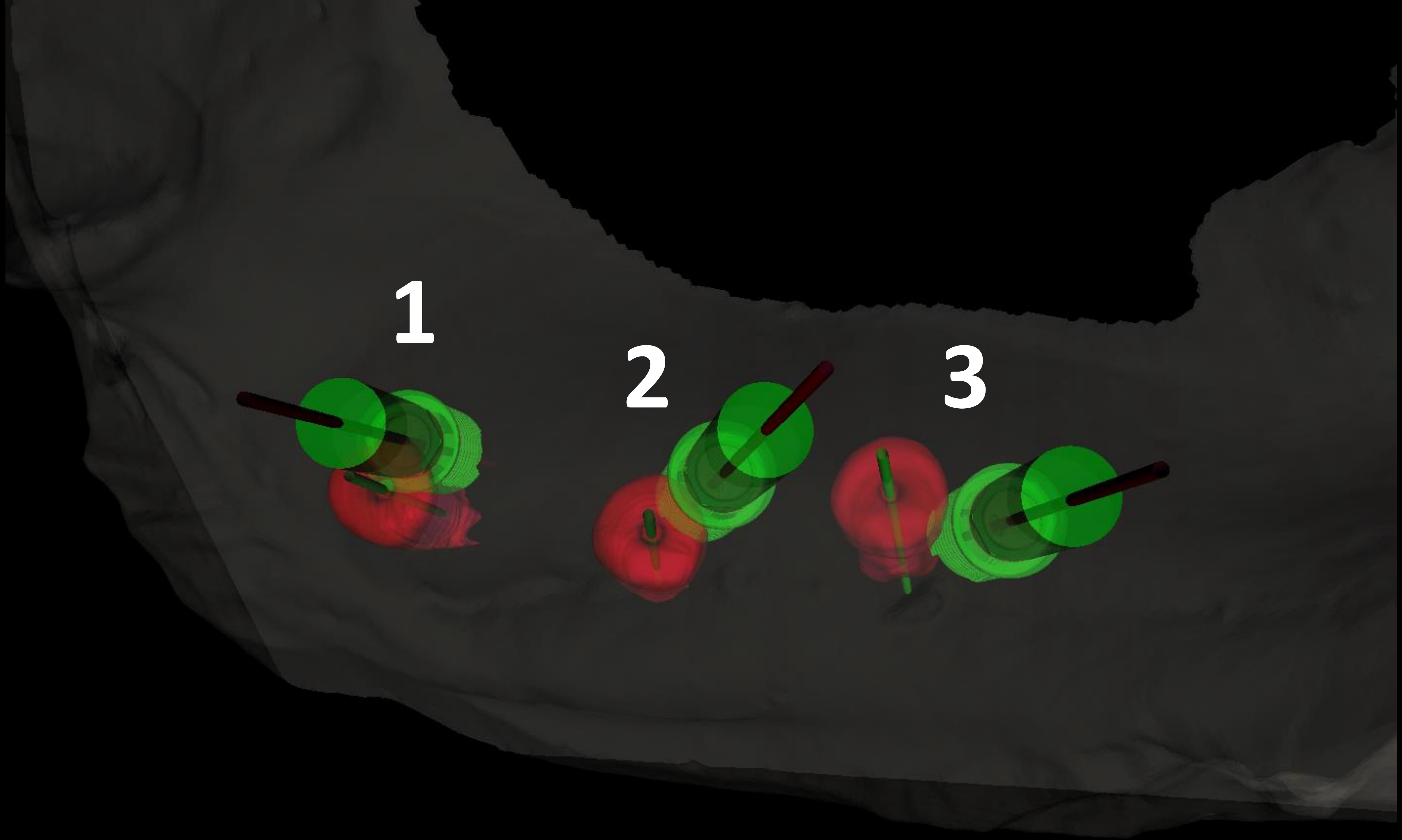
1
2
3

ION009 - INF

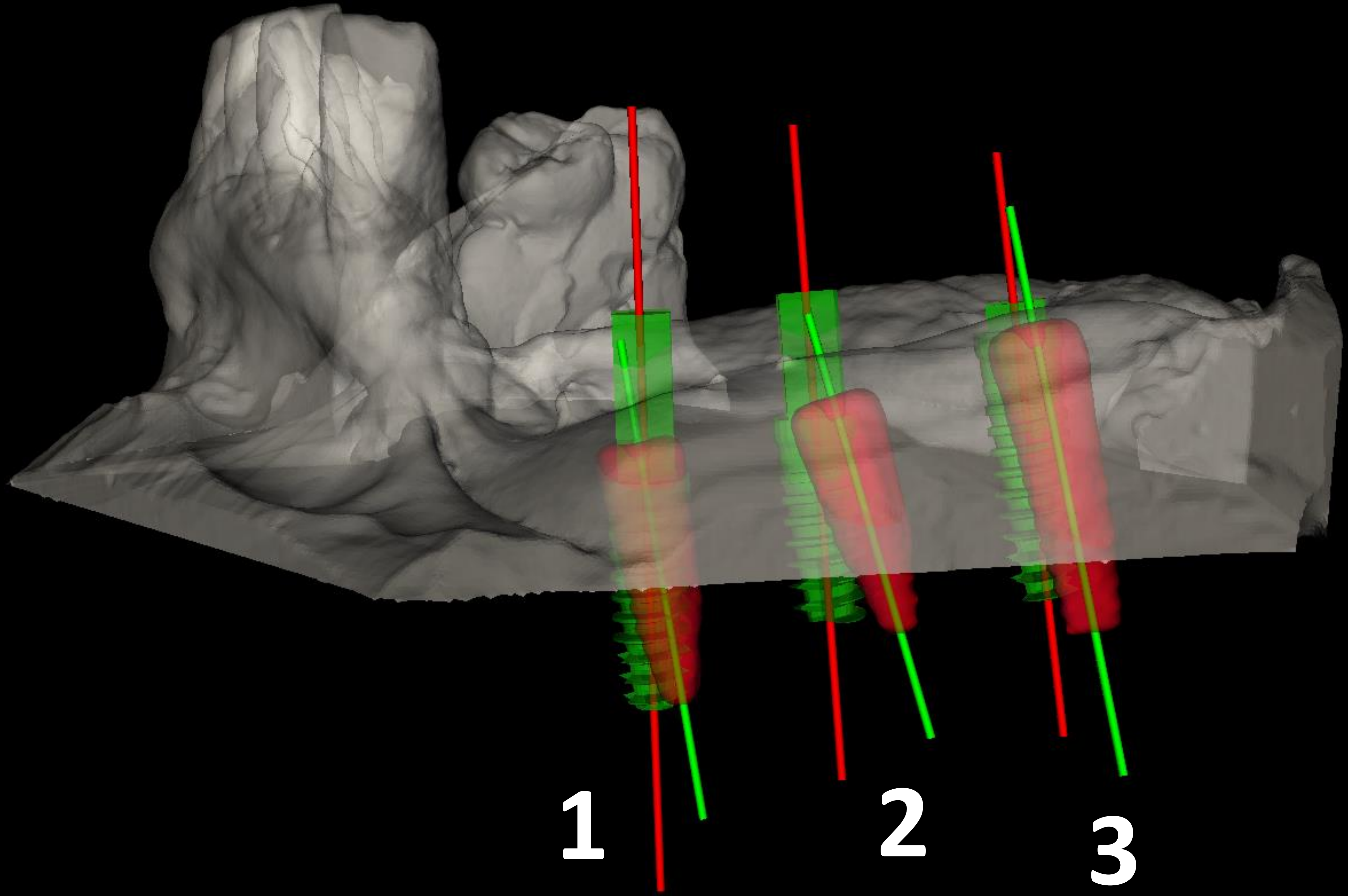
1
2
3

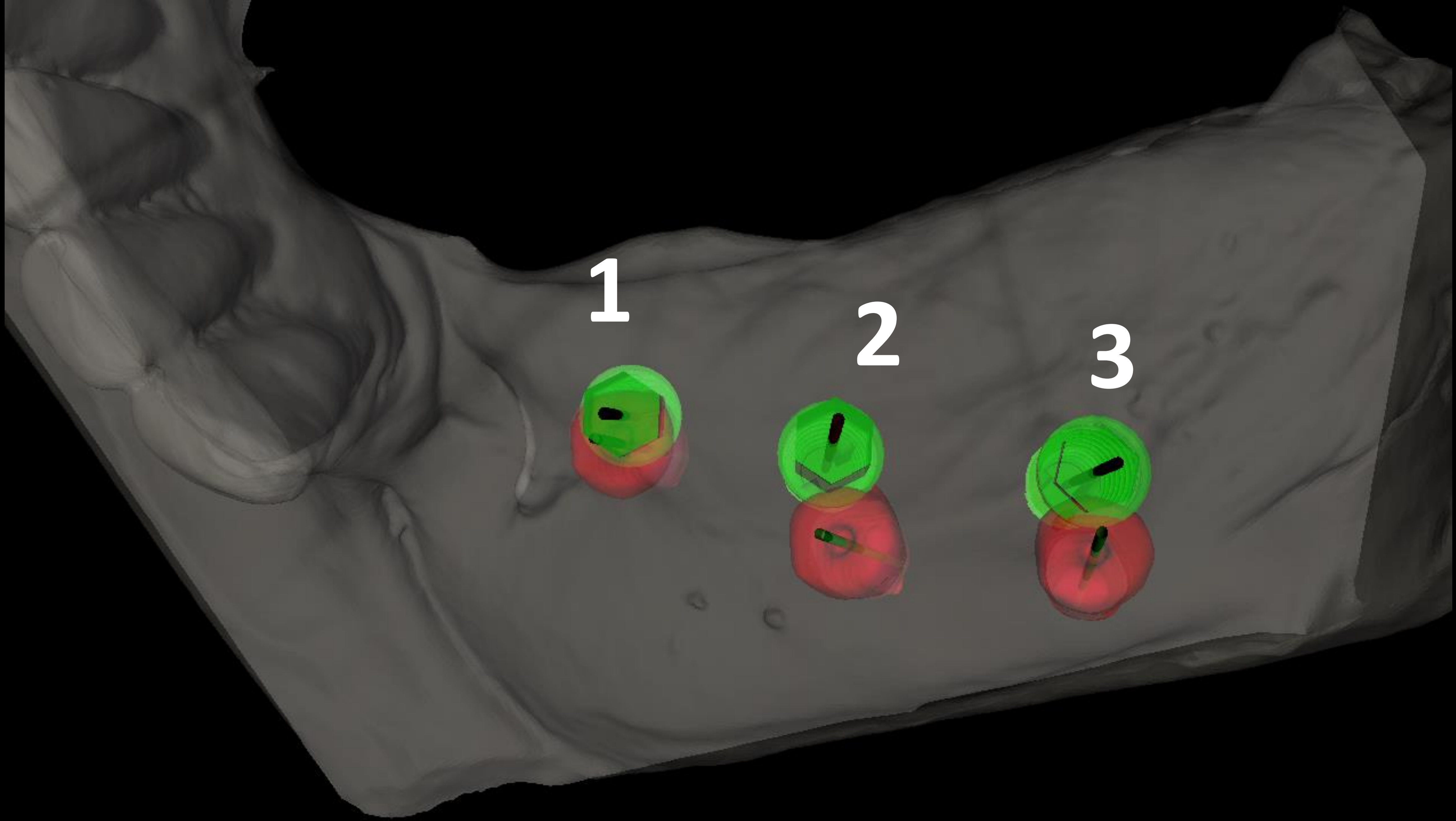
1
2
3

ION009 – SUP

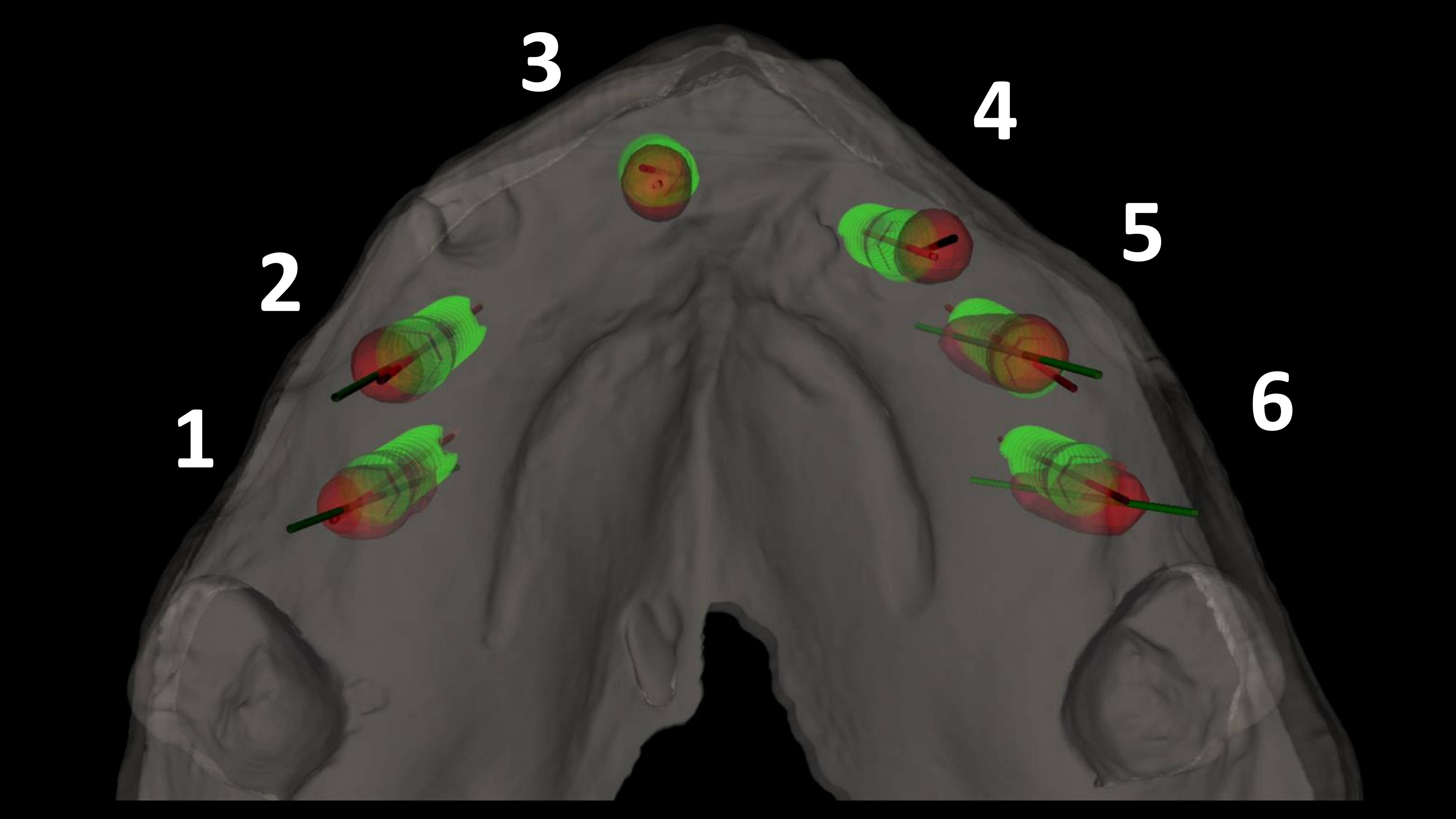
3
4
5
2
6
1

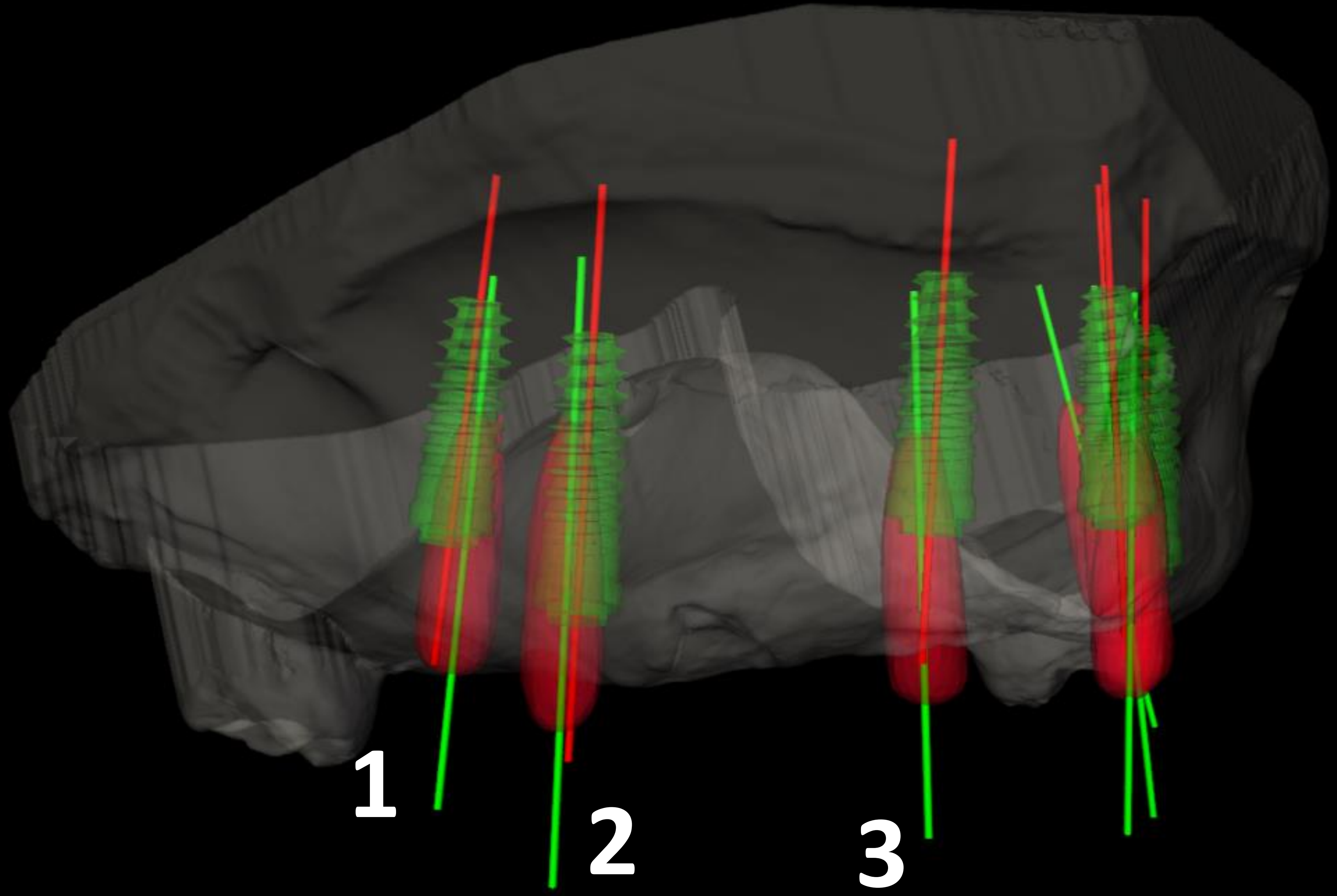
1
2
3

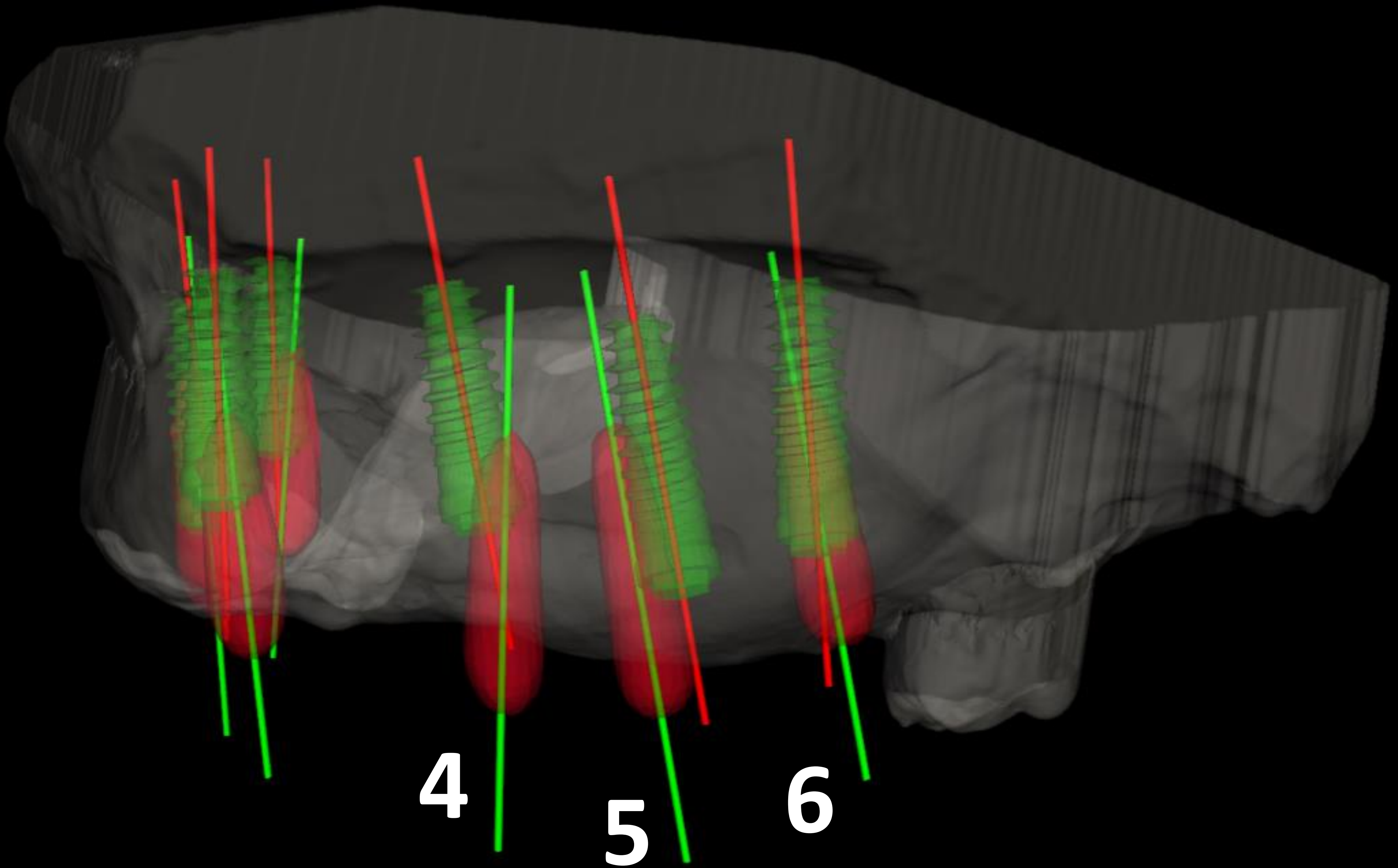
4
5
6

ION0010 - INF

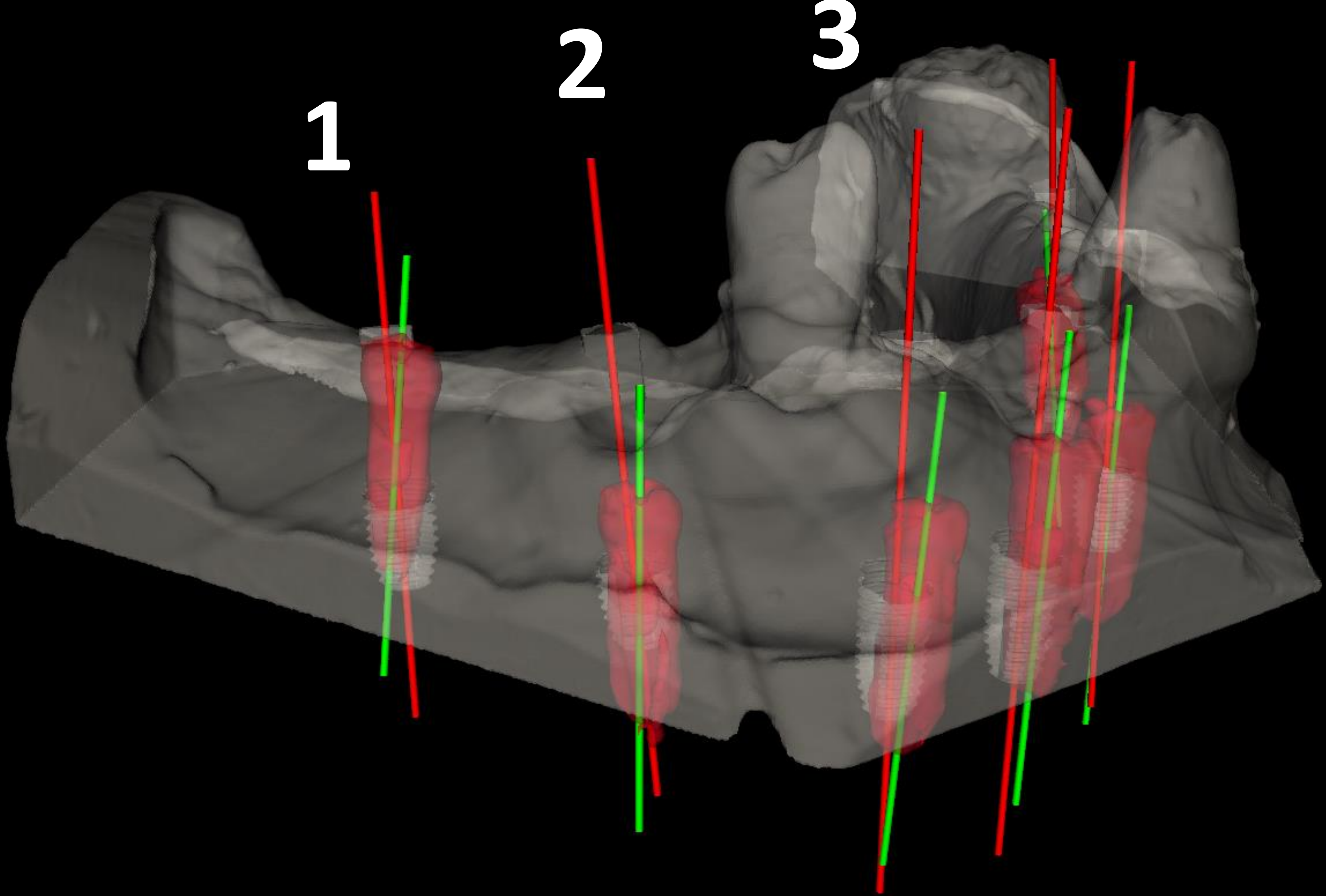
1
2
3

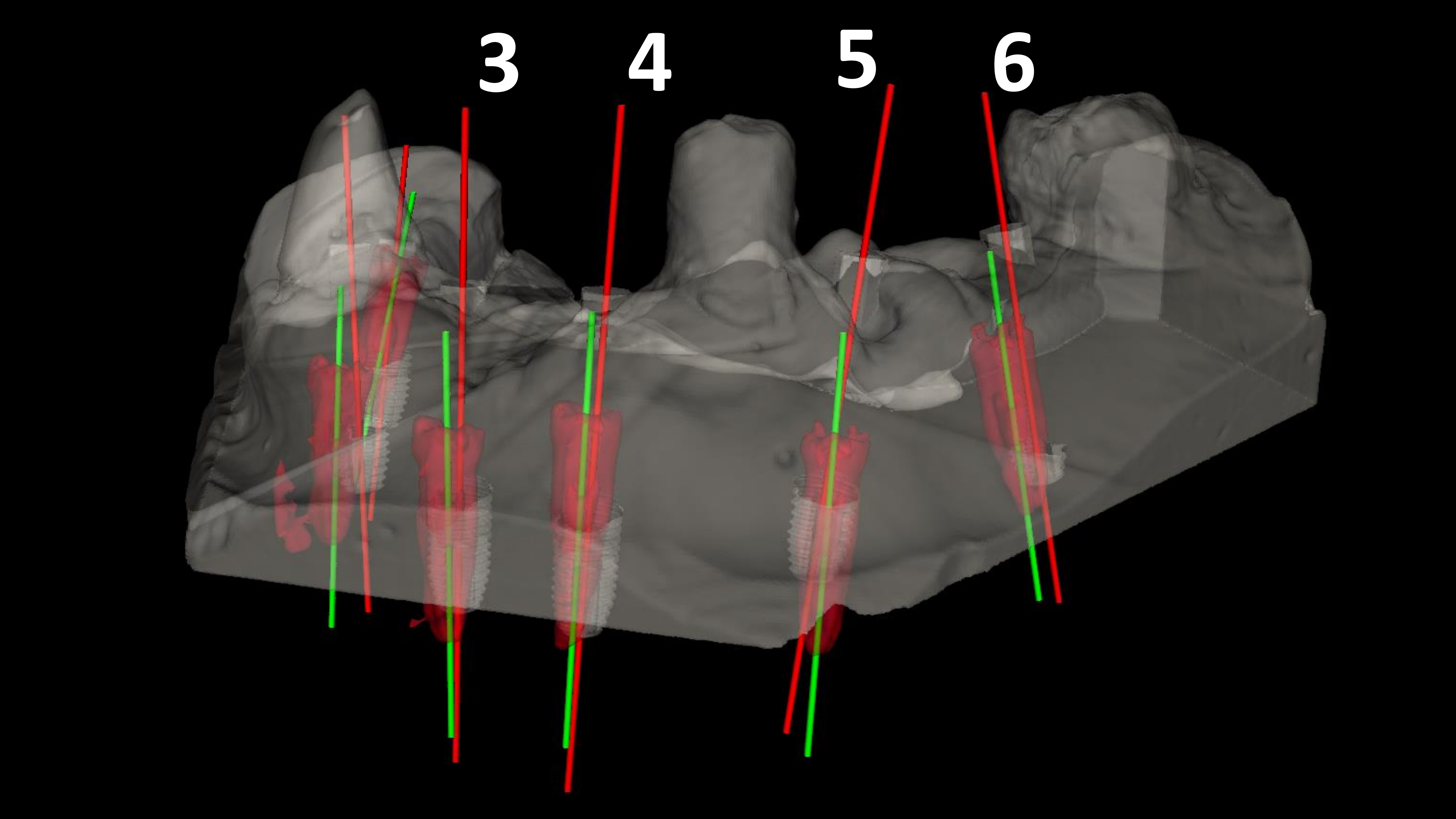
3
4
5
6

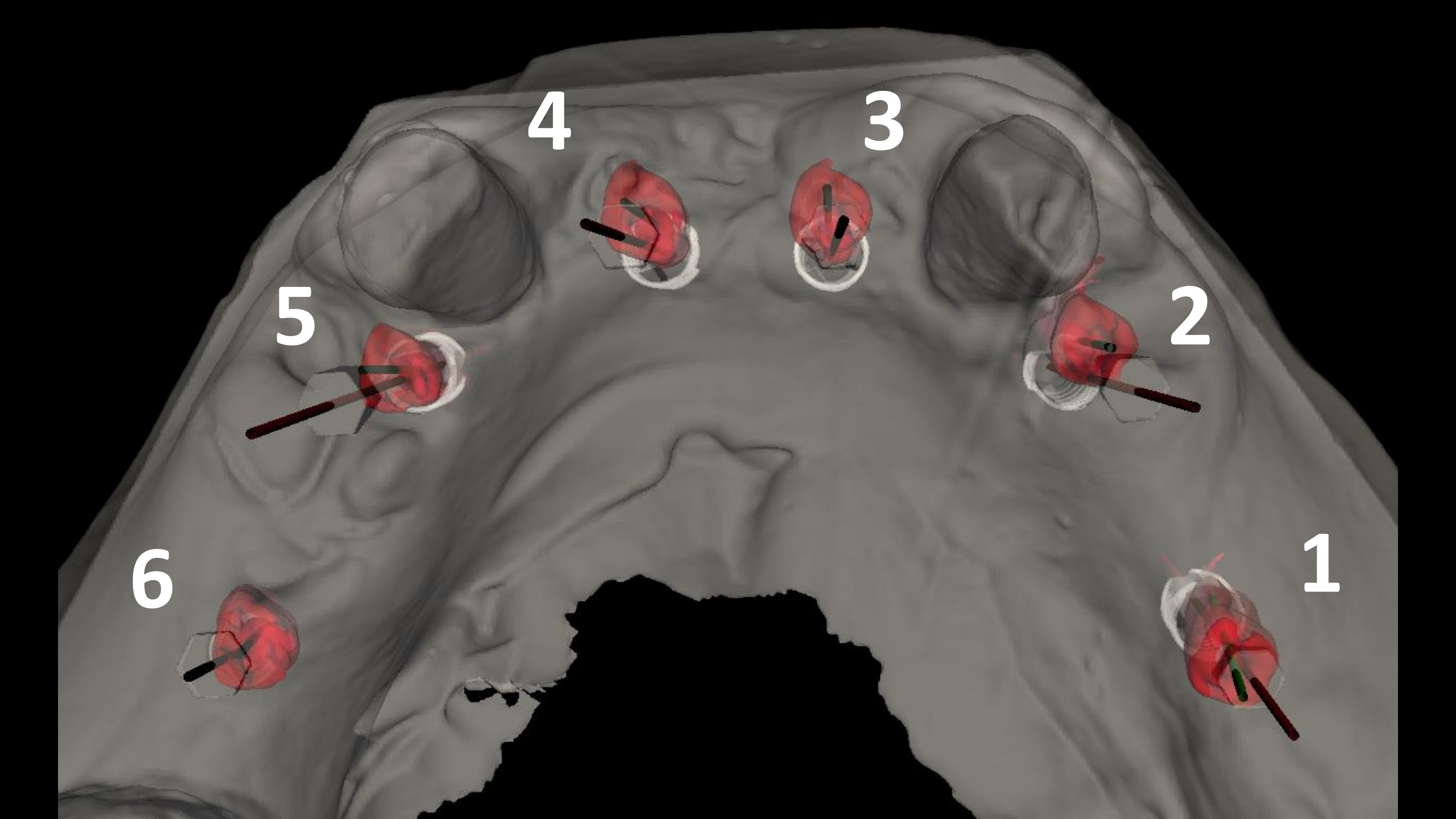
4
3
5
2
6
1

ION0010 - SUP

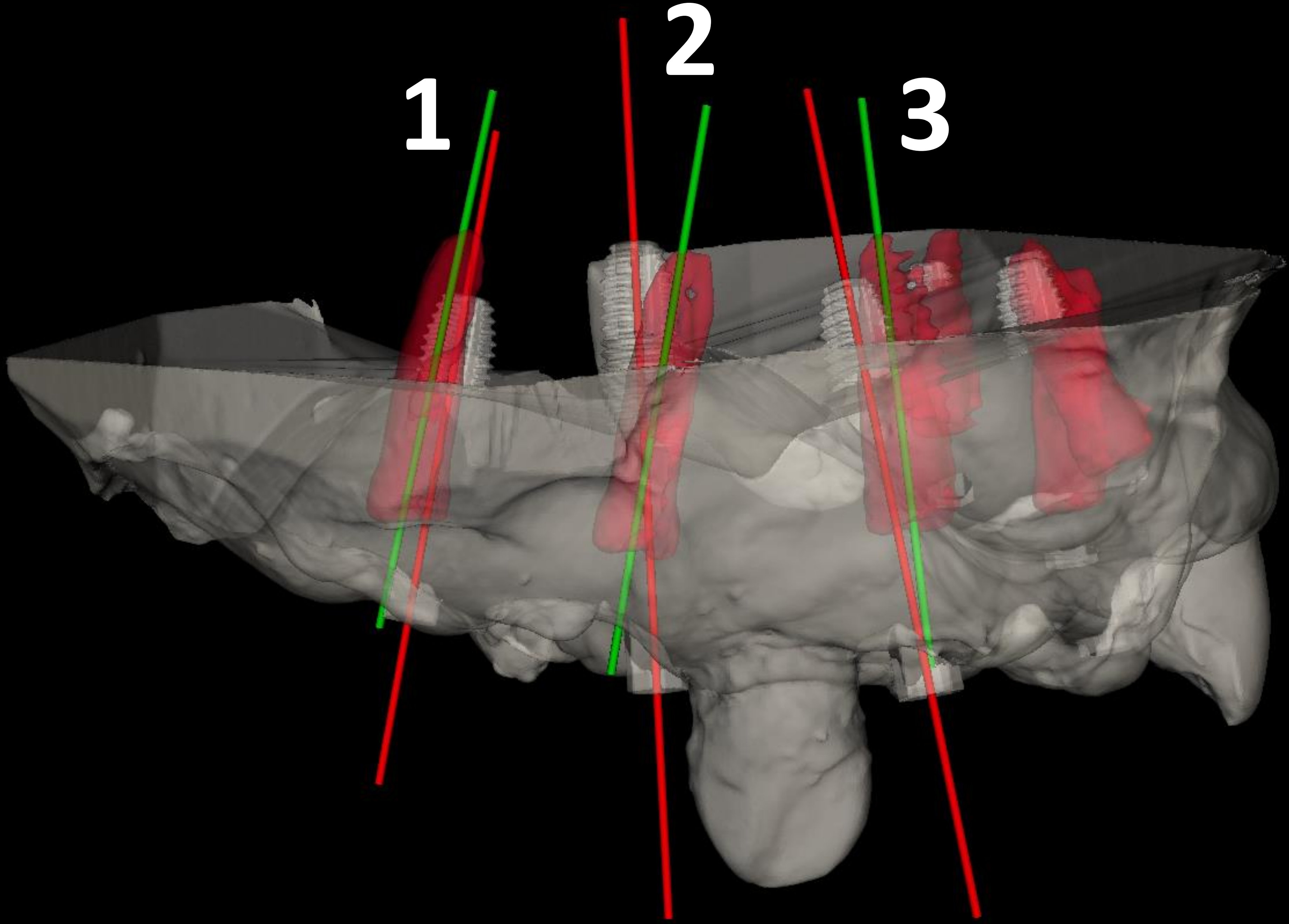
1
2
3

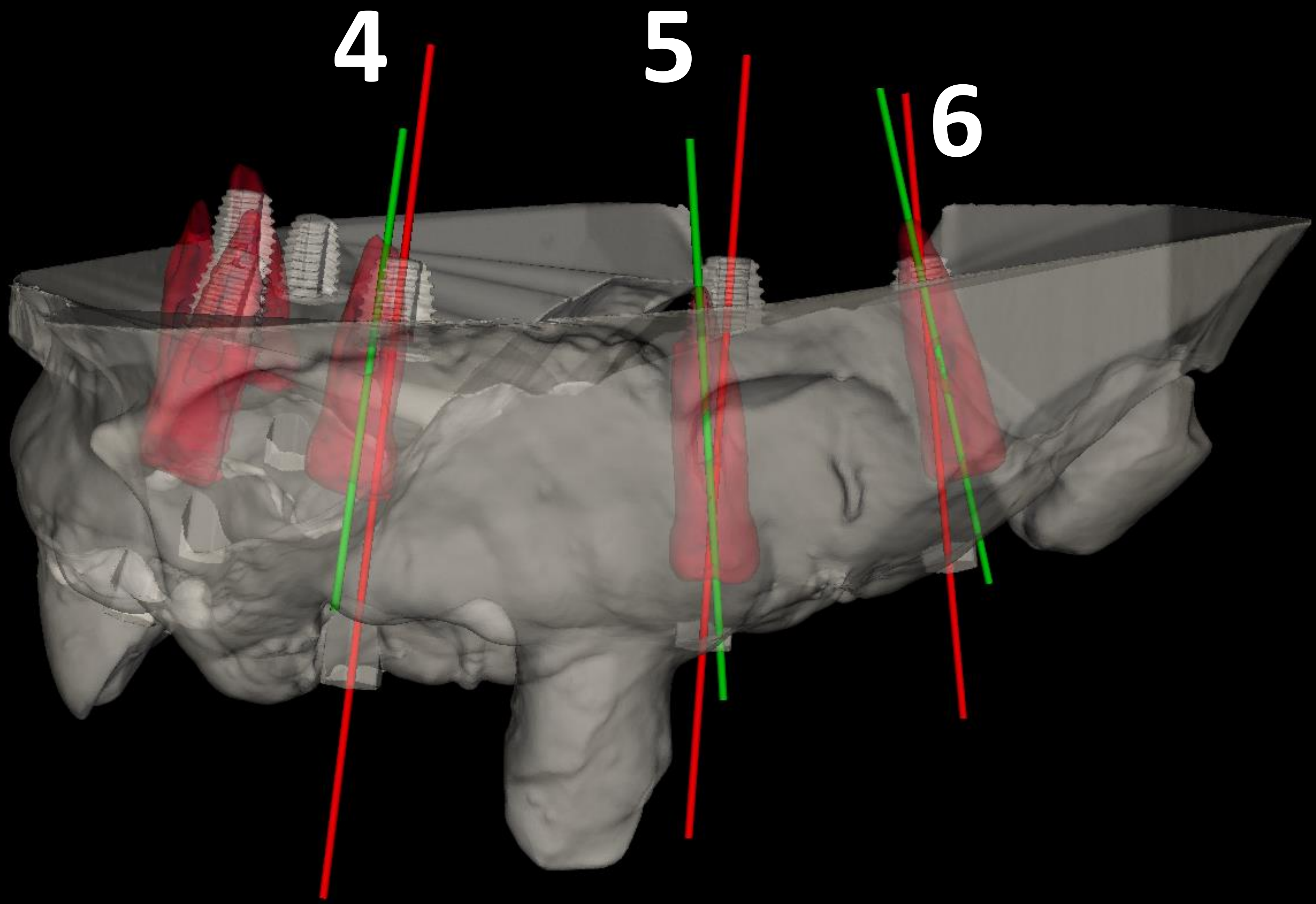

4
5
6

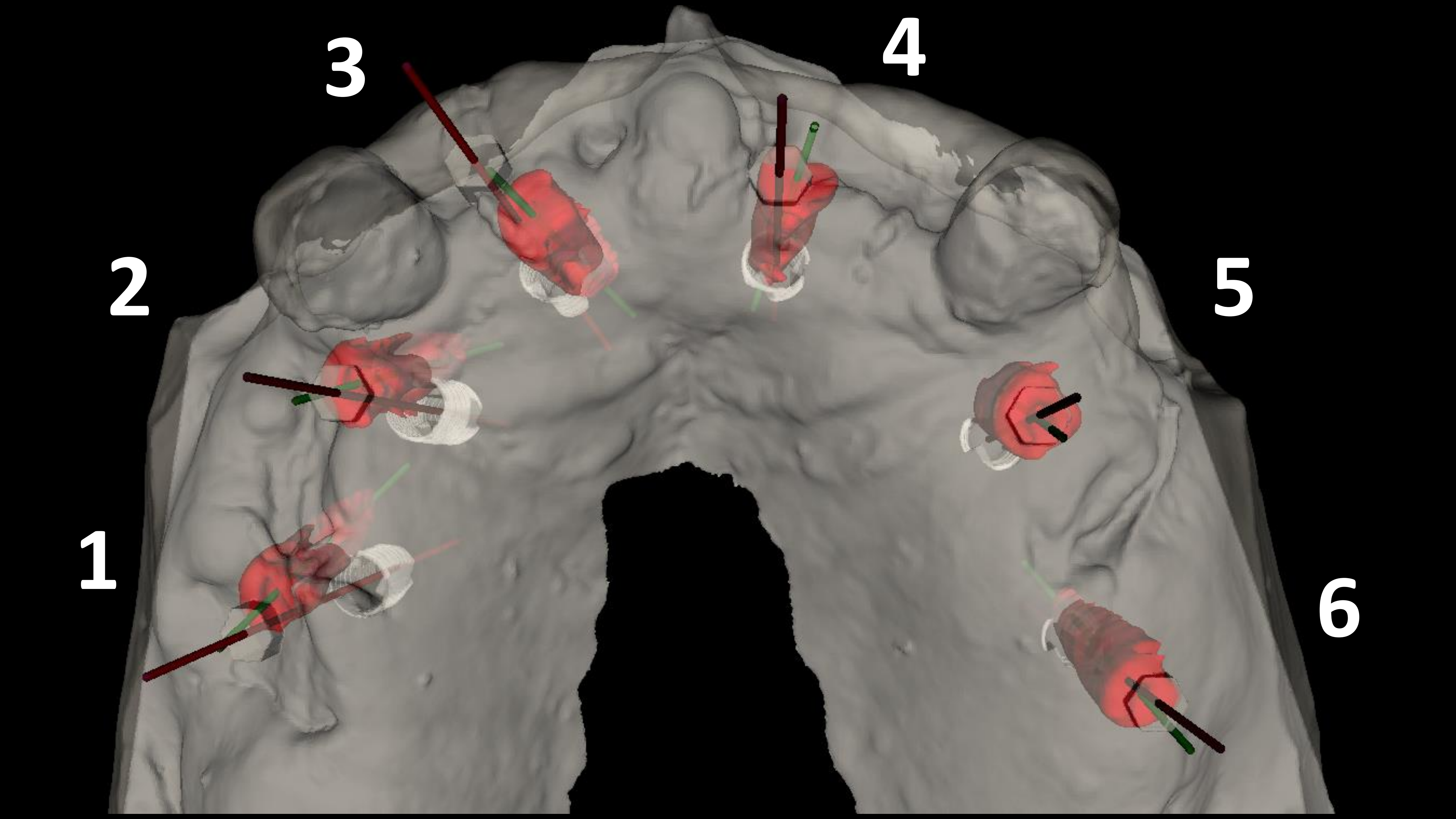
3
4
2
5
1
6

ION0011 - SUP

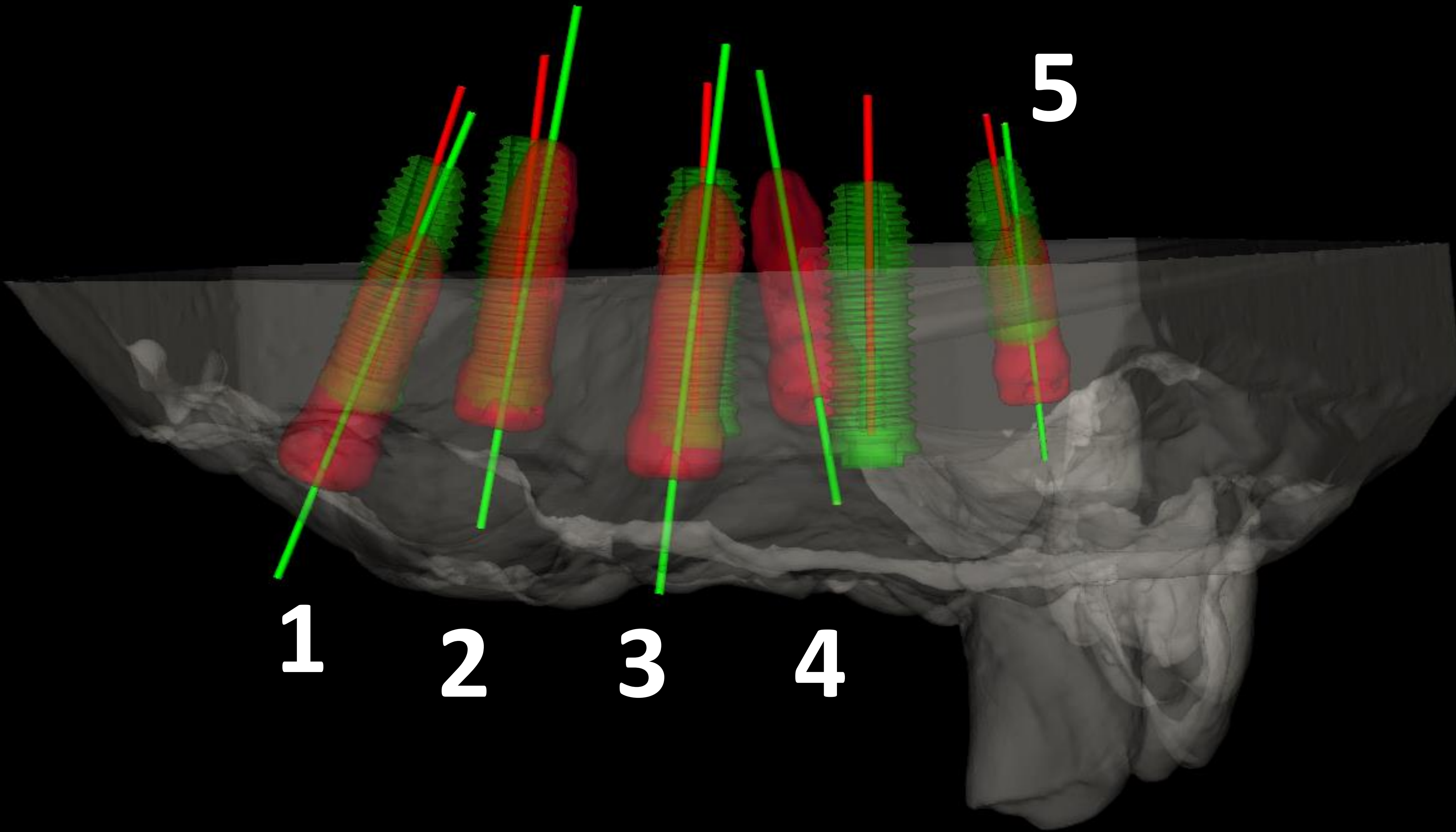
5
1
2
3
4

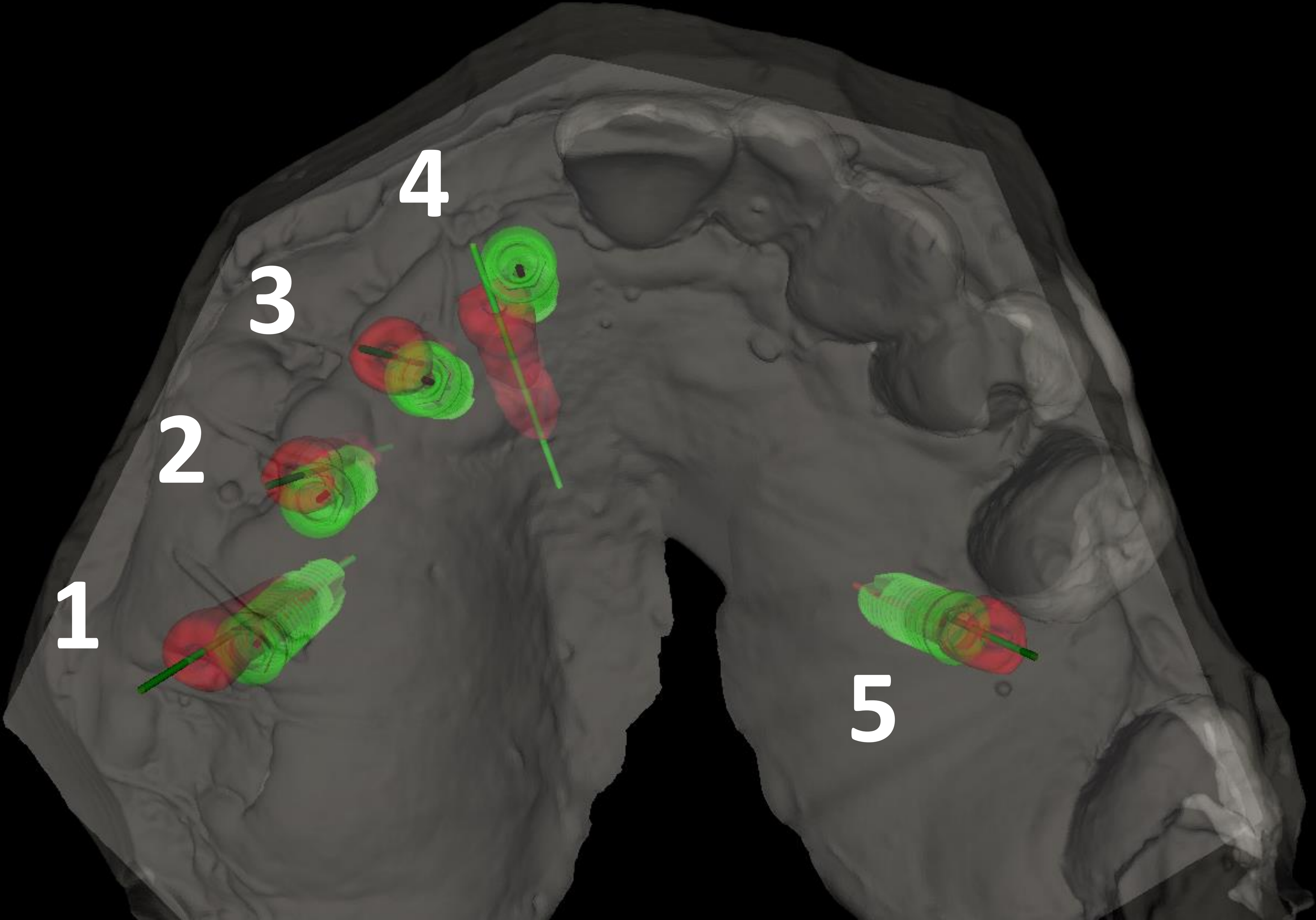

4
3
2
1
5

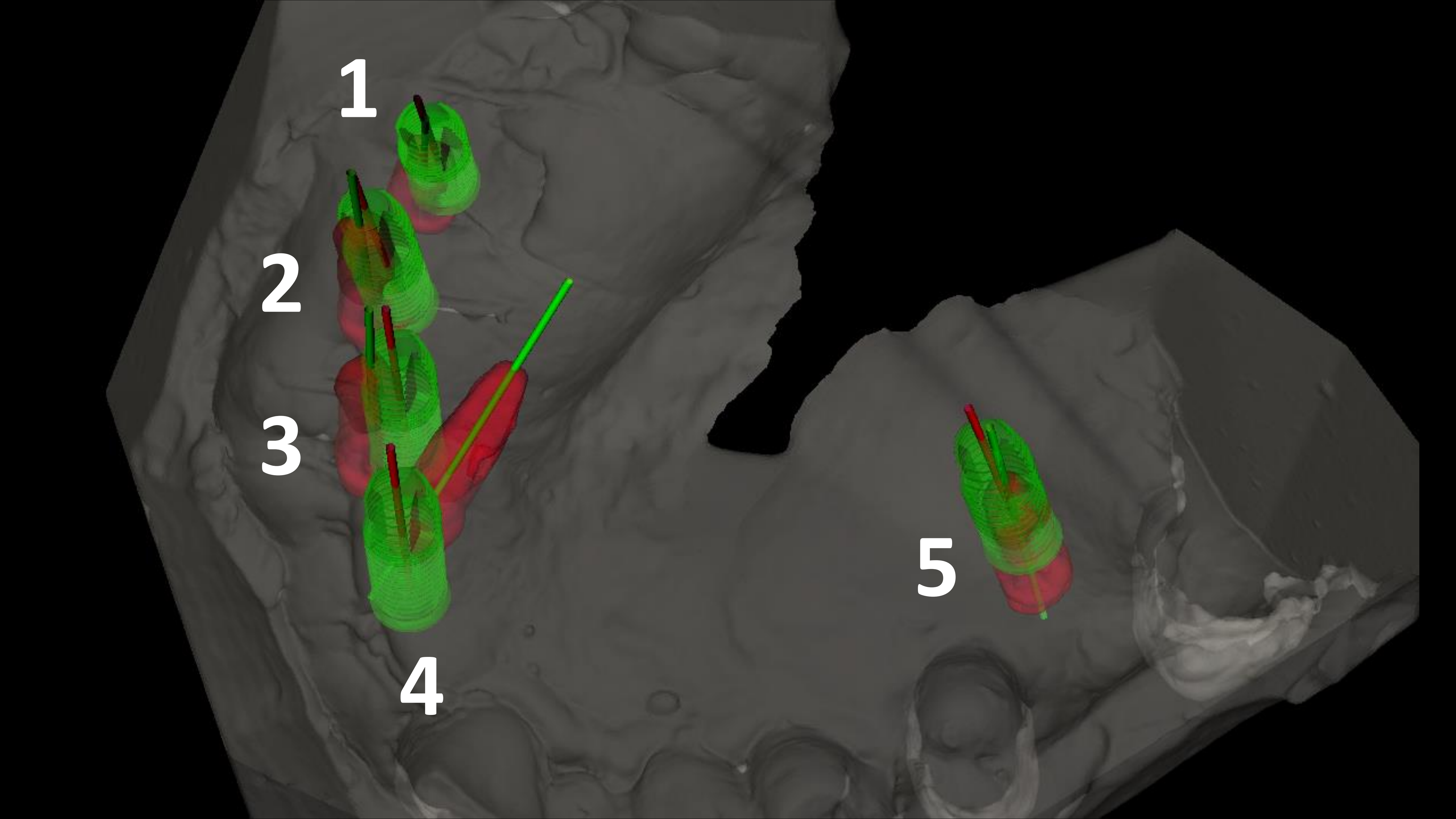
1
2
3
4
5
